# Practical tetrafluoroethylene fragment installation through a coupling reaction of (1,1,2,2-tetrafluorobut-3-en-1-yl)zinc bromide with various electrophiles

**DOI:** 10.3762/bjoc.14.213

**Published:** 2018-09-11

**Authors:** Ken Tamamoto, Shigeyuki Yamada, Tsutomu Konno

**Affiliations:** 1 Faculty of Molecular Chemistry and Engineering, Kyoto Institute of Technology, Matsugasaki, Sakyo-ku, Kyoto 606-8585, Japan

**Keywords:** acid chlorides, cross-coupling, iodoarenes, tetrafluoroethylene fragment, thermally stable zinc reagent

## Abstract

(1,1,2,2-Tetrafluorobut-3-en-1-yl)zinc bromide was prepared by insertion of the zinc–silver couple into the CF_2_–Br bond of commercially available 4-bromo-3,3,4,4-tetrafluorobut-1-ene in DMF at 0 °C for 0.5 h, The resultant polyfluorinated zinc reagent was found to be thermally stable at ambient temperature and storable for at least 1.5 years in the refrigerator. This CF_2_CF_2_-containing organozinc reagent could be easily transmetallated to copper species, which underwent cross-coupling reactions with various aromatic iodides or acyl chlorides to produce a broad range of CF_2_CF_2_-containing organic molecules in good-to-excellent yields. Therefore, the zinc reagent could become a new and practical synthetic tool for producing functional molecules with a CF_2_CF_2_ fragment.

## Introduction

**Figure 1:**
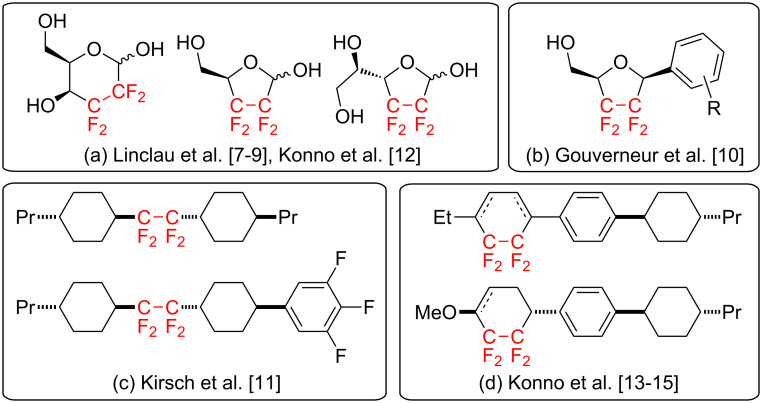
Functional molecules with CF_2_CF_2_-fragment.

Recently, much attention has been paid to organic compounds containing a perfluoroalkylene unit, e.g., –(CF_2_)*_n_*–, in various fields, such as medicine and materials sciences [1–4], because incorporation of multiple fluorine atoms into organic substances causes dramatic alterations in the chemical and physical properties of substances, which may significantly enhance their potential functionality [5–6]. Notably, organofluorine compounds bearing a tetrafluoroethylene (–CF_2_CF_2_–) unit have attracted significant interest as a promising framework for various functional molecules. In the medicinal field, for example, Linclau and co-workers reported the first enantioselective synthesis and the intriguing biological activities of CF_2_CF_2_-containing pyranose and furanose derivatives (Figure 1a) [7–9]. Subsequently, Gouverneur et al. also developed novel CF_2_CF_2_-containing C-nucleosides (Figure 1b) [10]. Meanwhile, in the field of materials sciences, Kirsch et al. revealed that the incorporation of the CF_2_CF_2_ unit between two cyclohexane rings caused significant enhancement in thermal stability in a liquid crystalline phase (Figure 1c) [11]. In addition to the development of a convenient access to CF_2_CF_2_-containing pyranoses [12], our group also showed that tricyclic mesogens with a CF_2_CF_2_-containing carbocycle exhibited large negative dielectric anisotropies (Figure 1d), which would be promising for candidates for vertically aligned (VA)-type display materials [13–17]. Owing to such valuable applications, the development of a much more efficient synthetic protocol for CF_2_CF_2_-containing organic molecules has a high priority in various research areas.

A number of synthetic protocols for CF_2_CF_2_-containing molecules have been developed in the last half-decade. Ogoshi and co-workers reported an efficient synthetic protocol for CF_2_CF_2_-containing compounds via carbocupration or oxycupration of tetrafluoroethylene (TFE) [18–20]. Gouverneur et al. disclosed a Cu(I)-mediated cross-coupling reaction using ArCF_2_CF_2_SiMe_3_ derivatives that produced the corresponding tetrafluoroethylenated compounds [21]. Beier’s group also described a broad range of tetrafluoroethylene compounds generated by the reductive coupling of in situ-formed RCF_2_CF_2_MgCl·LiCl with various electrophiles [22–23]. Our group revealed that commercially available 4-bromo-3,3,4,4-tetrafluorobut-1-ene (**1**) underwent a Cu(0)-mediated cross-coupling reaction with aromatic iodides in DMSO at 160 °C in a sealed-tube apparatus [24] or reductive couplings with various carbonyl compounds [25], leading to good yields of versatile CF_2_CF_2_-containing substances. However, many obstacles to practical synthesis remain, e.g., the use of 1,2-dibromotetrafluoroethane (Halon-2402) with ozone depletion and global warming potentials as a starting material [26], explosive and difficult-to-handle gaseous tetrafluoroethylene [18–20], thermally unstable polyfluoroalkylmetal species [8,27–28], etc. Therefore, the development of a more efficient synthetic protocol featuring easy-handling, simple preparation, and thermal stability is highly desirable.

**Scheme 1:**
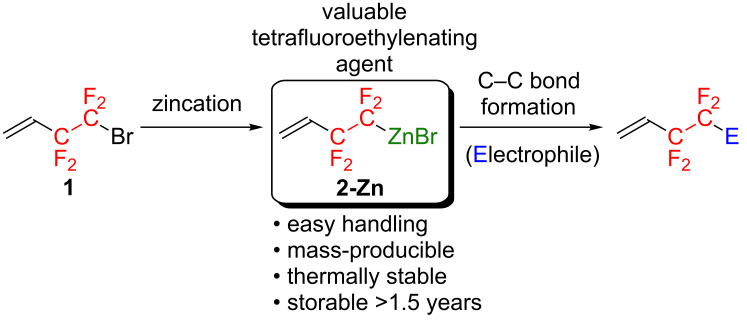
Preparation and synthetic applications of **2-Zn**.

Our strategy focused on the preparation of a thermally stable CF_2_CF_2_-containing metal species for which the “unreactive” form can be easily changed to the “reactive” form through chemical transformation. Out of a variety of organometallics, we selected an organozinc reagent that possesses higher thermal stability than the corresponding organolithium or -magnesium species due to the almost covalent C–Zn bond [29–30]. Moreover, organozinc reagents can be easily transformed to the “reactive” species, through a transmetallation process with a transition metal (e.g., Pd or Cu), which can efficiently construct a new C–C bond [30–32]. Thus, we aimed at the development of an efficient preparation of a thermally stable organozinc reagent, possessing a CF_2_CF_2_ fragment as a thermally stable tetrafluoroethylenating agent, and successive C–C bond formation to produce a wide variety of CF_2_CF_2_-containing molecules, and the results are described in this article (Scheme 1).

## Results and Discussion

We carried out the optimization of the reaction conditions for the preparation of **2-Zn** by direct zinc insertion into the CF_2_–Br bond of commercially available fluorinated substance **1**. The results are summarized in Table 1.

**Table 1:** Optimization of the reaction conditions for the preparation of **2-Zn**.

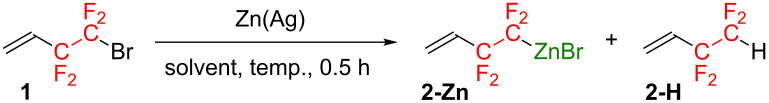						

entry	Zn(Ag) (equiv)	solvent	temp (°C)	yield^a^ (%)		recovery^a^ (% of **1**)
				2-Zn	2-H	

1^b^	2.0	DMF	0	0	0	quant
**2**	**2.0**	**DMF**	**0**	**86**	**9**	**0**
3	1.2	DMF	0	50	12	0
4	2.0	DMF	−30	0	0	quant
5	2.0	DMA	0	58	16	0
6	2.0	DMPU	0	22	28	0
7	2.0	NMP	0	38	20	0
8	2.0	THF	0	0	0	quant
9^c^	2.0	THF	40	75	0	trace

^a^Determined by ^19^F NMR. ^b^With zinc powder pre-activated by dilute HCl aq, instead of Zn(Ag). ^c^Carried out for 4 h.

Initially, we attempted zinc insertion using a typical protocol [33], i.e., treating **1** with 2.0 equiv of zinc powder, pre-activated with dilute HCl solution, in DMF at 0 °C for 0.5 h. Unfortunately, no desired zinc insertion occurred and substrate **1** was quantitatively recovered (Table 1, entry 1). Interestingly, when a zinc–silver couple Zn(Ag) [34] was employed instead of zinc powder, the desired zinc insertion took place very smoothly to form the desired (1,1,2,2-tetrafluorobut-3-en-1-yl)zinc bromide (**2-Zn**) in 86% NMR yield, along with a small amount of a reduction byproduct (**2-H**) (Table 1, entry 2). Reducing the amount of Zn(Ag) slightly retarded the zinc insertion, with only 50% NMR yield (Table 1, entry 3). Lowering the temperature to –30 °C also inhibited the zinc insertion, with quantitative recovery of **1** (Table 1, entry 4). The reaction was also attempted in various solvents, namely *N,N*-dimethylacetamide (DMA), *N,N’*-dimethylpropyleneurea (DMPU), *N*-methylpyrrolidin-2-one (NMP), and THF. In polar solvents (DMA, DMPU, and NMP), the reaction successfully produced the desired **2-Zn**, although the yield was still unsatisfactory (22–58%, Table 1, entries 5–7). In the less polar THF, in contrast, complete recovery of **1** was observed (Table 1, entry 8). Nevertheless, after raising the temperature to 40 °C, directed zinc insertion into the CF_2_–Br bond was achieved in THF, producing **2-Zn** in 75% NMR yield (Table 1, entry 9). Eventually, the optimal conditions for the preparation of **2-Zn** were determined to be that given in entry 2 (Table 1). It is noteworthy that **2-Zn** can be prepared in DMF or THF on a large scale (approx. 40–50 mmol scale) without any problem. More remarkably, the prepared **2-Zn** can be stored in DMF solution (ca. 0.70 M) for at least 1.5 years in the refrigerator [35].

**Figure 2:**
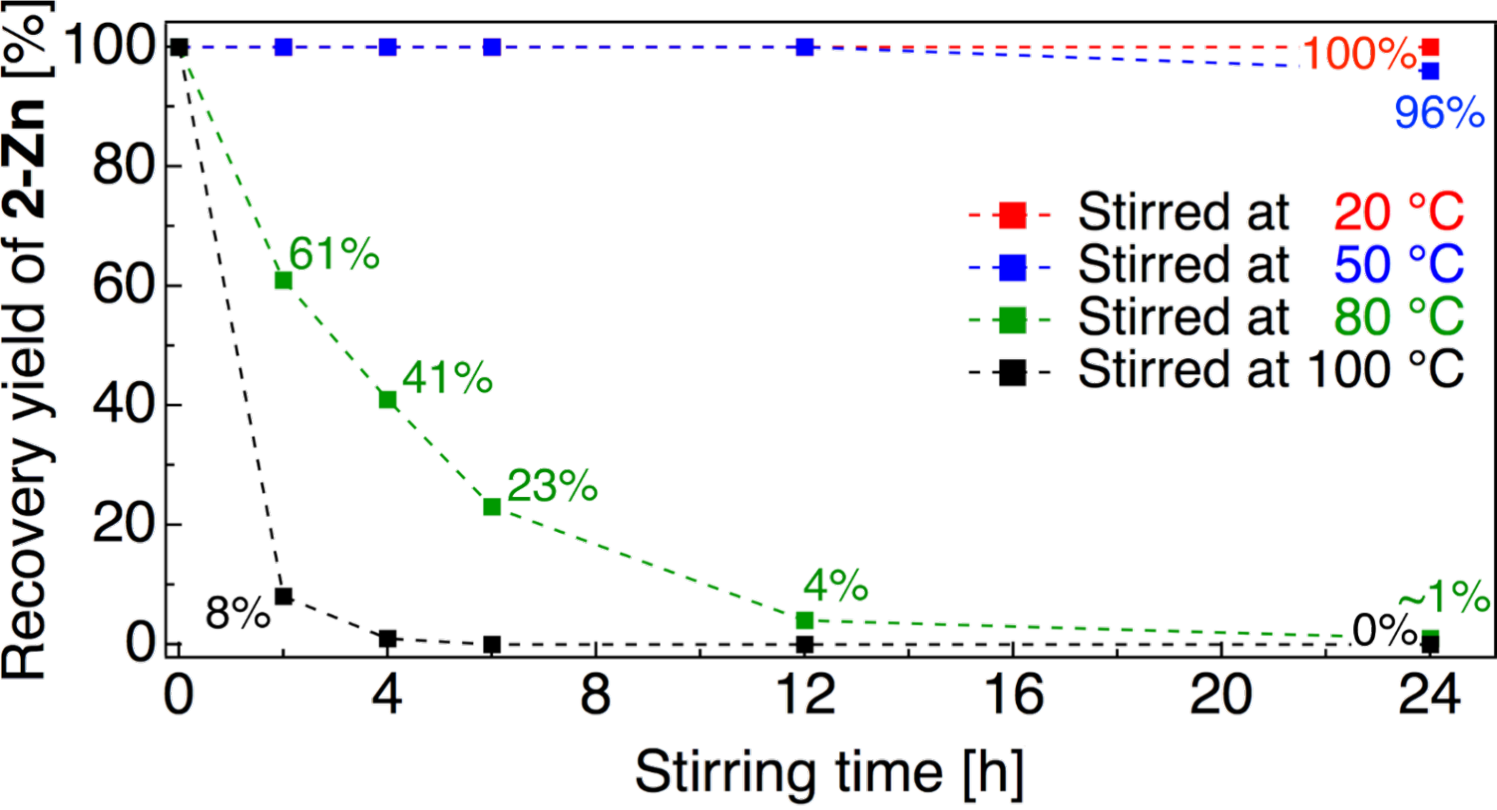
Recovery yield of **2-Zn** in DMF (ca. 0.70 M) after stirring at various temperature conditions.

In order to examine the stability of **2-Zn** in more detail, we quantitatively evaluated the thermal stability of **2-Zn** under various temperature conditions (Figure 2). After a given duration, the recovery yield of **2-Zn** was determined by ^19^F NMR analysis using an internal reference.

Complete recovery of **2-Zn** (0.70 M in DMF) was observed below 50 °C and gradual degradation occurred at 80 °C. It was revealed that 50% decomposition of **2-Zn** was observed after 4 h stirring, and almost all **2-Zn** was decomposed after 12 h stirring (96%). At 100 °C, **2-Zn** was almost completely decomposed after being stirred for 2 h, with a recovery yield of only 8%. According to Beier’s report [22–23], tetrafluoroethylmagnesium species fully decompose within 50 min at −40 °C; CH_2_=CHCF_2_CF_2_ZnBr (**2-Zn**) used in the present study was shown to be much more thermally stable than tetrafluoroethyllithium [12,25] and -magnesium species [22–23].

The synthetic uses of **2-Zn** as a promising tetrafluorinating agent were tested in several reactions. First, we demonstrated a typical C–C bond formation reaction. Treating freshly prepared **2-Zn** (ca. 0.70 M in DMF) with 5.0 equiv of iodobenzene (**3a**) in the presence of 10 mol % of CuI in DMF at 50 °C for 24 h resulted in limited formation (11% yield) of the cross-coupling product **4a** (Table 2, entry 1). The Cu(I)-catalyzed cross-coupling reaction with **3a** was proposed to take place via the following three key reaction steps [36]: (i) transmetallation from **2-Zn** to the corresponding Cu(I) species, (ii) oxidative addition of a C_Ar_–I bond to the Cu(I) atomic center to generate Cu(III) species, and (iii) reductive elimination of the product **4a** along with the regeneration of the Cu(I) salt. When the initial transmetallation from **2-Zn** to the reactive Cu(I) species proceeds much faster than the thermal decomposition of **2-Zn**, the yield of **4a** should be improved. Indeed, in the cross-coupling reaction carried out at 80 °C for 24 h, an enhanced yield of **4a** was observed (Table 2, entry 2), while the reaction in THF did not give any trace of the product (Table 2, entry 3). Catalyst optimization (Table 2, entries 4–6) showed that Cu_2_O led to the highest product yield (46%, Table 2, entry 6) [37]. After further exploring the reaction conditions (Table 2, entries 7–13) including catalyst loading, equivalents of **3a**, additives, and reaction temperature, the best result (64% NMR yield) was observed with 6.0 equiv of **3a** in the presence of 30 mol % of Cu_2_O in DMF at 100 °C for 24 h (Table 2, entry 12) [38].

**Table 2:** Optimization of reaction conditions for Cu(I)-catalyzed cross-coupling reaction of **2-Zn** with iodobenzene (**3a**).

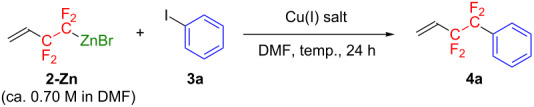				

entry^a^	Cu(I) salt (mol %)	**3a** (equiv)	temp (°C)^b^	yield^c^ (% of **4a**)

1	CuI (10)	5.0	50	11
2	CuI (10)	5.0	80	37
3^d^	CuI (10)	5.0	80	0
4	CuBr (10)	5.0	80	37
5	CuCl (10)	5.0	80	34
6	Cu_2_O (10)	5.0	80	46
7	Cu_2_O (30)	5.0	80	53
8	Cu_2_O (50)	5.0	80	54
9^e^	Cu_2_O (30)	5.0	80	50
10^f^	Cu_2_O (30)	5.0	80	35
11	Cu_2_O (30)	6.0	80	59
**12**	**Cu_2_O (30)**	**6.0**	**100**	**64 (29)**^g^
13	Cu_2_O (30)	6.0	120	57

^a^Carried out using ca. 0.6 mmol of **2-Zn** in DMF solution (ca. 0.70 M). ^b^Bath temperature. ^c^Determined by ^19^F NMR. Values in parentheses are isolated yields. ^d^The reaction was carried out using **2-Zn** in THF instead of **2-Zn** in DMF. ^e^*N,N,N’,N’*-Tetramethylethylenediamine (TMEDA) was used as an additive. ^f^1,10-Phenanthroline (phen) was used as an additive. ^g^The low isolated yield was due to the low boiling point and/or the high volatility of the product.

**Figure 3:**
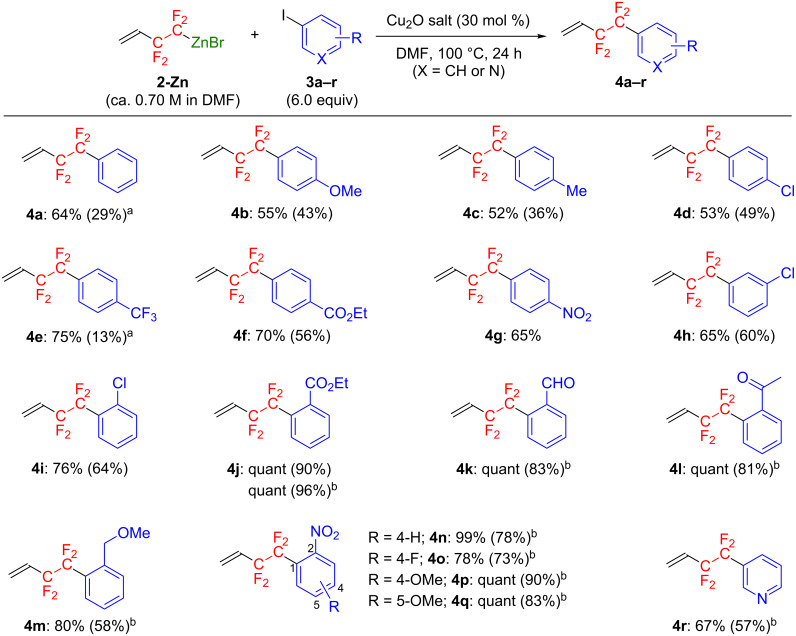
Copper(I)-catalyzed cross-coupling reaction of **2-Zn** with various iodoarene derivatives. NMR (isolated) yields are indicated. ^a^Low yields are due to the low boiling point and/or the high volatility of the products. ^b^Reaction was carried out using 1.0 equiv of **3** and 2.0 equiv of **2-Zn** in the presence of 60 mol % of Cu_2_O.

Using the optimal conditions for the reaction in entry 12, Table 2, various iodoarenes or -heteroarenes (**3b**–**r**) could be converted into the corresponding CF_2_CF_2_-substituted aromatic compounds **4b**–**r** (Figure 3).

Aromatic iodides with an electron-donating group, such as OMe (**3b**) and Me (**3c**), at the *p*ara position on the benzene ring were successfully coupled with **2-Zn** under the optimized conditions to form **4b** and **4c** in 43% and 36% isolated yields, respectively. *p*-Chloro- (**3d**) or *p*-trifluoromethyl-substituted iodobenzene (**3e**) could also participate in the coupling reaction, leading to the corresponding products **4d** and **4e** in 53% and 75% NMR (49% and 13% isolated) yields, respectively. Notably, iodoarenes with an ethoxycarbonyl (**3f**) or a nitro group (**3g**) at the *para*-position were successfully converted to the corresponding products **4f** and **4g** with good yields. Differences in the position of the substituents on the benzene ring had no effect on the coupling reaction; *m*-chloro- (**3h**) and *o*-chloroiodobenzene (**3i**) were transformed into their corresponding products **4h** and **4i** in 60% and 64% yields, respectively. Interestingly, the reaction using ethyl *o*-iodobenzoate (**3j**) quite efficiently proceeded to give the coupling product **4j** almost quantitatively. Comparing the result from the reaction with the *para*-substituted analog **3f**, the ester functionality at the *ortho*-position seems to significantly facilitate the formation of the coupling product. According to the previous reports by Jiang and co-workers [36], the oxygen atom in the ethoxycarbonyl group substituted at the *ortho-*position coordinates with the copper center to stabilize the Cu(III) intermediate, which facilitates the subsequent reductive elimination to form the desired coupling product. This acceleration effect of the ester functional group at the *ortho*-position led to a reduction in the chemical substances used. That is to say, the product **4j** was obtained in a quantitative manner even when the reaction of **2-Zn** was carried out with only a half equivalent of **3j**. The same effect was also noted for iodoarenes having a labile functional group, such as formyl (**3k**), acetyl (**3l**), methoxymethyl (**3m**), or a nitro group (**3n**–**q**) at the *ortho*-position, giving rise to the corresponding products **4k**–**q** with excellent efficiency. These results strongly suggest that **2-Zn** can be successfully employed for the coupling reaction with an electrophile bearing a reactive functional group. Lastly, this synthetic protocol could be applied to prepare the CF_2_CF_2_-substituted heteroaromatic compound **4r** from 3-iodopyridine (**3r**), demonstrating a promising pathway for constructing CF_2_CF_2_-containing heterocyclic compounds.

**Scheme 2:**
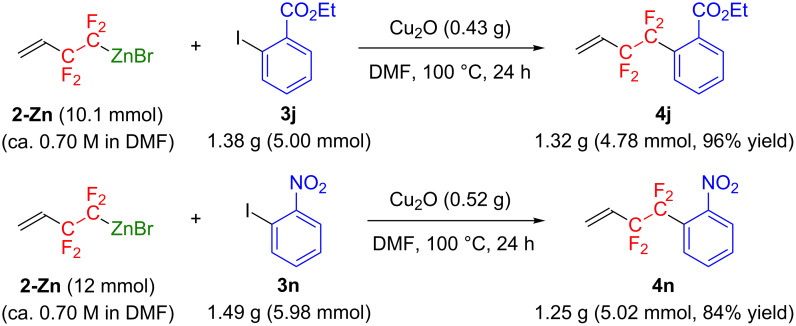
Multigram-scale cross-coupling of **2-Zn** with iodoarenes.

We also demonstrated the multigram preparation of CF_2_CF_2_-containing arenes through the present cross-coupling reaction, as shown in Scheme 2. Thus, treatment of 1.38 g (5.00 mmol) of ethyl *o*-iodobenzoate (**3j**) with 10.1 mmol of **2-Zn** in the presence of 3 mmol of Cu_2_O in DMF at 100 °C for 24 h gave 1.32 g (4.78 mmol) of the corresponding coupling product **4j** (96% yield). Similarly, the reaction of **2-Zn** with 1.49 g of *o*-iodonitrobenzene (**3n**) afforded 1.25 g (5.02 mmol) of the adduct **4n** (84% yield). This achievement may lead to a significant contribution as a first scalable CF_2_CF_2_ fragment installation method.

**Scheme 3:**
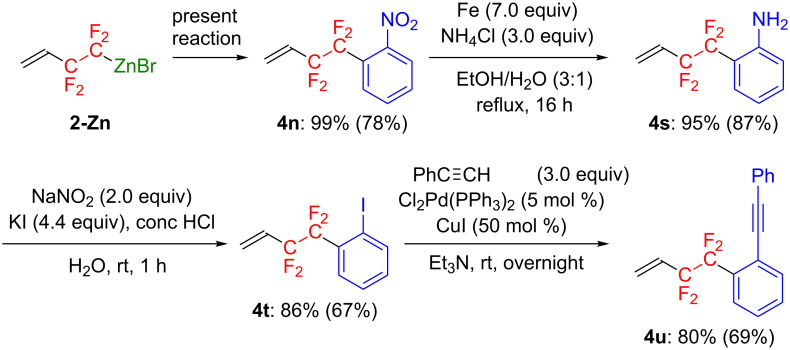
Synthesis of a CF_2_CF_2_ group containing tolane derivative.

To be further convinced of the importance of the present reaction, we carried out an additional transformation of the coupling adduct **4n** to a CF_2_CF_2_-containing π-conjugates molecule, a fluorinated tolane derivative, applicable to promising functional materials, such as light-emitting and liquid-crystalline materials (Scheme 3) [39–44].

Thus, CF_2_CF_2_ group containing **4n** with a nitro group at the *ortho*-position of the aromatic ring was effectively prepared from **2-Zn** with the present protocol. **4n** was smoothly converted to the corresponding aniline derivative **4s** in 87% isolated yield, after exposure to a reductive environment using Fe powder and NH_4_Cl. Subsequent Sandmeyer reaction of **4s** took place very nicely to afford the corresponding CF_2_CF_2_-substituted iodobenzene derivative **4t** in 67% isolated yield. Then, **4t** underwent Pd(0)-catalyzed Sonogashira cross-coupling reaction with phenylacetylene, producing the corresponding tolane derivative **4u** with a CF_2_CF_2_ fragment in good yield (30% overall yield from **2-Zn**). Consequently, **2-Zn** is found to be a powerful tetrafluoroethylenating agent for producing a broad range of organic molecules with a CF_2_CF_2_ unit.

Next, the Cu(I)-catalyzed cross-coupling reaction of **2-Zn** with benzoyl chloride (**5a**) as a coupling partner was investigated (Table 3).

**Table 3:** Optimization of reaction conditions for Cu(I)-catalyzed cross-coupling reaction of **2-Zn** with benzoyl chloride (**5a**).

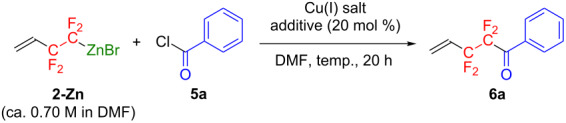					

entry	Cu(I) salt (mol %)	**5a** (equiv)	additive	temp^a^ (°C)	yield^b^ (% of **4a**)

1	CuI (30)	2.4	–	100	67
2	CuBr (30)	2.4	–	100	51
3	CuCN (30)	2.4	–	100	46
4	Cu_2_O (30)	2.4	–	100	81
5	Cu_2_O (10)	2.4	TMEDA	100	nd^c^
6	Cu_2_O (30)	2.4	phen	100	69
7	Cu_2_O (50)	2.4	bpy	100	quant
**8**	**Cu_2_O (30)**	**2.4**	**bpy**	**rt**	**quant (92)**
9	Cu_2_O (30)	2.0	bpy	rt	83

^a^Bath temperature. ^b^Determined by ^19^F NMR. Values in parentheses are isolated yield. ^c^Not detected.

Treating **2-Zn** (ca. 0.70 M DMF solution) with 2.4 equiv of **5a** in the presence of CuI (30 mol %) in DMF at 100 °C for 20 h enabled the formation of the corresponding cross-coupling reaction product **6a** in 67% yield (Table 3, entry 1). Optimization of the Cu(I) catalyst for the benzoylation reaction (Table 3, entries 2–4) revealed that Cu_2_O was the most efficient for producing **6a** (81% by NMR, Table 3, entry 4). To further improve the reaction efficiency, three different additives (TMEDA, phen, and 2,2’-bipyridyl (bpy)) were tested. The first two additives were found to be ineffective for the present reaction (Table 3, entries 5 and 6), whereas bpy led to the quantitative formation of **6a** (Table 3, entry 7). The facilitative effect of bpy as an additive made it possible to convert **5a** to **6a** even at ambient temperature (Table 3, entry 8). Additionally, to utilize the present reaction in an environmentally benign protocol, we conducted the reaction with a decreased amount of **5a**. Unfortunately, this slightly lowered the yield of **6a** (Table 3, entry 9).

**Figure 4:**
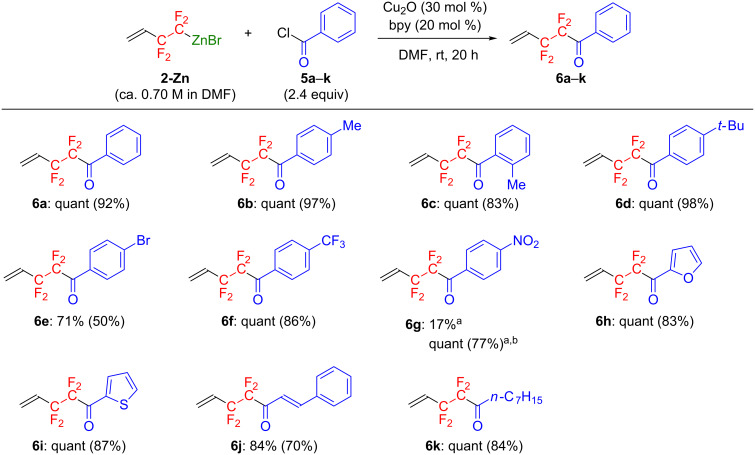
Copper(I)-catalyzed cross-coupling reaction of **2-Zn** with various acid chlorides. NMR yields (isolated yields) are indicated. ^a^Carried out at 100 °C. ^b^With 4.0 equiv of **5g**.

With the optimized conditions (Table 3, entry 8), various kinds of acid chlorides **5b**–**k** could be converted to the corresponding CF_2_CF_2_-substituted products **6b**–**k** (Figure 4).

Benzoyl chloride derivatives with an electron-donating (**5b**–**d**) and an electron-withdrawing group (**5e**,**f**) gave rise to the corresponding coupling products **6b**–**f** in 50–98% isolated yields. However, the reaction using nitro-substituted substrate **5g** was quite slow under the same conditions. Slight modification of the reaction conditions, namely increasing the amount of **5g** and raising the reaction temperature, significantly improved the yield of **6g**. Acid chlorides with a heteroaromatic moiety, e.g., **5h** and **5i**, could also readily participate in the coupling reaction, leading to **6h** and **6i** in 83% and 87% isolated yields, respectively. Additionally, cinnamoyl chloride (**5j**) and *n*-octanoyl chloride (**5k**) were also suitable electrophiles to form the respective products **6j** and **6k** in high-to-excellent yields.

## Conclusion

We developed a novel and practical tetrafluoroethylenating agent, viz. CH_2_=CHCF_2_CF_2_ZnBr (**2-Zn**), which can be prepared in large-scale and stored for at least 1.5 years in the refrigerator without decomposition. **2-Zn** could be successfully transformed into a broad range of CF_2_CF_2_-containing molecules with good-to-excellent efficiency. Considering that our previous study found that the vinyl moiety in the coupling product could be a useful molecular building block [14,45], **2-Zn** presented here should be a suitable and valuable tetrafluoroethylenating agent for preparing various CF_2_CF_2_-containing molecules, thereby providing a powerful and practical synthetic tool in organofluorine chemistry.

## Supporting Information

File 1 Experimental procedures, characterization data (^1^H, ^13^C, ^19^F NMR, IR and HRMS), copies of ^1^H, ^13^C and ^19^F NMR spectra.
