# Supplementary material for: Practical tetrafluoroethylene fragment installation through a coupling reaction of (1,1,2,2-tetrafluorobut-3-en-1-yl)zinc bromide with various electrophiles
Source: Beilstein J Org Chem. 2018 Sep 11;14:2375–83. doi: 10.3762/bjoc.14.213 (PMC6142754; doi:10.3762/bjoc.14.213)

Supporting Information

for

Practical tetrafluoroethylene fragment installation through a coupling reaction of (1,1,2,2-tetrafluorobut-3-en-1-yl)zinc bromide with various electrophiles

Ken Tamamoto, Shigeyuki Yamada and Tsutomu Konno*

Address: Faculty of Molecular Chemistry and Engineering, Kyoto Institute of Technology, Matsugasaki, Sakyo-ku, Kyoto 606-8585, Japan

Email: Tsutomu Konno - konno@kit.ac.jp

*Corresponding author

Experimental procedures, characterization data (^{1}H, ^{13}C, ^{19}F NMR, IR and HRMS), copies of ^{1}H, ^{13}C and ^{19}F NMR spectra

Table of Contents

1.	Experimental section	S5
	General experimental	S5
	Materials	S5
	Preparation of Zn–Ag couple	S6
	Preparation of CH_2=$CHCF_2CF_2ZnBr$ in DMF (**2-Zn**)	S6
	(1,1,2,2-Tetrafluorobut-3-en-1-yl)zinc bromide in DMF (**2-Zn**)	S7
	Typical procedure for Cu(I)-catalyzed cross-coupling reaction of organozinc reagent **2-Zn** with iodobenzene (**3a**)	S7
	3,3,4,4-Tetrafluoro-4-phenylbut-1-ene (**4a**)	S7
	3,3,4,4-Tetrafluoro-4-(4-methoxyphenyl)but-1-ene (**4b**)	S8
	3,3,4,4-Tetrafluoro-4-(4-methylphenyl)but-1-ene (**4c**)	S8
	4-(4-Chlorophenyl)-3,3,4,4-tetrafluorobut-1-ene (**4d**)	S9
	3,3,4,4-Tetrafluoro-4-(4-trifluoromethylphenyl)but-1-ene (**4e**)	S10
	Ethyl 4-(1,1,2,2-tetrafluorobut-3-en-1yl)benzoate (**4f**)	S10
	3,3,4,4-Tetrafluoro-4-(4-nitrophenyl)but-1-ene (**4g**)	S11
	4-(3-Chlorophenyl)-3,3,4,4-tetrafluorobut-1-ene (**4h**)	S11
	4-(2-Chlorophenyl)-3,3,4,4-tetrafluorobut-1-ene (**4i**)	S12
	Typical procedure for improved Cu(I)-catalyzed cross-coupling reaction of organozinc reagent **2-Zn** with ethyl 2-iodobenzoate (**3j**)	S13
	Ethyl 2-(1,1,2,2-tetrafluorobut-3-en-1-yl)benzoate (**4j**)	S13
	3,3,4,4-Tetrafluoro-4-(2-formylphenyl)but-1-ene (**4k**)	S14
	3,3,4,4-Tetrafluoro-4-(2-acetylphenyl)but-1-ene (**4l**)	S14
	3,3,4,4-Tetrafluoro-4-(2-methoxymethylphenyl)but-1-ene (**4m**)	S15
	3,3,4,4-Tetrafluoro-4-(2-nitrophenyl)but-1-ene (**4n**)	S16
	3,3,4,4-Tetrafluoro-3-(4-fluoro-2-nitrophenyl)but-1-ene (**4o**)	S16
	3,3,4,4-Tetrafluoro-4-(4-methoxy-2-nitrophenyl)but-1-ene (**4p**)	S17
	3,3,4,4-Tetrafluoro-4-(5-methoxy-2-nitrophenyl)but-1-ene (**4q**)	S18
	3-(1,1,2,2-Tetrafluorout-3-en-1-yl)pyridine (**4r**)	S18
	Typical procedure for the amination of **4n**	S19
	2-(1,1,2,2-Tetrafluorobut-3-en-1-yl)aniline (**4s**)	S19
	Typical procedure for the Sandmeyer reaction of **4s**	S20
	3,3,4,4-Tetrafluoro-4-(2-iodophenyl)but-1-ene (**4t**)	S20
	Typical procedure for the Sonogashira cross-coupling reaction of 4s with phenylacetylene	S21
	1-(2-Phenylethynyl)-2-(2,2,3,3-tetrafluorobut-3-en-1-yl)benzene (**4u**)	S21
	Typical procedure for Cu(I)-catalyzed cross-coupling reaction of organozinc reagent **2-Zn** with benzoyl chloride (**5a**)	S22

2,2,3,3-Tetrafluoro-1-phenylpent-4-en-1-one (**6a**) S22
2,2,3,3-Tetrafluoro-1-(4-methylphenyl)pent-4-en-1-one (**6b**) S23
2,2,3,3-Tetrafluoro-1-(2-methylphenyl)pent-4-en-1-one (**6c**) S24
1-(*tert*-Butylphenyl)-2,2,3,3-tetrafluoropent-4-en-1-one (**6d**) S24
1-(4-Bromophenyl)-2,2,3,3-tetrafluoropent-4-en-1-one (**6e**) S25
2,2,3,3-Tetrafluoro-1-(4-trifluoromethylphenyl)pent-4-en-1-one (**6f**) S25
2,2,3,3-Tetrafluoro-1-(furan-2-yl)pent-4-en-1-one (**6h**) S26
2,2,3,3-Tetrafluoro-1-(thiophen-2-yl)pent-4-en-1-one (**6i**) S27
4,4,5,5-Tetrafluoro-1-phenylhept-1,6-dien-3-one (**6j**) S27
3,3,4,4-Tetrafluorododec-1-en-5-one (**6k**) S28
Typical procedure for improved Cu(I)-catalyzed cross-coupling reaction of organozinc reagent 2-Zn with 4-nitrobenzoyl chloride (**5g**) S28
2,2,3,3-Tetrafluoro1-(4-nitrophenyl)pent-4-en-1-one (**6g**) S29

2. Additional scheme and figure. S30
Scheme S1 S30
Figure S1 S30

3 Copies of ^{1}H, ^{13}C, and ^{19}F NMR spectra for new compounds S31
^{1}H, ^{13}C, and ^{19}F NMR Spectra of 3,3,4,4-tetrafluoro-4-(4-trifluoromethylphenyl) but-1-ene (**4e**) S31–S32
^{1}H, ^{13}C, and ^{19}F NMR Spectra of 4-(3-chlorophenyl)-3,3,4,4-tetrafluorobut-1-ene (**4h**) S33–S34
^{1}H, ^{13}C, and ^{19}F NMR Spectra of 4-(2-chlorophenyl)-3,3,4,4-tetrafluorobut-1-ene (**4i**) S35–S36
^{1}H, ^{13}C, and ^{19}F NMR Spectra of ethyl 2-(1,1,2,2-tetrafluorobut-3-en-1-yl)benzoate (**4j**) S37–S38
^{1}H, ^{13}C, and ^{19}F NMR Spectra of 3,3,4,4-tetrafluoro-4-(2-formylphenyl)but-1-ene (**4k**) S39–S40
^{1}H, ^{13}C, and ^{19}F NMR Spectra of 3,3,4,4-tetrafluoro-4-(2-methoxymethylphenyl) but-1-ene (**4m**) S41–S42
^{1}H, ^{13}C, and ^{19}F NMR Spectra of 3,3,4,4-tetrafluoro-4-(2-nitrophenyl)but-1-ene (**4n**) S43–S44
^{1}H, ^{13}C, and ^{19}F NMR Spectra of 4-(4-fluoro-2-nitrophenyl)-3,3,4,4-tetrafluoro but-1-ene (**4o**) S45–S46
^{1}H, ^{13}C, and ^{19}F NMR Spectra of 3,3,4,4-tetrafluoro-4-(4-methoxy-2-nitrophenyl) but-1-ene (**4p**) S47–S48
^{1}H, ^{13}C, and ^{19}F NMR Spectra of 3,3,4,4-tetrafluoro-4-(5-methoxy-2-nitrophenyl) but-1-ene (**4q**) S49–S50
^{1}H, ^{13}C, and ^{19}F NMR Spectra of 2-(1,1,2,2-tetafluorobut-3-en-1-yl)aniline (**4s**) S51–S52
^{1}H, ^{13}C, and ^{19}F NMR Spectra of 3,3,4,4-tetrafluoro-4-(2-iodophenyl)but-1-ene (**4t**) S53–S54
^{1}H, ^{13}C, and ^{19}F NMR Spectra of 1-(2-phenylethynyl)-2-(2,2,3,3-tetrafluoro but-3-en-1-yl)benzene (**4u**) S55–S56
^{1}H, ^{13}C, and ^{19}F NMR Spectra of 2,2,3,3-tetrafluoro-1-phenylpent-4-en-1-one (**6a**) S57–S58
^{1}H, ^{13}C, and ^{19}F NMR Spectra of 2,2,3,3-tetrafluoro-1-(4-methylphenyl)

pent-4-en-1-one (**6b**) S59–S60
^{1}H, ^{13}C, and ^{19}F NMR Spectra of 2,2,3,3-tetrafluoro-1-(2-methylphenyl)
pent-4-en-1-one (**6c**) S61–S62
^{1}H, ^{13}C, and ^{19}F NMR Spectra of 1-(4-*tert*-butylphenyl)-2,2,3,3-tetrafluoropent-
4-en-1-one (**6d**) S63–S64
^{1}H, ^{13}C, and ^{19}F NMR Spectra of 1-(4-bromophenyl)-2,2,3,3-tetrafluoropent-
4-en-1-one (**6e**) S65–S66
^{1}H, ^{13}C, and ^{19}F NMR Spectra of 2,2,3,3-tetrafluoro-1-(4-trifluoromethylphenyl)
pent-4-en-1-one (**6f**) S67–S68
^{1}H, ^{13}C, and ^{19}F NMR Spectra of 2,2,3,3-tetrafluoro-1-(4-nitrophenyl)
pent-4-en-1-one (**6g**) S69–S70
^{1}H, ^{13}C, and ^{19}F NMR Spectra of 2,2,3,3-tetrafluoro-1-(furan-2-yl) S71–S72
pent-4-en-1-one (**6h**)
^{1}H, ^{13}C, and ^{19}F NMR Spectra of 2,2,3,3-tetrafluoro-1-(thiophen-2-yl) S73–S74
pent-4-en-1-one (**6i**)
^{1}H, ^{13}C, and ^{19}F NMR Spectra of 4,4,5,5-tetrafluoro-1-phenylhept-1,6-dien-3-one (**6j**) S75–S76
^{1}H, ^{13}C, and ^{19}F NMR Spectra of 3,3,4,4-tetrafluorododec-1-en-5-one (**6k**) S77–S78

1. Experimental section

General experimental

Infrared spectra (IR) were determined in a liquid film on a NaCl plate with a JASCO FT/IR-4100 type A spectrometer and all spectra are reported in wavenumber (cm^{-1}). ^{1}H and ^{13}C NMR spectra were measured with a Bruker AVANCE III 400 NMR spectrometer (^{1}H: 400 MHz and ^{13}C: 100 MHz) in a chloroform-*d* ($CDCl_3$) solution and the chemical shifts are reported in parts per million (ppm) using the residual proton or carbon in the NMR solvent. A Bruker AVANCE III 400 NMR spectrometer was used for determining the yield of the products with hexafluorobenzene (C_6F_6) or benzotrifluoride ($CF_3C_6H_5$). ^{19}F NMR (376.05 MHz) spectra were measured with a Bruker AVANCE III 400 NMR spectrometer in a $CDCl_3$ solution with trichlorofluoromethane ($CFCl_3$, δ_F = 0 ppm) as an internal standard. High-resolution mass spectra (HRMS) were taken on a JEOL JMS-700MS spectrometer by fast atom bombardment (FAB) method.

All reactions were carried out using dried glassware with a magnetic stirrer bar under an atmosphere of argon and routinely monitored by ^{19}F NMR spectroscopy or thin-layer chromatography (TLC).

Materials

All chemicals were of reagent grade and, if necessary, were purified in the usual manner prior to use.

Column chromatography was carried out on silica gel (Wako gel® C-200) and TLC was performed on silica gel TLC plates (Merck, Silica gel 60 F_{254}).

Preparation of Zn–Ag couple

To a stirred solution of silver acetate (10 mg, 0.060 mmol) in glacial acetic acid (20 mL) at reflux temperature was added zinc powder (10 g, 153 mmol) in one portion, and the whole was stirred at that temperature for additional 30 seconds. After refluxing for 30 seconds, the flask was cooled by dipping in an ice-bath. The Zn–Ag couple formed was separated by vacuum filtration in the open-air, which was washed with Et_2O several times, followed by drying under reduced pressure. The Zn–Ag couple separated was stored at room temperature under Ar atmosphere. See Figure S1

Preparation of $CH_2=CHCF_2CF_2ZnBr$ in DMF (2-Zn)

In a two-necked round-bottomed flask, equipped with a teflon-coated stirrer bar, was placed Zn–Ag couple (0.66 g, 10 mmol) in DMF (5.0 mL). To the suspension was slowly dropwise added 4-bromo-3,3,4,4-tetrafluorobut-1-ene (**1**, 1.0 g, 5.0 mmol) at 0 ºC and the whole was stirred at that temperature for 30 min. The resultant was subjected to a short pad of celite and washed by Et_2O three times. The filtrate was concentrated in vacuo to give the corresponding organozinc reagents **2-Zn** in 86% NMR yield as a 0.70 M DMF solution.

(1,1,2,2-Tetrafluorobut-3-en-1-yl)zinc bromide in DMF (2-Zn)

Yield: 86% (determined by ^{19}F NMR); ^{19}F NMR ($CDCl_3$): δ −112.10 (brs, 2F, C*F*$_2$CH=), −120.23 (brs, 2F, C*F*$_2$Zn).

Typical procedure for Cu(I)-catalyzed cross-coupling reaction of organozinc reagent 2-Zn with iodobenzene (3a)

In a two-necked round-bottomed flask, equipped with a teflon-coated stirrer bar, were added iodobenzene (**3a**, 0.40 mL, 3.6 mmol) and Cu_2O (0.027 g, 0.18 mmol), and a DMF solution of **2-Zn** (0.60 M, 1.0 mL, 0.6 mmol). The whole was heated up at 100 ºC and stirred at that temperature for 24 h. After cooling to room temperature, the resultant was filtered through a short pad of silica gel using hexane as an eluent. The filtrate was concentrated in vacuo to give the crude materials, which was purified by silica gel column chromatography using hexanes as an eluent, leading to the corresponding compound **4a** (0.036 g, 0.17 mmol) in 29% isolated yield as a colorless oil.

3,3,4,4-Tetrafluoro-4-phenylbut-1-ene (4a) [S1]

Known compound; Yield: 29% (0.036 g, 0.17 mmol); Colorless oil (hexanes, R_f = 0.57); ^{1}H NMR ($CDCl_3$): δ 5.69 (d, *J* = 11.6 Hz, 1H, *trans*-C*H*$_2$=CHCF$_2$), 5.83 (dt, *J* = 17.2, 1.6 Hz, 1H, *cis*-C*H*$_2$=CHCF$_2$), 6.02 (ddt, *J* = 17.2, 11.6, 11.5 Hz, 1H, CF$_2$C*H*=), 7.44–7.58 (m, 5H, Ar*H*); ^{13}C NMR ($CDCl_3$): δ 115.1 (tt, *J* = 248.8, 37.2 Hz, *C*F$_2$), 116.4 (tt, *J* = 252.1, 35.5 Hz, *C*F$_2$), 124.1 (t, *J* = 9.9

Hz, CH_2=), 126.7 (t, J = 24.8 Hz, CF_2CH=), 126.9 (td, J = 6.6, 1.7 Hz, Ar), 127.0–127.7 (m, Ar), 128.2 (Ar), 131.0 (t, J = 1.7 Hz, Ar); ^{19}F NMR ($CDCl_3$): δ −112.56 (s, 2F, CF_2Ar), −114.95 (d, J = 11.46 Hz, 2F, CF_2CH=); IR (neat): ν 3071, 1653, 1424, 1286, 1243, 1130, 1070, 949 cm^{-1}; HRMS (FAB) calcd for $[M]^+$ $C_{10}H_8F_4$: 204.0562, found 204.0558.

3,3,4,4-Tetrafluoro-4-(4-methoxyphenyl)but-1-ene (4b) [S1]

Known compound; Yield: 43% (0.065 g, 0.28 mmol); Colorless oil (hexanes, R_f = 0.20); ^{1}H NMR ($CDCl_3$): δ 3.85 (s, 3H, CH_3), 5.67 (d, J = 11.3 Hz, 1H, *trans*-CH_2=$CHCF_2$), 5.81 (dt, J = 17.2, 2.0 Hz, 1H, *cis*-CH_2=$CHCF_2$), 6.00 (ddt, J = 17.2, 11.4, 11.3 Hz, 1H, CF_2CH=), 6.95 (d, J = 8.8 Hz, 2H, Ar*H*), 7.48 (d, J = 8.8 Hz, 2H, Ar*H*); ^{13}C NMR ($CDCl_3$): δ 55.7 (CH_3), 114.0 (Ar), 115.5 (tt, J = 249.0, 38.2 Hz, CF_2), 116.9 (tt, J = 251.6, 35.1 Hz, CF_2), 123.2 (t, J = 25.4 Hz, Ar), 124.3 (t, J = 9.2 Hz, CH_2=), 127.3 (t, J = 24.9 Hz, CF_2CH=), 128.7 (t, J = 6.3 Hz, Ar), 161.9 (Ar); ^{19}F NMR ($CDCl_3$): δ −111.52 (s, 2F, CF_2Ar), −115.01 (d, J = 11.4 Hz, 2F, CF_2CH=); IR (neat): ν 2938, 1519, 1420, 1260, 1181, 1095, 1074, 982, 832 cm^{-1}; HRMS (FAB) calcd for $[M]^+$ $C_{11}H_{10}F_4O$: 234.0668, found 234.0672.

3,3,4,4-Tetrafluoro-4-(4-methylphenyl)but-1-ene (4c) [S1]

Known compound; Yield: 36% (0.046 g, 0.21 mmol); Colorless oil (hexanes, R_f = 0.37); ^{1}H NMR ($CDCl_3$): δ 2.41 (s, 3H, CH_3), 5.68 (d, J = 11.2 Hz, 1H, *trans*-CH_2=$CHCF_2$), 5.82 (dt, J = 17.5, 2.0

Hz, 1H, *cis*- C*H*$_2$=CHCF$_2$), 6.01 (ddt, *J* = 17.5, 11.4, 11.2 Hz, 1H, CF$_2$C*H*=), 7.26 (d, *J* = 8.4 Hz, 2H, Ar*H*), 7.44 (d, *J* = 8.4 Hz, 2H Ar*H*); ^{13}C NMR (CDCl$_3$): δ 21.3 (*C*H$_3$), 115.1 (tt, *J* = 248.4, 37.6 Hz, *C*F$_2$), 116.6 (tt, *J* = 251.3, 35.1 Hz, *C*F$_2$), 124.0 (t, *J* = 9.5 Hz, *C*H$_2$=), 126.8 (t, *J* = 24.8 Hz, CF$_2$C*H*=), 126.6–127.1 (m, Ar), 127.9 (t, *J* = 24.8 Hz, Ar), 128.9 (Ar), 141.2 (t, *J* = 1.7 Hz, Ar); ^{19}F NMR (CDCl$_3$): δ –112.24 (s, 2F, C*F*$_2$Ar), –115.04 (d, *J* = 11.4 Hz, 2F, C*F*$_2$CH=); IR (neat): ν 3043, 2927, 1617, 1420, 1288, 1243, 1121, 1074, 982, 818 cm^{-1}; HRMS (FAB) calcd for $[M]^+$ $C_{11}H_{10}F_4$: 218.0719, found 218.0726.

4-(4-Chlorophenyl)-3,3,4,4-tetrafluorobut-1-ene (4d) [S1]

Known compound; Yield: 49% (0.072 g, 0.30 mmol); Colorless oil (hexanes, R$_f$ = 0.50); ^{1}H NMR (CDCl$_3$): δ 5.71 (d, *J* = 11.2 Hz, 1H, *trans*-C*H*$_2$=CHCF$_2$), 5.84 (dt, *J* = 17.2, 2.0 Hz, 1H, *cis*-C*H*$_2$=CHCF$_2$), 6.01 (ddt, *J* = 17.2, 11.2, 11.3 Hz, 1H, CF$_2$C*H*=), 7.44 (d, *J* = 8.8 Hz, 2H, Ar*H*), 7.50 (d, *J* = 8.8 Hz, Ar*H*); ^{13}C NMR (CDCl$_3$): δ 115.1 (tt, *J* = 249.1, 37.6 Hz, *C*F$_2$), 116.2 (tt, *J* = 252.1, 35.6 Hz, *C*F$_2$), 124.6 (t, *J* = 9.2 Hz, *C*H$_2$=), 126.5 (t, *J* = 24.7 Hz, CF$_2$*C*H=), 128.5 (t, *J* = 6.3 Hz, Ar), 128.8 (Ar), 129.4 (t, *J* = 25.3 Hz, Ar), 137.6 (t, *J* = 1.7 Hz, Ar); ^{19}F NMR (CDCl$_3$): δ –112.44 (s, 2F, C*F*$_2$Ar), –114.80 (d, *J* = 11.3 Hz, 2F, C*F*$_2$CH=); IR (neat): ν 2925, 2857, 1604, 1491, 1218, 1135, 1095, 1074, 957, 826 cm^{-1}; HRMS (FAB) calcd for $[M]^+$ $C_{10}H_7ClF_4$: 238.0172, found 238.0170.

3,3,4,4-Tetrafluoro-4-(4-trifluoromethylphenyl)but-1-ene (4e)

Yield: 13% (0.036 g, 0.13 mmol); Colorless oil (hexanes, R_f = 0.57); ^{1}H NMR ($CDCl_3$): δ 5.74 (d, *J* = 11.1 Hz, 1H, *trans*-CH_2=CHCF_2), 5.86 (dt, *J* = 17.3, 2.1 Hz, 1H, *cis*-CH_2=CHCF_2), 6.03 (ddt, *J* = 17.3, 11.2, 11.1 Hz, 1H, CF_2C*H*=), 7.70 (d, *J* = 8.6 Hz, 2H, Ar*H*), 7.74 (d, *J* = 8.6 Hz, 2H, Ar*H*); ^{13}C NMR ($CDCl_3$): δ 115.1 (tt, *J* = 248.9, 36.9 Hz, *C*F_2), 116.0 (tt, *J* = 252.4, 36.1 Hz, *C*F_2), 123.7 (q, *J* = 272.6 Hz, *C*F_3), 124.9 (t, *J* = 9.3 Hz, *C*H_2=), 125.5 (q, *J* = 3.7 Hz, Ar), 126.3 (t, *J* = 24.6 Hz, CF_2*C*H=), 127.7 (t, *J* = 6.5 Hz, Ar), 133.3 (q, *J* = 33.0 Hz, Ar), 134.5 (t, *J* = 25.0 Hz, Ar); ^{19}F NMR ($CDCl_3$): δ −63.57 (s, 3F, CF_3), −112.81 (s, 2F, CF_2Ar), −114.57 (d, *J* = 11.2 Hz, 2F, CF_2CH=); IR (neat): ν 2923, 2870, 1416, 1329, 1220, 1174, 1018, 924, 840 cm^{-1}; HRMS (FAB) calcd for $[M]^+$ $C_{11}H_7F_7$: 272.0436, found 272.0428.

Ethyl 4-(1,1,2,2-tetrafluorobut-3-en-1-yl)benzoate (4f) [S1]

Known compound; Yield: 56% (0.095 g, 0.34 mmol); Colorless oil (hexanes/AcOEt = 20/1, R_f = 0.34); ^{1}H NMR ($CDCl_3$): δ 1.41 (t, *J* = 7.2 Hz, 3H, CH_2CH_3), 4.41 (q, *J* = 7.2 Hz, 2H, CH_2CH_3), 5.71 (d, *J* = 11.2 Hz, 1H, *trans*-CH_2=CHCF_2), 5.83 (dt, *J* = 17.2, 2.0 Hz, 1H, *cis*-CH_2=CHCF_2), 6.01 (ddt, *J* = 17.2, 11.5, 11.2 Hz, 1H, CF_2C*H*=), 7.63 (d, *J* = 8.0 Hz, 2H, Ar*H*), 8.13 (d, *J* = 8.0 Hz, 2H, Ar*H*); ^{13}C NMR ($CDCl_3$): δ 14.6 (CH_2*C*H_3), 61.7 (*C*H_2CH_3), 115.3 (tt, *J* = 249.4, 36.9 Hz, *C*F_2), 116.4 (tt, *J* = 252.3, 35.7 Hz, *C*F_2), 124.8 (t, *J* = 9.3 Hz, *C*H_2=), 126.7 (t, *J* = 24.7 Hz, CF_2*C*H=),

127.3 (t, *J* = 6.4 Hz, Ar), 129.7 (Ar), 133.4 (Ar), 135.2 (t, *J* = 24.8 Hz, Ar), 166.0 (*C*=O); ^{19}F NMR ($CDCl_3$): δ −112.88 (s, 2F, C*F*$_2$Ar), −114.70 (d, *J* = 11.5 Hz, 2F, C*F*$_2$CH=); IR (neat): ν 2985, 1725, 1412, 1279, 1218, 1136, 1076, 981, 855 cm^{-1}; HRMS (FAB) calcd for $[M+H]^+$ $C_{13}H_{13}F_4O_2$: 277.0852, found 277.0853.

3,3,4,4-Tetrafluoro-4-(4-nitrophenyl)but-1-ene (4g) [S1]

Known compound; This compound could not be obtained in a pure form, and included the remaining iodoarene; Yield: 65% (determined by ^{19}F NMR); Colorless oil (hexanes/AcOEt = 5/1, R_f = 0.57; ^{1}H NMR ($CDCl_3$): δ 5.77 (d, *J* = 11.2 Hz, 1H, *trans*-C*H*$_2$=CHCF$_2$), 5.88 (dt, *J* = 17.3, 2.0 Hz, 1H, *cis*-C*H*$_2$=CHCF$_2$), 6.04 (ddt, *J* = 17.3, 11.2, 11.4 Hz, 1H, CF$_2$C*H*=), 7.77 (d, *J* = 8.7 Hz, 2H, Ar*H*), 8.32 (d, *J* = 8.7 Hz, 2H, Ar*H*); ^{13}C NMR ($CDCl_3$): δ 115.0 (tt, *J* = 249.5, 37.1 Hz, *C*F$_2$), 115.8 (tt, *J* = 252.9, 36.5 Hz, *C*F$_2$), 123.6 (Ar), 125.2 (t, *J* = 9.4 Hz, *C*H$_2$=), 126.0 (t, *J* = 24.5 Hz, CF$_2$*C*H=), 128.5 (t, *J* = 6.4 Hz, Ar), 137.0 (t, *J* = 25.0 Hz, Ar), 149.8 (Ar); ^{19}F NMR ($CDCl_3$): δ −112.72 (s, 2F, C*F*$_2$Ar), −114.25 (d, *J* = 11.4 Hz, 2F, C*F*$_2$CH=); IR (neat): ν 3121, 1613, 1533, 1421, 1284, 1242, 1139, 1077, 945, 849 cm^{-1}; HRMS (FAB) calcd for $[M]^+$ $C_{10}H_7F_4NO_2$: 249.0413, found 249.0422.

4-(3-Chlorophenyl)-3,3,4,4-tetrafluorobut-1-ene (4h)

Yield: 60% (0.142 g, 0.60 mmol); Colorless oil (hexanes, R_f = 0.50); ^{1}H NMR ($CDCl_3$): δ 5.72 (d, *J*

= 11.2 Hz, 1H, *trans*-CH_2=CHCF_2), 5.90 (dt, *J* = 17.3, 2.0 Hz, 1H, *cis*- CH_2=CHCF_2), 6.00 (ddt, *J* = 17.3, 11.2, 11.2 Hz, 1H, CF_2C*H*=), 7.40 (dd, *J* = 7.8, 7.8 Hz, 1H, Ar*H*), 7.46 (d, *J* = 7.8 Hz, 1H, Ar*H*) 7.50 (d, *J* = 7.8 Hz, 1H, Ar*H*), 7.56 (s, 1H, Ar*H*); ^{13}C NMR (CDCl_3): δ 115.1 (tt, *J* = 249.2, 37.3 Hz, *C*F_2), 115.8 (tt, *J* = 252.5, 35.7 Hz, *C*F_2), 124.7 (t, *J* = 9.2 Hz, *C*H_2=), 125.3 (t, *J* = 6.5 Hz, Ar), 126.5 (t, *J* = 24.8 Hz, CF_2*C*H=), 127.4 (t, *J* = 6.7 Hz, Ar), 129.8 (Ar), 131.4 (Ar), 132.8 (t, *J* = 25.3 Hz, Ar), 134.6 (Ar); ^{19}F NMR (CDCl_3): δ –112.47 (s, 2F, CF_2Ar), –114.70 (d, *J* = 11.2 Hz, 2F, CF_2CH=); IR (neat): ν 2925, 1420, 1243, 1100, 1011, 981, 886 cm^{-1}; HRMS (FAB) calcd for $[M]^+$ $C_{10}H_7ClF_4$: 238.0172, found 238.0176.

4-(2-Chlorophenyl)-3,3,4,4-tetrafluorobut-1-ene (4i)

Yield: 64% (0.095 g, 0.40 mmol); Colorless oil (hexanes, R_f = 0.50); ^{1}H NMR (CDCl_3): δ 5.73 (d, *J* = 11.3 Hz, 1H, *trans*-CH_2=CHCF_2), 5.87 (dt, *J* = 17.3, 2.2 Hz, 1H, *cis*- CH_2=CHCF_2), 6.09 (ddtm, *J* = 17.3, 11.3, 11.4 Hz, 1H, CF_2C*H*=), 7.35 (tm, *J* = 7.7 Hz, 1H, Ar*H*), 7.42 (tm, *J* = 7.7 Hz, 1H, Ar*H*), 7.48 (d, *J* = 7.7 Hz, 1H, Ar*H*), 7.59 (dm, *J* = 7.7 Hz, 1H, Ar*H*); ^{13}C NMR (CDCl_3): δ 115.6 (tt, *J* = 250.0, 37.3 Hz, *C*F_2), 116.4 (tt, *J* = 254.4, 37.0 Hz, *C*F_2), 124.4 (t, *J* = 9.4 Hz, *C*H_2=), 126.6 (Ar), 126.7 (t, *J* = 24.6 Hz, CF_2C*H*=), 128.4 (t, *J* = 23.7 Hz, Ar), 130.4 (t, *J* = 8.4 Hz, Ar), 132.0 (Ar), 132.2 (Ar), 133.4 (t, *J* = 2.8 Hz, Ar); ^{19}F NMR (CDCl_3): δ –108.47 (s, 2F, CF_2Ar), –113.75 (d, *J* = 11.4 Hz, 2F, CF_2CH=); IR (neat): ν 2929, 1475, 1216, 1154, 1094, 939 cm^{-1}; HRMS (FAB) calcd

for $[M]^+$ $C_{10}H_7ClF_4$: 238.0172, found 238.0174.

Typical procedure for improved Cu(I)-catalyzed cross-coupling reaction of organozinc reagent 2-Zn with ethyl 2-iodobenzoate (3j)

In a two-necked round-bottomed flask, equipped with a teflon-coated stirrer bar and reflux condenser, was added ethyl 2-iodobenzoate (**3j**, 0.14 g 0.50 mmol), Cu_2O (0.060 g, 0.42 mmol), and a DMF solution of **2-Zn** (0.65 M, 1.54 mL, 1.0 mmol), and the mixture was stirred at 100 ºC for 24 h. After being cooled to room temperature, the resulting solution was subjected to a short pad of silica gel using hexanes as an eluent. The filtrate was concentrated in vacuo to give the crude materials, which was purified by silica gel column chromatography to provide the corresponding compound **4j** (0.13 g, 0.48 mmol) in 96% yield as an yellow oil.

Ethyl 2-(1,1,2,2-tetrafluorobut-3-en-1-yl)benzoate (4j)

Yield: 96% (0.13 g, 0.48 mmol); Yellow oil (hexanes/AcOEt = 15/1, R_f = 0.29); ^{1}H NMR ($CDCl_3$): δ 1.35 (t, J = 7.1 Hz, 3H, CH_2CH_3), 4.36 (q, J = 7.1 Hz, 2H, CH_2CH_3), 5.70 (d, J = 11.0 Hz, 1H, *trans*-CH_2=$CHCF_2$), 5.86 (dt, J = 17.3, 2.2 Hz, 1H, *cis*-CH_2=$CHCF_2$), 6.07 (ddt, J = 17.3, 11.4, 11.0 Hz, 1H, CF_2CH=), 7.47–7.62 (m, 4H, Ar*H*); ^{13}C NMR ($CDCl_3$): δ 14.1 (CH_2CH_3), 61.9 (CH_2CH_3), 115.2 (tt, J = 250.2, 37.2 Hz, CF_2), 116.6 (tt, J = 253.4, 36.1 Hz, CF_2), 124.2 (t, J = 9.3 Hz, CH_2=), 126.8 (t, J = 24.4 Hz, CF_2CH=), 127.7 (t, J = 24.5 Hz, Ar), 128.5 (Ar), 128.7–128.9 (m, Ar), 129.7

(Ar), 131.1 (Ar), 133.8 (t, *J* = 3.3 Hz, Ar), 168.6 (*C*=O); ^{19}F NMR ($CDCl_3$): δ −106.44 (s, 2F, C*F*$_2$Ar), −113.42 (d, *J* = 11.4 Hz, 2F, C*F*$_2$CH=); IR (neat): ν 3076, 2988, 2903, 1739, 1578, 1415, 1370, 1269, 1152 cm^{-1}; HRMS (FAB) calcd for $[M+H]^+$ $C_{13}H_{13}F_4O_2$: 277.0852, found 277.0847.

3,3,4,4-Tetrafluoro-4-(2-formylphenyl)but-1-ene (4k)

Yield: 83% (0.096 g, 0.41 mmol); Pale yellow oil (hexanes/AcOEt = 15/1, R_f = 0.40); ^{1}H NMR ($CDCl_3$): δ 5.78 (d, *J* = 11.5 Hz, 1H, *trans*-C*H*$_2$=CHCF$_2$), 5.90 (dt, *J* = 17.3, 2.1 Hz, 1H, *cis*-C*H*$_2$=CHCF$_2$), 6.09 (ddtm, *J* = 17.3, 11.5, 11.4 Hz, 1H, CF$_2$C*H*=), 7.63–7.71 (m, 3H, Ar*H*), 8.11–8.13 (m, 1H, Ar*H*), 10.36 (t, *J* = 1.7 Hz, 1H, C*H*O); ^{13}C NMR ($CDCl_3$): δ 115.1 (tt, *J* = 249.1 Hz, 37.5 Hz, *C*F$_2$), 117.5 (tt, *J* = 253.3, 37.7 Hz, *C*F$_2$), 125.3 (t, *J* = 9.2 Hz, Ar), 126.0 (t, *J* = 24.7 Hz, CF$_2$*C*H=), 128.7 (t, *J* = 9.5 Hz, *C*H$_2$=), 128.8 (Ar), 131.3 (t, *J* = 24.8 Hz, Ar), 131.7 (Ar), 133.1 (Ar), 135.7 (t, *J* = 1.4 Hz, Ar), 191.0 (tt, *J* = 7.3, 3.9 Hz, *C*=O); ^{19}F NMR ($CDCl_3$): δ −103.57 (s, 2F, C*F*$_2$Ar), −114.28 (d, *J* = 11.4 Hz, 2F, C*F*$_2$CH=); IR (neat): ν 3075, 2925, 1699, 1597 1417, 1213, 1128, 922 cm^{-1}; HRMS (FAB) calcd for $[M+H]^+$ $C_{11}H_9F_4O$: 233.0590, found 233.0593.

3,3,4,4-Tetrafluoro-4-(2-acetylphenyl)but-1-ene (4l)

Yield: 81% (0.123 g, 0.50 mmol); Yellow oil (hexane/AcOEt = 5/2, R_f = 0.37); ^{1}H NMR ($CDCl_3$): δ 2.52 (s, 3H, C*H*$_3$), 5.71 (d, *J* = 11.4 Hz, 1H, *trans*-C*H*$_2$=CHCF$_2$), 5.86 (dtm, *J* = 17.3, 2.3 Hz, 1H, *cis*-C*H*$_2$=CHCF$_2$), 6.04 (dtdm, *J* = 17.3, 11.36, 11.42 Hz, 1H, CF$_2$C*H*=), 7.23–7.25 (m, 1H, Ar*H*),

7.47–7.60 (m, 3H, Ar*H*); ^{13}C NMR ($CDCl_3$): δ 31.8 (CH_3), 115.2 (tt, J = 249.8, 37.5 Hz, CF_2), 116.9 (tt, J = 253.0, 36.3 Hz, CF_2), 124.6 (t, J = 9.3 Hz, CH_2=), 125.8 (Ar), 126.1 (t, J = 24.7 Hz, Ar), 126.4 (t, J = 24.4 Hz, CF_2CH=), 128.8 (tt, J = 7.1, 2.6 Hz, Ar), 129.0 (Ar), 131.3 (Ar), 142.6 (t, J = 2.9 Hz, Ar), 203.8 (*C*=O); ^{19}F NMR ($CDCl_3$, $CFCl_3$): δ –105.50 (s, 2F, CF_2Ar), –113.53 (d, J = 11.4 Hz, 2F, CF_2CH=); IR (neat): ν 3415, 3072, 2989, 2942, 1709, 1569, 1412, 1250, 1208, 1143, 1108, 1086, 1054, 941, 922 cm^{-1}; HRMS (FAB) calcd for $[M+H]^+$ $C_{12}H_{11}F_4O$: 247.0746, Found 247.0749.

3,3,4,4-Tetrafluoro-4-(2-methoxymethylphenyl)but-1-ene (4m)

Yield: 58% (0.069 g, 0.28 mmol); Yellow oil (hexanes/AcOEt = 40/1, R_f = 0.31); ^{1}H NMR ($CDCl_3$): δ 3.43 (s, 3H, CH_3), 4.65 (s, 2H, ArCH_2), 5.72 (d, J = 11.24 Hz, 1H, *trans*-CH_2=$CHCF_2$), 5.86 (dt, J = 17.34, 2.15 Hz, 1H, *cis*-CH_2=$CHCF_2$), 6.05 (dtd, J = 17.34, 11.24, 11.37 Hz, 1H, CF_2CH=), 7.36 (t, J = 7.37 Hz, 1H, Ar*H*), 7.49 (d, J = 7.37 Hz, 1H, Ar*H*), 7.52 (t, J = 7.37 Hz, 1H, Ar*H*), 7.70 (d, J = 7.37 Hz, 1H, Ar*H*); ^{13}C NMR ($CDCl_3$): δ 58.5 (CH_3), 71.3 (tt, J = 6.56, 3.28 Hz, ArCH_2), 115.6 (tt, J = 249.3, 38.1 Hz, CF_2), 117.9 (tt, J = 252.98, 36.54 Hz, CF_2), 124.4 (t, J = 9.28 Hz, CH_2=), 126.8 (t, J = 24.97 Hz, CF_2CH=), 127.0 (Ar), 127.7 (t, J = 23.77 Hz, Ar), 128.5 (t, J = 8.09 Hz, Ar), 128.6 (Ar), 131.3 (Ar), 138.6 (Ar); ^{19}F NMR ($CDCl_3$, $CFCl_3$): δ –106.44 (s, 2F, CF_2Ar), –114.28 (d, J = 11.37 Hz, 2F, CF_2CH=); IR (neat): ν 2987, 2931, 2897, 2827, 1452, 1420, 1213, 1196, 1116, 1097,

1061, 960, 938, 918, 765, 687 cm^{-1}; HRMS (FAB) calcd for $[M+Na]^+$ $C_{12}H_{12}F_4NaO$: 271.0722, found 271.0715.

3,3,4,4-Tetrafluoro-4-(2-nitrophenyl)but-1-ene (4n)

Yield: 78% (1.3 g, 5.2 mmol); Yellow oil (hexanes/AcOEt = 5/1, R_f = 0.51); ^{1}H NMR ($CDCl_3$): δ 5.76 (d, *J* = 11.4 Hz, 1H, *trans*-C*H*$_2$=CHCF$_2$), 5.91 (dt, *J* = 17.0, 2.0 Hz, 1H, *cis*-C*H*$_2$=CHCF$_2$), 6.1 (ddtm, *J* = 17.0, 11.4, 11.4 Hz, 1H, CF$_2$C*H*=), 7.55–7.59 (m, 1H, Ar*H*), 7.63–7.72 (m, 3H, Ar*H*); ^{13}C NMR ($CDCl_3$): δ 115.1 (tt, *J* = 250.5, 36.9 Hz, *C*F$_2$), 115.5 (tt, *J* = 254.4, 37.3 Hz, *C*F$_2$), 122.6 (t, *J* = 25.7 Hz, CF$_2$*C*H=), 123.9 (Ar), 125.1 (t, *J* = 9.3 Hz, *C*H$_2$=), 125.9 (t, *J* = 24.4 Hz, Ar), 130.1 (tt, *J* = 6.9, 2.6 Hz, Ar), 131.0 (Ar), 132.6 (Ar), 149.8 (Ar); ^{19}F NMR ($CDCl_3$): δ –107.35 (s, 2F, C*F*$_2$Ar), –113.02 (d, *J* = 11.4 Hz, 2F, C*F*$_2$CH=); IR (neat): ν 3086, 2904, 1609, 1540, 1420, 1372, 1220, 1163, 1119, 980, 851 cm^{-1}; HRMS (FAB) calcd for $[M+Na]^+$ $C_{10}H_7F_4NNaO_2$: 272.0311, found 272.0313.

3,3,4,4-Tetrafluoro-4-(4-fluoro-2-nitrophenyl)but-1-ene (4o)

Yield: 73% (0.390 g, 1.46 mmol); Yellow oil (hexanes/AcOEt = 5/1, R_f = 0.43); ^{1}H NMR ($CDCl_3$): δ 5.74 (d, *J* = 11.18 Hz, 1H, *trans*-C*H*$_2$=CHCF$_2$), 5.87 (dt, *J* = 17.31, 1.84 Hz, 1H, *cis*-C*H*$_2$=CHCF$_2$), 6.03 (dtd, *J* = 17.31, 11.32, 11.18 Hz, 1H, CF$_2$C*H*=), 7.29 (dd, *J* = 7.56, 2.52 Hz, 1H, Ar*H*), 7.33–7.37 (m, 1H, Ar*H*), 7.70 (dd, *J* = 8.92, 5.24 Hz, 1H, Ar*H*); ^{13}C NMR ($CDCl_3$): δ 111.99 (d, *J* =

27.06 Hz, Ar), 115.09 (tt, J = 250.37, 36.98, CF$_2$), 115.37 (tt, J =254.39, 37.51 Hz, CF$_2$), 118.48 (d, J = 21.43 Hz, Ar), 118.70 (Ar), 125.28 (t, J = 9.83 Hz, CH$_2$=), 125.63 (t, J = 24.44 Hz, CF$_2$$C$H=), 132.27 to 132.51 (m, Ar), 150.66 (Ar), 163.86 (d, J = 257.69 Hz, Ar); ^{19}F NMR (CDCl$_3$, CFCl$_3$): δ −104.18 to −104.23 (m, 1F, ArF), −106.94 (s, 2F, CF_2Ar), −112.82 (d, J = 11.32 Hz, 2F, CF_2CH=); IR (neat): ν 3087, 2908, 2351, 1929, 1717, 1621, 1550, 1508, 1421, 1369, 1230, 1117, 953, 729 cm^{-1}; HRMS (FAB) calcd for [M]$^+$ $C_{10}H_6F_5NO_2$: 267.0139, found 267.0134.

3,3,4,4-Tetrafluoro-4-(4-methoxy-2-nitrophenyl)but-1-ene (4p)

Yield: 90% (0.510 g, 1.83 mmol); Yellow oil (hexanes/AcOEt = 5/1, R$_f$ = 0.37); ^{1}H NMR (CDCl$_3$): δ 3.83 (s, 3H, CH_3), 5.70 (d, J = 11.24 Hz, 1H, *trans*-CH_2=CHCF$_2$), 5.83 (dt, J = 17.33, 1.80 Hz, 1H, *cis*-CH_2=CHCF$_2$), 6.00 (dtd, J = 17.33, 11.67, 11.24 Hz, 1H, CF$_2$CH=), 7.00 (d, J = 2.52 Hz, 1H, ArH), 7.08 (dm, J = 8.94 Hz, 1H, ArH), 7.54 (d, J = 8.94 Hz, 1H, ArH); ^{13}C NMR (CDCl$_3$): δ 55.99 (CH$_3$), 109.47 (Ar), 113.77 (t, J = 26.23 Hz, Ar), 115.14 (tt, J = 250.09, 37.36 Hz, CF$_2$), 115.65 (tt, J = 253.52, 36.90 Hz, CF$_2$), 116.30 (Ar), 124.78 (t, J = 9.31 Hz, CH$_2$=), 125.98 (t, J = 24.43 Hz, CF$_2$$C$H=), 131.25 (t, J = 7.05 Hz, Ar), 150.68 (Ar), 162.28 (Ar); ^{19}F NMR (CDCl$_3$, CFCl$_3$): δ −106.74 (s, 2F, CF_2Ar), −113.17 (d, J = 11.67 Hz, 2F, CF_2CH=); IR (neat): ν 3095, 2935, 2850, 2370, 2313, 1621, 1546, 1421, 1371, 1098, 1055, 795 cm^{-1}; HRMS (FAB) calcd for [M+Na]$^+$ $C_{11}H_9F_4NNaO_3$: 302.0417, found 302.0418.

3,3,4,4-Tetrafluoro-4-(5-methoxy-2-nitrophenyl)but-1-ene (4q)

Yield: 83% (0.21 g, 0.75 mmol); Yellow oil (hexanes/AcOEt = 5/1, R_f = 0.29); ^{1}H NMR ($CDCl_3$): δ 3.87 (s, 3H, CH_3), 5.73 (d, J = 11.22 Hz, 1H, *trans*-CH_2=CHCF_2), 5.87 (d, J = 17.29 Hz, 1H, *cis*-CH_2=CHCF_2), 6.05 (dtd, J = 17.29, 11.40, 11.22 Hz, 1H, C*H*CF_2=), 7.06 (dd, J = 8.92, 2.50 Hz, 1H, ArH), 7.12 (d, J = 2.50 Hz, 1H, ArH), 7.59 (d, J = 8.92 Hz, 1H, ArH); ^{13}C NMR ($CDCl_3$): δ 56.1 (*C*H_3), 115.2 (tt, J = 250.8, 36.6 Hz, *C*F_2), 115.4 (tt, J = 254.5, 37.1 Hz, *C*F_2), 115.7 (tt, J = 7.8, 2.4 Hz, Ar), 116.5 (Ar), 142.8 (t, J = 21.48 Hz, *C*HCF_2=), 125.0 (t, J = 8.96 Hz, *C*H_2=), 126.0 (t, J = 24.29 Hz, Ar), 126.5 (Ar), 143.1 (Ar), 161.2 (Ar); ^{19}F NMR ($CDCl_3$, $CFCl_3$): δ –106.19 (s, 2F, CF_2Ar), –112.46 (d, J = 11.40 Hz, 2F, CF_2CH=); IR (neat): ν 2947, 2849, 1588, 1538, 1498, 1421, 1364, 1302, 1248, 1117, 963, 859 cm^{-1}; HRMS (FAB) calcd for $[M+H]^+$ $C_{11}H_{10}F_4NNaO_3$: 280.0597, found 280.0599.

3-(1,1,2,2-Tetrafluorobut-3-en-1-yl)pyridine (4r) [S1]

Known compound: Yield: 57% (0.070 g, 0.34 mmol); Yellow oil (hexanes/AcOEt = 5/1, R_f = 0.29); ^{1}H NMR ($CDCl_3$): δ 5.74 (d, J = 11.3 Hz, 1H, *trans*-CH_2=CHCF_2), 5.86 (dt, J = 17.2, 2.1 Hz, 1H, *cis*-CH_2=CHCF_2), 6.04 (ddt, J = 17.2, 11.5, 11.3 Hz, 1H, CF_2C*H*=), 7.41 (dd, J = 8.0, 4.5 Hz, 1H, Ar*H*), 7.87 (d, J = 8.0 Hz, 1H, Ar*H*), 8.76 (d, J = 4.5 Hz, 1H, Ar*H*), 8.80 (s, 1H, Ar*H*); ^{13}C NMR ($CDCl_3$): δ 115.0 (tt, J = 249.2, 37.4 Hz, *C*F_2), 115.8 (tt, J = 252.4, 36.6 Hz, *C*F_2), 123.2 (Ar), 125.1

(t, J = 9.4 Hz, CH_2=), 126.1 (t, J = 24.6 Hz, CF_2CH=), 127.0 (t, J = 24.9 Hz, Ar), 134.8 (t, J = 6.2 Hz, Ar), 148.2 (t, J = 6.8 Hz, Ar), 152.0 (Ar); ^{19}F NMR ($CDCl_3$): δ −113.14 (s, 2F, CF_2Ar), −114.62 (d, J = 11.5 Hz, 2F, CF_2CH=); IR (neat): ν 2928, 2858, 1728, 1424, 1292, 1114, 1094, 965 cm^{-1}; HRMS (FAB) calcd for $[M+H]^+$ $C_9H_8F_4N$: 206.0593, found 206.0586.

Typical procedure for the Amination of 4n

To a solution of **4n** (0.90 g, 3.6 mmol) in EtOH (5.4 mL) and H_2O (1.8 mL) was added NH_4Cl (0.58 g, 11 mmol) and Fe (1.4 g, 25 mmol). The mixture was heated to reflux (bath temp. 85 ºC) and stirred for 16 h. The reaction mixture was cooled to room temperature. Next, the mixture was filtered through silica gel with ethyl acetate, and then concentrated. The **4s** was purified by silica gel column chromatography (0.68 g, 3.1 mmol, 87%).

2-(1,1,2,2-Tetafluorobut-3-en-1-yl)aniline (4s)

Yield: 87% (0.682 g, 3.11 mmol); Yellow oil (hexanes/AcOEt = 5/1, R_f = 0.50); ^{1}H NMR ($CDCl_3$): δ 4.17 (s, 2H, NH_2), 5.70 (d, J = 11.2 Hz, 1H, *trans*-CH_2=$CHCF_2$), 5.87 (dtd, J = 17.4, 2.1, 0.7 Hz, 1H, *cis*-CH_2=$CHCF_2$), 6.03 (dtd, J = 17.4, 11.3, 11.2 Hz, 1H, $CHCF_2$=), 6.69 (dd, J = 7.9, 0.6 Hz, 1H, ArH), 6.77 (tm, J = 7.9 Hz, 1H, ArH), 7.23–7.30 (m, 2H, ArH); ^{13}C NMR ($CDCl_3$): δ 113.3 (t, J = 22.9 Hz, =$CHCF_2$), 116.3 (tt, J = 249.7, 37.9 Hz, CF_2), 117.57 (Ar), 117.64 (Ar), 118.3 (tt, J = 252.1, 35.9 Hz, CF_2), 124.2 (t, J = 9.3 Hz, CH_2=), 126.9 (t, J = 24.6 Hz, Ar), 129.2 (t, J = 8.4 Hz,

Ar), 132.2 (Ar), 145.7 (t, J = 2.2 Hz, Ar); ^{19}F NMR ($CDCl_3$): δ −110.60 (s, 2F, CF_2Ar), −115.03 (d, J = 11.3 Hz, 2F, CF_2CH=); IR (neat): ν 3522, 3421, 3236, 3042, 1925, 1582, 1495, 1467, 1420, 1322 cm^{-1}; HRMS (FAB) calcd for $C_{10}H_9F_4N$ [M]$^+$: 219.0671, found 219.0668.

Typical procedure for the Sandmayer reaction of 4s

Under an argon atmosphere, to concentrated aqueous HCl (0.75 mL) was slowly added aniline derivative **4s** (0.11 g, 0.49 mmol) and distilled water (0.75 mL) at 0 ºC and stirring for 30 min at the same temperature. Then, to this mixture was added a solution of $NaNO_2$ dissolved in H_2O (1.5 mL) at the 0 ºC. After stirring for 10 min at the same temperature, to this mixture was added a solution of KI dissolved in H_2O (1.5 mL) at 0 ºC. After gradually warming to room temperature, the mixture was quenched with saturated aqueous sodium thiosulfate and then whole was extracted with ethyl acetate three times. The combined organic layers were dried over anhydrous Na_2SO_4, then filtered, and concentrated in *vacuo.* The residue was purified by silica gel column chromatography to give the corresponding iodide **4t** (0.11 g, 0.33 mmol) in 67% yield as a pale yellow oil.

3,3,4,4-Tetrafluoro-4-(2-iodophenyl)but-1-ene (4t)

Yield: 67% (0.11 g, 0.33 mmol); pale yellow oil (hexanes only, R_f = 0.50); ^{1}H NMR ($CDCl_3$): δ 5.73 (d, J = 11.2 Hz, 1H, *trans*-CH_2=CHCF$_2$), 5.87 (dt, J = 17.3, 2.1 Hz, 1H, *cis*-CH_2=CHCF$_2$), 6.07 (dtd, J = 17.3, 11.7, 11.2 Hz, 1H, C*H*CF$_2$=), 7.11–7.15 (m, 1H, Ar*H*), 7.41–7.46 (m, 1H, Ar*H*),

7.54 (d, J = 7.9 Hz, 1H, ArH), 8.06 (d, J = 7.9 Hz, 1H, ArH); ^{13}C NMR ($CDCl_3$): δ 91.4 (Ar), 115.49 (tt, J = 250.4, 37.4 Hz, CF_2), 115.54 (tt, J = 254.9, 36.8 Hz, CF_2), 124.6 (t, J = 9.4 Hz, CH_2=), 126.8 (t, J = 24.5 Hz, =CHCF$_2$), 127.7, 130.5 (t, J = 8.4 Hz), 132.2, 133.2 (t, J = 23.5 Hz), 142.9; ^{19}F NMR ($CDCl_3$): δ −107.37 (s, 2F, CF_2Ar), −112.46 (d, J = 11.7 Hz, 2F, CF_2CH=); IR (neat): ν 3086, 2928, 2850, 1934, 1818, 1716, 1650, 1589, 1567, 1467, 1420 cm^{-1}; HRMS (FAB) calcd for $[M]^+$ $C_{10}H_7F_4I$: 329.9529, found 329.9520.

Typical procedure for the Sonogashira cross-coupling reaction of 4s with phenylacetylene

To a solution of **4t** (0.15 g, 0.45 mmol) in Et_3N (0.66 mL) was added $Cl_2Pd(PPh_3)_2$ (0.016 g, 0.0225 mmol), CuI (0.043 g, 0.225 mmol) and ethynylbenzene (0.15 mL 1.35 mmol). The mixture was stirred overnight at the same temperature. Next, the mixture was filtered through silica gel with hexane, and then concentrated. The crude mixture was purified by silica gel column chromatography to afford the corresponding **4u** in a pure form (0.094 g, 0.31 mmol, 69%).

1-(2-Phenylethynyl)-2-(2,2,3,3-tetrafluorobut-3-en-1-yl)benzene (4u)

Yield: 69% (0.094 g, 0.31 mmol); Yellow oil (hexanes/AcOEt = 20/1, R_f = 0.41); ^{1}H NMR ($CDCl_3$): δ 5.70 (d, J = 11.3 Hz, 1H, *trans*-CH_2=CHCF$_2$), 5.87 (dt, J = 16.9, 1.6 Hz, 1H, *cis*-CH_2=CHCF$_2$), 6.13 (ddt, J = 16.9, 11.3, 11.2 Hz, 1H, CHCF$_2$=), 7.37–7.68 (m, 9H, ArH); ^{13}C NMR ($CDCl_3$): δ 87.5 (C≡), 93.6 (C≡), 115.7 (tt, J = 250.4, 36.8 Hz, CF_2), 116.8 (tt, J = 254.5, 35.7 Hz, CF_2), 122.7

(t, *J* = 3.5 Hz, Ar), 123.4 (Ar), 124.1 (t, *J* = 9.3 Hz, *C*H_2=CH-), 127.1 (t, *J* = 24.5 Hz, *C*F_2CH=), 127.9 (Ar), 128.5 (Ar), 128.6 (Ar), 128.7 (Ar), 130.8 (Ar), 131.61 (Ar), 131.62 (t, *J* = 22.8 Hz, Ar), 134.3 (Ar); ^{19}F NMR ($CDCl_3$): δ –109.39 (s, 2F, CF_2Ar), –114.24 (d, *J* = 11.2 Hz, 2F, CF_2CH=); IR (neat): ν 3064, 2957, 2926, 2857, 2338, 2224, 1971, 1947, 1918, 1883, 1831, 1725, 1647, 1603, 1498, 1126 cm^{-1}; HRMS (FAB) calcd for $[M]^+$ $C_{18}H_{12}F_4$: 304.0875, found 304.0866.

Typical procedure for Cu(I)-catalyzed cross-coupling reaction of organozinc reagent 2-Zn with benzoyl chloride (5a)

In a two-necked round-bottomed flask, equipped with a teflon-coated stirrer bar, were placed benzoyl chloride (**5a**, 0.17 mL, 1.5 mmol), Cu_2O (0.027 g, 0.18 mmol), 2,2'-bipyridyl (0.019 g, 0.12 mmol), and a DMF solution of **2-Zn** (0.61 M, 1.0 mL, 0.61 mmol), and the whole were stirred at room temperature for 20 h. The resultant was poured into water and the organic products were extracted with Et_2O three times. The combined organic layers were dried over anhydrous Na_2SO_4, then filtered, and concentrated in vacuo. The residue was purified by silica gel column chromatography to give the corresponding **6a** (0.13 g, 0.56 mmol) in 92% yield as a colorless oil.

2,2,3,3-Tetrafluoro-1-phenylpent-4-en-1-one (6a)

Yield: 92% (0.13 g, 0.56 mmol); Colorless oil (hexanes/AcOEt = 5/1, R_f = 0.70); ^{1}H NMR ($CDCl_3$): δ 5.76 (d, *J* = 10.4 Hz, 1H, *cis*-CH_2=CH), 5.93 (dt, *J* = 17.6, 2.4 Hz, 1H, *trans*-CH_2=CH), 6.06–6.19

(m, 1H, CH_2=C*H*), 7.50–7.54 (m, 2H, Ar-*H*), 7.67 (t, *J* = 7.6 Hz 1H, Ar-*H*), 8.09 (d, *J* = 6.5 Hz 2H, Ar-*H*); ^{13}C NMR ($CDCl_3$): δ 112.3 (tt, *J* = 264.5, 36.3 Hz, *C*F_2), 115.0 (tt, *J* = 250.4, 31.4 Hz, *C*F_2), 125.1 (t, *J* = 12.0 Hz, *C*H_2=), 126.6 (t, *J* = 24.0 Hz, CH_2=*C*H), 129.1 (Ar), 130.6 (m, Ar), 132.6 (m, Ar), 135.2 (Ar), 186.4 (t, *J* = 26.4 Hz, *C*=O); ^{19}F NMR ($CDCl_3$): δ –114.76 (s, 2F, C*F_2*-C=O), –113.90 (d, *J* = 12.0 Hz, 2F, =CH-C*F_2*); IR (neat): ν 3066, 2929, 1704, 1450, 1308, 1239, 1153, 1012, 965, 848 cm^{-1}; HRMS (FAB) calcd for $C_{11}H_9F_4O$ [M+H]$^+$: 233.0590, found 233.0595.

2,2,3,3-Tetrafluoro-1-(4-methylphenyl)pent-4-en-1-one (6b)

Yield: 97% (0.15 g, 0.59 mmol); Colorless oil (hexanes/AcOEt = 5/1, R_f = 0.70); ^{1}H NMR ($CDCl_3$): δ 2.42 (s, 3H, C*H_3*), 5.73 (d, *J* = 11.1 Hz, 1H, *cis*-C*H_2*=CH), 5.92 (dt, *J* = 17.1, 2.2 Hz, 1H, *trans*-C*H_2*=CH), 6.11 (ddt, *J* = 17.1, 11.6, 11.1 Hz, 1H, CH_2=C*H*), 7.29 (d, *J* = 8.1 Hz, 2H, Ar-*H*), 8.00 (d, *J* = 8.1 Hz, 2H, Ar-*H*); ^{13}C NMR ($CDCl_3$): δ 21.8 (*C*H_3), 112.2 (tt, *J* = 265.3, 35.5 Hz, *C*F_2), 114.8 (tt, *J* = 250.4, 31.0 Hz, *C*F_2), 124.7 (t, *J* = 9.5 Hz, *C*H_2=), 126.5 (t, *J* = 23.6 Hz, CH_2=*C*H), 129.6 (Ar), 129.9 (Ar), 130.5 (t, *J* = 3.3 Hz, Ar), 146.6 (Ar), 185.6 (t, *J* = 26.9 Hz, *C*=O); ^{19}F NMR ($CDCl_3$): δ –114.71 (s, 2F, C*F_2*-C=O), –113.96 (d, *J* = 11.6 Hz, 2F, =CH-C*F_2*); IR (neat): ν 2925, 1699, 1420, 1308, 1232, 1153, 1112, 1012, 856 cm^{-1}; HRMS (FAB) calcd for $C_{12}H_{11}F_4O$ [M+H]$^+$: 247.0746, found 247.0741.

2,2,3,3-Tetrafluoro-1-(2-methylphenyl)pent-4-en-1-one (6c)

Yield: 83% (0.13 g, 0.53 mmol); Pale yellow oil (hexanes/AcOEt = 20/1, R_f = 0.60); ^{1}H NMR ($CDCl_3$): δ 2.46 (s, 3H, CH_3), 5.75 (d, J = 11.3 Hz, 1H, *cis*-CH_2=CH), 5.91 (dt, J = 17.4, 2.2 Hz, 1H, *trans*-CH_2=CH), 6.12 (ddtm, J = 17.4, 11.6, 11.3 Hz, 1H, CH_2=C*H*), 7.29–7.33 (m, 2H, Ar-*H*), 7.45–7.49 (m, 1H, Ar-*H*), 7.79–7.81 (m, 1H, Ar-*H*); ^{13}C NMR ($CDCl_3$): δ 20.8 (CH_3), 115.1 (tt, J = 266.6, 35.8 Hz, CF_2), 114.9 (tt, J = 250.5, 31.5 Hz, CF_2), 124.6 (t, J = 9.6 Hz, CH_2=), 125.5 (Ar), 126.4 (t, J = 23.9 Hz, CH_2=*C*H), 129.3 (t, J = 5.6 Hz, Ar), 132.0 (Ar), 132.8 (m, 2C, Ar), 140.0 (Ar), 189.6 (t, J = 26.8 Hz, *C*=O); ^{19}F NMR ($CDCl_3$): δ –113.76 (m, 2F, CF_2-C=O), –115.08 (s, 2F, =CH-CF_2); IR (neat): ν 3030, 2966, 1715, 1420, 1292, 1247, 1156, 1111, 1013, 860 cm^{-1}; HRMS (FAB) calcd for $C_{12}H_{10}F_4O$ $[M]^+$: 246.0663, found 246.0668.

1-(4-*tert*-Butylphenyl)-2,2,3,3-tetrafluoropent-4-en-1-one (6d)

Yield: 98% (0.17 g, 0.59 mmol); Pale yellow oil (hexanes/AcOEt = 20/1, R_f = 0.60); ^{1}H NMR ($CDCl_3$): δ 1.35 (s, 9H, CH_3), 5.74 (d, J = 11.3 Hz, 1H, *cis*-CH_2=CH), 5.92 (dt, J = 17.4, 2.2 Hz, 1H, *trans*-CH_2=CH), 6.12 (ddt, J = 17.4, 11.4, 11.3 Hz, 1H, CH_2=C*H*), 7.53 (dt, J = 8.7, 2.1 Hz, 2H, Ar-*H*), 8.05 (d, J = 8.7 Hz, 2H, Ar-*H*); ^{13}C NMR ($CDCl_3$): δ 31.1 (CH_3), 35.5 ($C(CH_3)_3$), 112.2□(tt, J = 265.2, 35.7 Hz, CF_2), 114.8 (tt, J = 250.5, 31.1 Hz, CF_2), 124.8 (t, J = 9.5 Hz, CH_2=) , 125.9 (Ar), 126.5 (t, J = 23.9 Hz, CH_2=*C*H), 129.8 (Ar), 130.5 (t, J = 3.4 Hz, Ar), 159.2 (Ar), 185.7 (t, J =

26.8 Hz, *C*=O); ^{19}F NMR ($CDCl_3$): δ –113.98 (dd, *J* = 11.4, 2.2 Hz, 2F, CF_2-C=O), –114.76 (s, 2F, =CH-CF_2); IR (neat): ν 2970, 1700, 1600, 1415, 1304, 1114, 888 cm^{-1}; HRMS (FAB) calcd for $C_{15}H_{17}F_4O$ [M+H]$^+$: 289.1216, found 289.1208.

1-(4-Bromophenyl)-2,2,3,3-tetrafluoropent-4-en-1-one (6e)

Yield: 50% (0.095 g, 0.31 mmol); Colorless oil (hexanes/AcOEt = 20/1, R_f = 0.50); ^{1}H NMR ($CDCl_3$): δ 5.77 (d, *J* = 11.2 Hz, 1H, *cis*-CH_2=CH), 5.93 (dt, *J* = 17.3, 2.1 Hz, 1H, *trans*-CH_2=CH), 6.10 (ddt, *J* = 17.3, 11.4, 11.2 Hz, 1H, CH_2=C*H*), 7.64–7.70 (m, 2H, Ar-*H*), 7.95 (d, *J* = 8.6 Hz, 2H, Ar-*H*); ^{13}C NMR ($CDCl_3$): δ 111.9 (tt, *J* = 265.1, 36.3 Hz, *C*F_2), 114.6 (tt, *J* = 250.5, 31.2 Hz, *C*F_2), 125.0 (t, *J* = 9.5 Hz, *C*H_2=), 126.0 (t, *J* = 23.9 Hz, CH_2=*C*H), 130.7 (Ar), 131.0 (Ar), 131.7 (t, *J* = 3.5 Hz, Ar), 132.2 (Ar), 185.1 (t, *J* = 27.3 Hz, *C*=O); ^{19}F NMR ($CDCl_3$): δ −114.89 (s, 2F, CF_2-C=O), −113.80 (dd, *J* = 11.4, 2.1 Hz, 2F, =CH-CF_2); IR (neat): ν 3125, 1701, 1584, 1420, 1284, 1112, 1012, 883 cm^{-1}; HRMS (FAB) calcd for $C_{11}H_8BrF_4O$ [M+H]$^+$: 310.9695, found 310.9689.

2,2,3,3-Tetrafluoro-1-(4-trifluoromethylphenyl)pent-4-en-1-one (6f)

Yield: 86% (0.15 g, 0.50 mmol), Pale yellow oil (hexanes/AcOEt = 20/1, R_f = 0.40); ^{1}H NMR ($CDCl_3$): δ 5.79 (d, *J* = 11.1 Hz, 1H, *cis*-CH_2=CH), 5.95 (dt, *J* = 17.3, 2.2 Hz, 1H, *trans*-CH_2=CH), 6.11 (ddt, *J* = 17.3, 11.9, 11.1 Hz, 1H, CH_2=C*H*), 7.78 (d, *J* = 8.2 Hz, 2H, Ar-*H*), 8.20 (d, *J* = 8.2

Hz, 2H, Ar-*H*); ^{13}C NMR ($CDCl_3$): δ 111.9 (tt, *J* = 265.2, 36.4 Hz, *C*F_2), 114.8 (tt, *J* = 250.6, 31.2 Hz, *C*F_2), 123.4 (q, *J* = 272.9 Hz, *C*F_3), 125.5 (t, *J* = 9.7 Hz, *C*H_2=), 126.95 (t, *J* = 23.2 Hz, CH_2=*C*H), 125.96 (q, *J* = 3.6 Hz, Ar), 130.8 (t, *J* = 3.7 Hz, Ar), 135.1 (Ar), 136.1 (q, *J* = 27.6 Hz, Ar), 185.6 (t, *J* = 27.56 Hz, *C*=O); ^{19}F NMR ($CDCl_3$): δ –115.05 (s, 2F, CF_2-C=O), –113.63 (dd, *J* = 11.9, 2.2 Hz, 2F, =CH-CF_2), –64.01 (s, 3F, CF_3); IR (neat): ν 3125, 3075, 1714, 1414, 1330, 1234, 1136, 1069, 965, 852 cm^{-1}; HRMS (FAB) calcd for $C_{12}H_7F_7O$ $[M]^+$: 300.0385, found 300.0393.

2,2,3,3-Tetrafluoro-1-(furan-2-yl)pent-4-en-1-one (6h)

Yield: 83% (0.11 g, 0.50 mmol); Pale yellow oil (hexanes/AcOEt = 5/1, R_f = 0.40); ^1H NMR ($CDCl_3$): δ 5.69 (d, *J* = 11.0 Hz, 1H, *cis*-CH_2=CH), 5.83 (dt, *J* = 17.0, 2.2 Hz, 1H, *trans*-CH_2=CH), 5.97 (ddt, *J* = 17.0, 11.8, 11.0 Hz, 1H, CH_2=C*H*), 6.59 (dd, *J* = 3.8, 1.7 Hz, 1H, Ar-*H*), 7.43–7.45 (m, 1H, Ar-*H*), 7.74 (dd, *J* = 1.7, 0.6 Hz, 1H, Ar-*H*); ^{13}C NMR ($CDCl_3$): δ 111.4 (tt, *J* = 263.3, 36.7 Hz, *C*F_2), 113.2 (Ar), 114.5 (tt, *J* = 250.5, 32.0 Hz, *C*F_2), 124.7 (t, *J* = 5.8 Hz, Ar), 125.1 (t, *J* = 9.5 Hz, *C*H_2=), 125.6 (t, *J* = 23.8 Hz, CH_2=*C*H), 148.6 (Ar), 149.6 (Ar), 173.3 (t, *J* = 28.0 Hz, *C*=O); ^{19}F NMR ($CDCl_3$): δ –118.56 (s, 2F, CF_2-C=O), –114.19 (d, *J* = 11.8 Hz, 2F, =CH-CF_2); IR (neat): ν 3150, 1692, 1560, 1461, 1397, 1186, 1041, 931, 844 cm^{-1}; HRMS (FAB) calcd for $C_9H_7F_4O_2$ $[M+H]^+$: 223.0382, found 223.0392.

2,2,3,3-Tetrafluoro-1-(thiophen-2-yl)pent-4-en-1-one (6i)

Yield: 87% (0.13 g, 0.55 mmol); Colorless oil (hexanes/AcOEt = 20/1, R_f = 0.34); ^{1}H NMR ($CDCl_3$): δ 5.76 (d, J = 11.1 Hz, 1H, *cis*-CH_2=CH), 5.92 (dt, J = 17.3, 2.1 Hz, 1H, *trans*-CH_2=CH), 6.08 (ddt, J = 17.3, 11.4, 11.1 Hz, 1H, CH_2=C*H*), 7.21–7.23 (m, 1H, Ar-*H*), 7.85 (dd, J = 4.9, 1.0 Hz, 1H, Ar-*H*), 8.00–8.01 (m, 1H, Ar-*H*); ^{13}C NMR ($CDCl_3$): δ 114.7 (tt, J = 263.3, 36.4 Hz, *C*F_2), 114.6 (tt, J = 250.8, 30.8 Hz, *C*F_2), 125.1 (t, J = 9.5 Hz, *C*H_2=), 125.9 (t, J = 23.9 Hz, CH_2=*C*H), 129.1 (Ar), 136.8 (t, J = 5.5 Hz, Ar), 137.6 (Ar), 138.8 (Ar), 178.8 (t, J = 27.8 Hz, *C*=O); ^{19}F NMR ($CDCl_3$): δ –114.03 to –113.99 (m, 2F, CF_2-C=O), –116.59 (s, 2F, =CH-CF_2); IR (neat): ν 3104, 1676, 1411, 1359, 1238, 1165, 1115, 1065, 820 cm^{-1}; HRMS (FAB) calcd for $C_9H_7F_4OS$ $[M+H]^+$: 239.0153, found 239.0154.

4,4,5,5-Tetrafluoro-1-phenylhept-1,6-diene-3-one (6j)

Yield: 70% (0.11 g, 0.43 mmol); Pale yellow oil (hexanes/AcOEt = 40/1, R_f = 0.37); ^{1}H NMR ($CDCl_3$): δ 5.76 (d, J = 10.4 Hz, 1H, *cis*-CH_2=CH), 5.89–5.95(m, 1H, *trans*-CH_2=CH), 6.05 (ddtm, J = 15.5, 13.1, 10.4 Hz, 1H, CH_2=C*H*), 7.15 (d, J = 15.6 Hz, 1H), 7.42–7.50 (m, 3H, Ar-*H*), 7.63–7.66 (m, 2H, Ar-*H*), 7.92 (d, J = 15.9 Hz, 1H); ^{13}C NMR ($CDCl_3$): δ = 111.1 (tt, J = 262.9, 36.8 Hz, *C*F_2), 114.9 (tt, J = 249.7, 32.2 Hz, *C*F_2), 118.4, 125.1 (t, J = 9.5 Hz, *C*H_2=), 125.9 (t, J = 24.0 Hz, CH_2=*C*H), 129.28, 129.34, 132.1, 133.8, 148.5, 185.1 (t, J = 26.5 Hz, *C*=O); ^{19}F NMR ($CDCl_3$): δ –

114.48 (d, J = 13.1 Hz, 2F, CF_2-C=O), –122.74 (s, 2F, =CH-CF_2); IR (neat): ν 3068, 2926, 1707, 1608, 1577, 1420, 1147, 1066, 980 cm^{-1}; HRMS (FAB) calcd for $C_{13}H_{11}F_4O$ $[M+H]^+$: 259.0744, found 259.0746.

3,3,4,4-Tetrafluorododec-1-en-5-one (6k)

Yield: 84% (0.13 g, 0.84 mmol); Colorless oil (hexanes/AcOEt = 20/1, R_f = 0.57); ^{1}H NMR ($CDCl_3$): δ 0.85 (brs, 3H, CH_3), 1.27 (brs, 8H, -$(CH_2)_4$-), 1.61 (brs, 2H, $COCH_2CH_2$), 2.70 (t, J = 6.7 Hz, 2H, $COCH_2CH_2$), 5.69 (d, J = 10.5 Hz, 1H, *cis*-CH_2=CH), 5.84 (d, J = 17.3 Hz, 1H, *trans*-CH_2=CH), 5.96 (dtd, J = 17.3, 11.4, 10.5 Hz, 1H, CH_2=CH); ^{13}C NMR ($CDCl_3$): δ 14.0 (CH_3), 22.6 (CH_2), 22.7 (CH_2), 28.9 (CH_2), 29.1 (CH_2), 31.8 (CH_2), 38.3 (CH_2), 110.5 (tt, J = 263.6, 37.3 Hz, CF_2), 114.9 (tt, J = 249.4, 32.4 Hz, CF_2), 124.8 (t, J = 9.6 Hz, CH_2=CH), 125.9 (t, J = 23.9 Hz, =CH-CF_2), 197.2 (t, J = 26.7 Hz, C=O); ^{19}F NMR ($CDCl_3$): δ –122.10 (s, 2F, CF_2-C=O), –114.52 (d, J = 11.4 Hz, 2F, =CH-CF_2); IR (neat): ν 2931, 2859, 1751, 1420, 1153, 1117, 1012, 962 cm^{-1}; HRMS (FAB) calcd for $C_{12}H_{19}F_4O$ $[M+H]^+$: 255.1372, found 255.1364.

Typical procedure for improved Cu(I)-catalyzed cross-coupling reaction of organozinc reagent 2-Zn with 4-nitrobenzoyl chloride (5g)

In a two-necked round-bottomed flask, equipped with a teflon-coated stirrer bar, were placed 4-nitrobenzoyl chloride (**5g**, 0.50 g 2.7 mmol), Cu_2O (0.030 g, 0.21 mmol), 2,2'-bipyridyl (0.020 g,

0.13 mmol), and a DMF solution of **2-Zn** (0.66 M, 1.0 mL, 0.66 mmol), and the whole were stirred at 100 °C for 20 h. The resultant was poured into water and the organic products were extracted with Et_2O three times. The combined organic layers were dried over anhydrous Na_2SO_4, then filtered, and concentrated in *vacuo*. The residue was purified by silica gel column chromatography to give the corresponding **6g** (0.14 g, 0.51 mmol) in 77% yield as a pale yellow oil.

2,2,3,3-Tetrafluoro-1-(4-nitrophenyl)pent-4-en-1-one (6g)

Yield: 77% (0.14 g, 0.51 mmol); Pale yellow oil (hexanes/AcOEt = 5/1, R_f = 0.50); ^{1}H NMR ($CDCl_3$): δ 5.78 (d, J = 11.0 Hz, 1H, *cis*-CH_2=CH), 5.91 (dt, J = 17.31, 1.68 Hz, 1H, *trans*-CH_2=CH), 6.07 (dtd, J = 17.3, 11.9, 11.0 Hz, 1H, CH_2=C*H*), 8.21 (d, J = 8.4 Hz, 2H, Ar-*H*), 8.30–8.33 (m, 2H, Ar-*H*); ^{13}C NMR ($CDCl_3$): δ 111.7 (tt, J = 264.9, 37.4 Hz, *C*F_2), 114.7 (tt, J = 250.6, 31.4 Hz, *C*F_2), 123.9 (Ar), 125.6 (t, J = 23.9 Hz, CH_2=*C*H), 125.6 (t, J = 9.4 Hz, *C*H_2=), 131.4 (t, J = 3.4 Hz, Ar), 136.7 (Ar), 151.2 (Ar), 185.1 (t, J = 28.0 Hz, *C*=O); ^{19}F NMR ($CDCl_3$): δ –115.04 (s, 2F, CF_2-C=O), –113.37 (d, J = 11.9 Hz, 2F, =CH-CF_2); IR (neat): ν 3057, 1715, 1604, 1531, 1351, 1156, 1014, 834 cm^{-1}; HRMS (FAB) calcd for $C_{11}H_8F_4NO_3$ $[M+H]^+$: 278.0440, found 278.0435.

2. Additional scheme and Figure

Scheme S1:

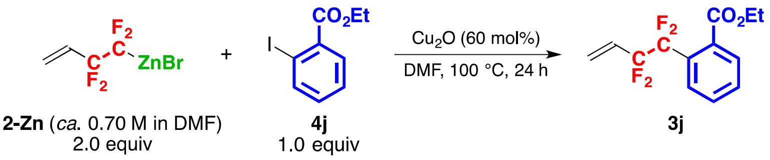

Condition	^{19}F NMR Yield/%
Freshly prepared	quant.
1.5 year after the zinc reagent was prepared	quant.

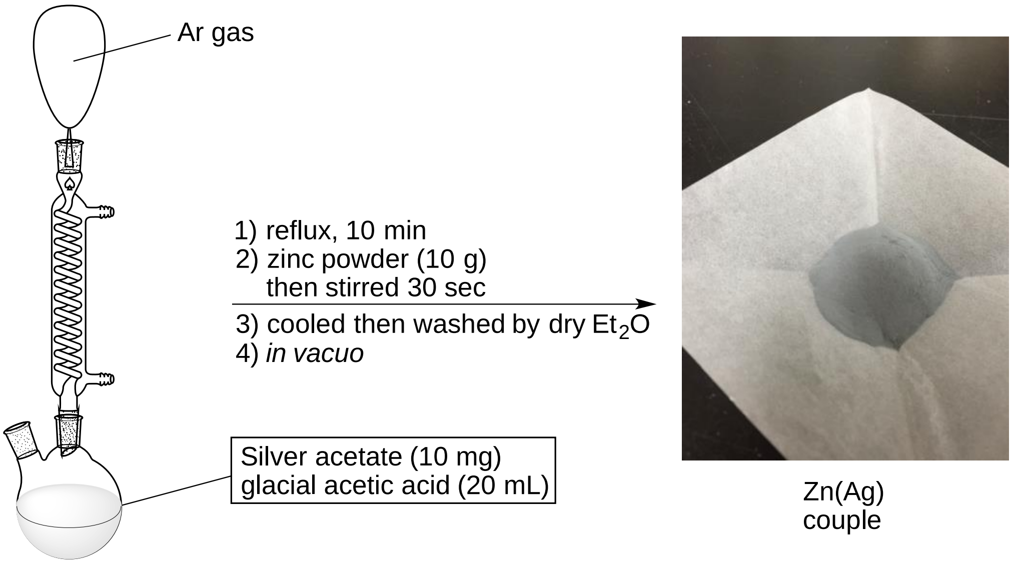

Figure S1: Preparation of Zn-Ag couple

Reference

S1 Watanabe, Y.; Konno, T. *J. Fluorine Chem.* **2015,** *174,* 102–107. doi:10.1016/j.jfluchem.2014.12.008

3. Copies of ^{1}H, ^{13}C, and ^{19}F NMR spectra for new compounds

^{1}H NMR Spectrum of 3,3,4,4-tetrafluoro-4-(4-trifluoromethylphenyl)but-1-ene (**4e**)

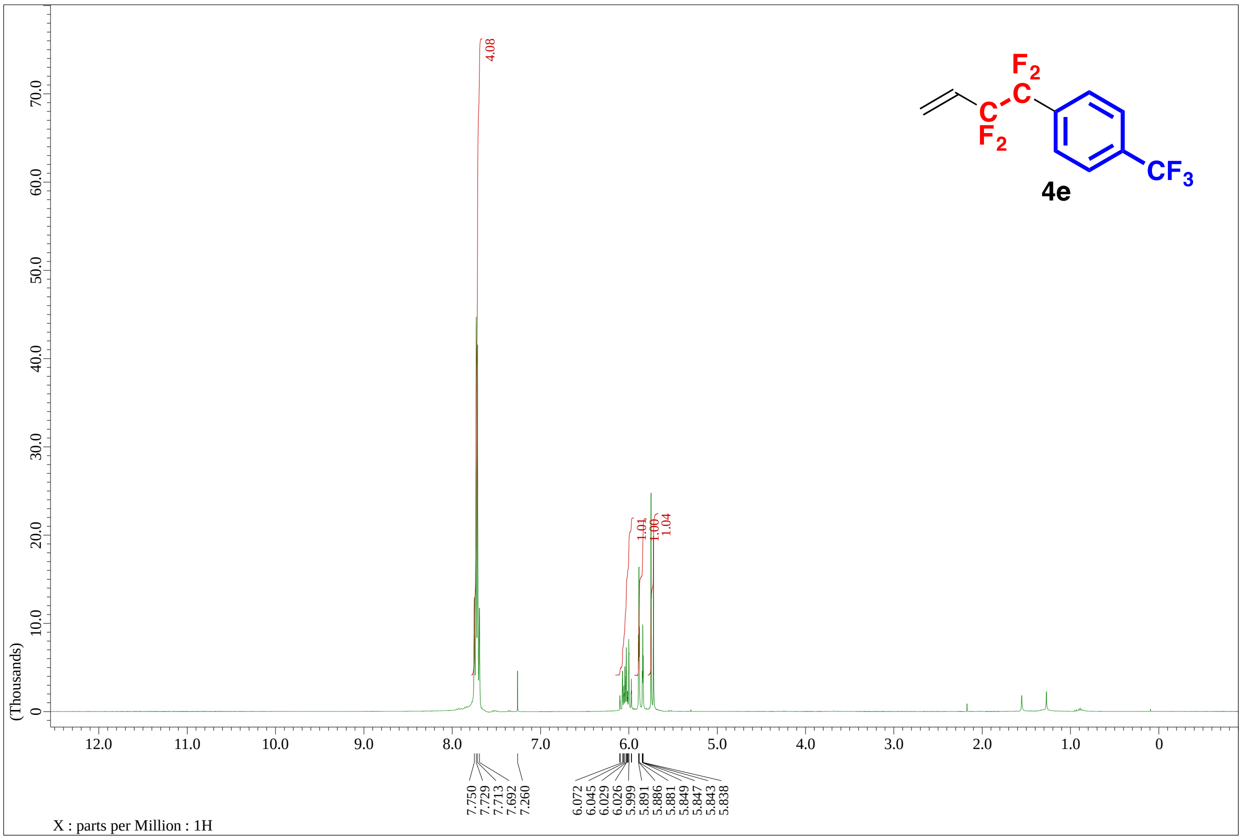

^{13}C NMR Spectrum of 3,3,4,4-tetrafluoro-4-(4-trifluoromethylphenyl)but-1-ene (**4e**)

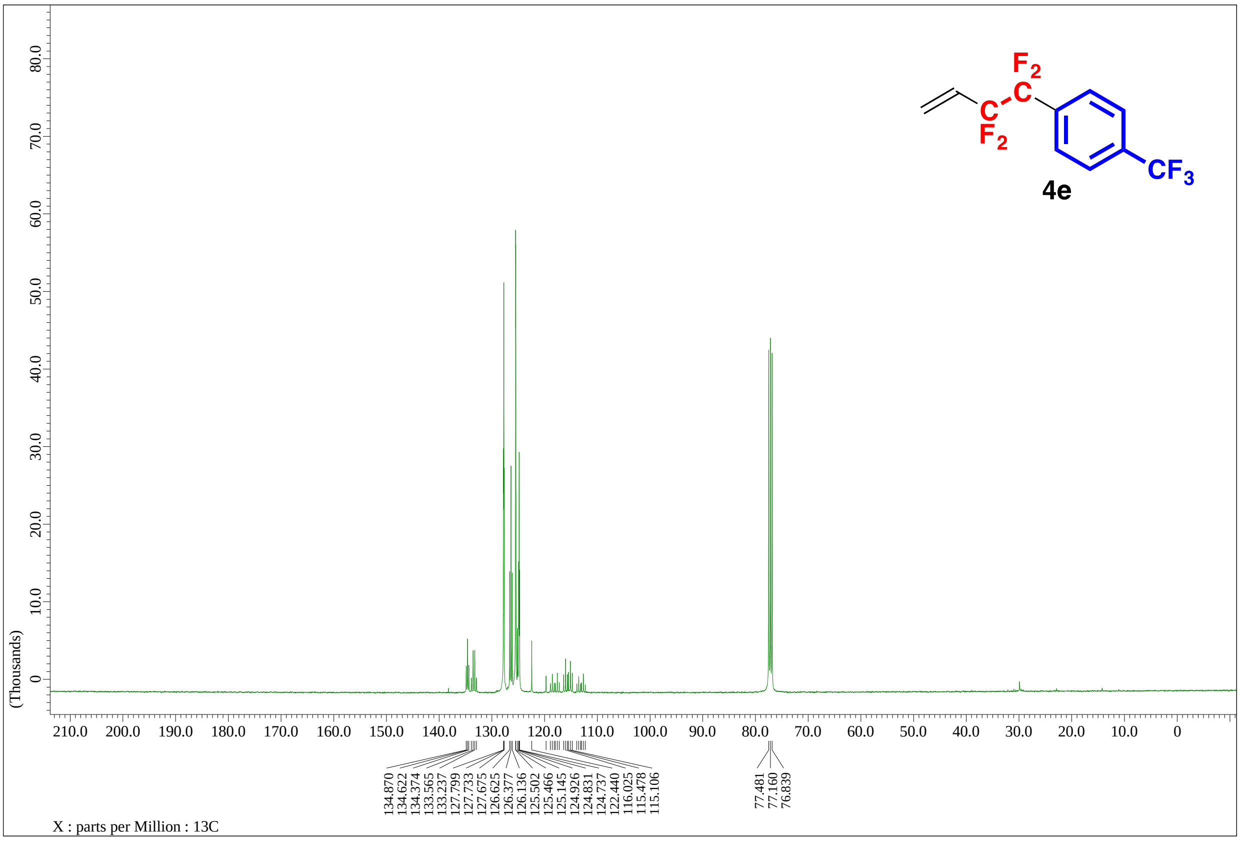

^{19}F NMR Spectrum of 3,3,4,4-tetrafluoro-4-(4-trifluoromethylphenyl)but-1-ene (**4e**)

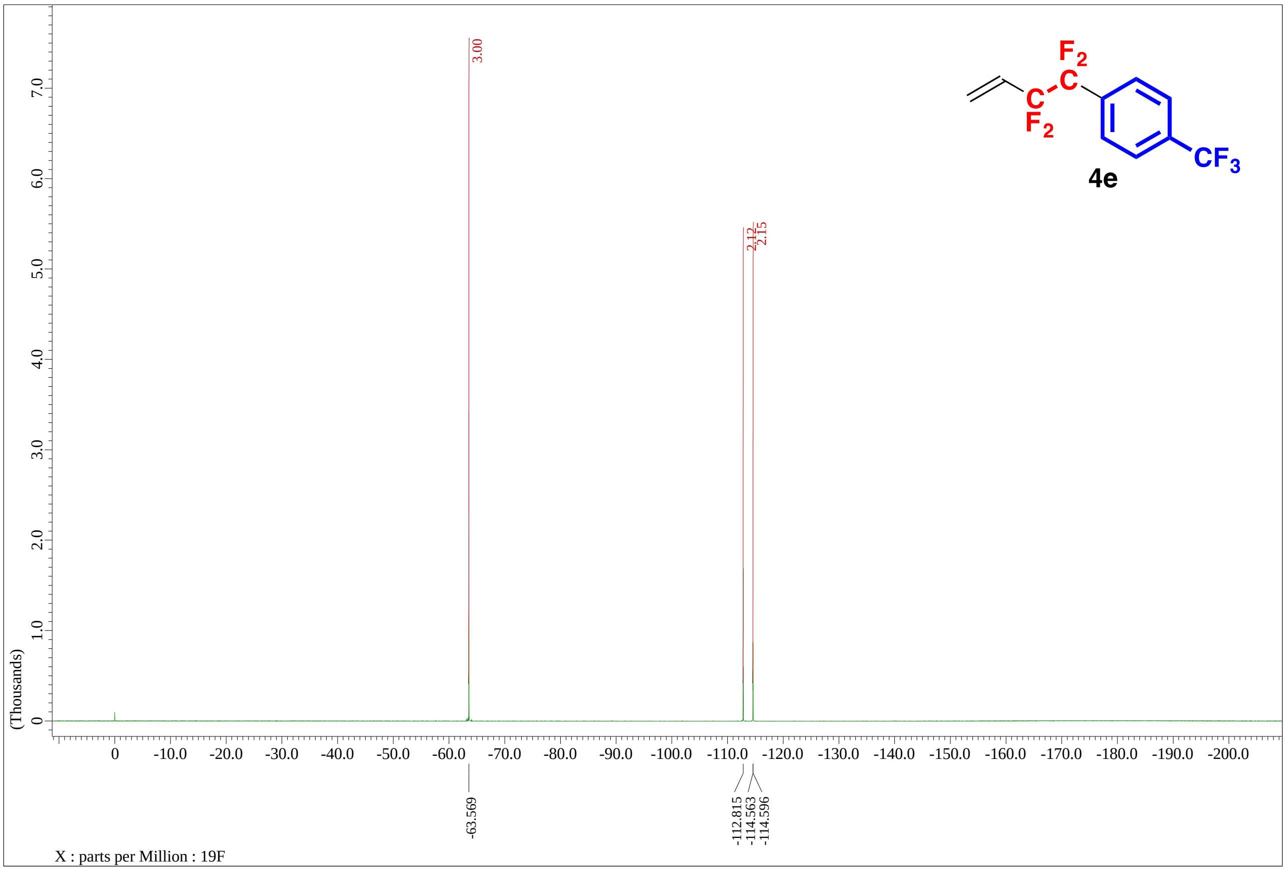

^{1}H NMR Spectrum of 4-(3-chlorophenyl)-3,3,4,4-tetrafluorobut-1-ene (**4h**)

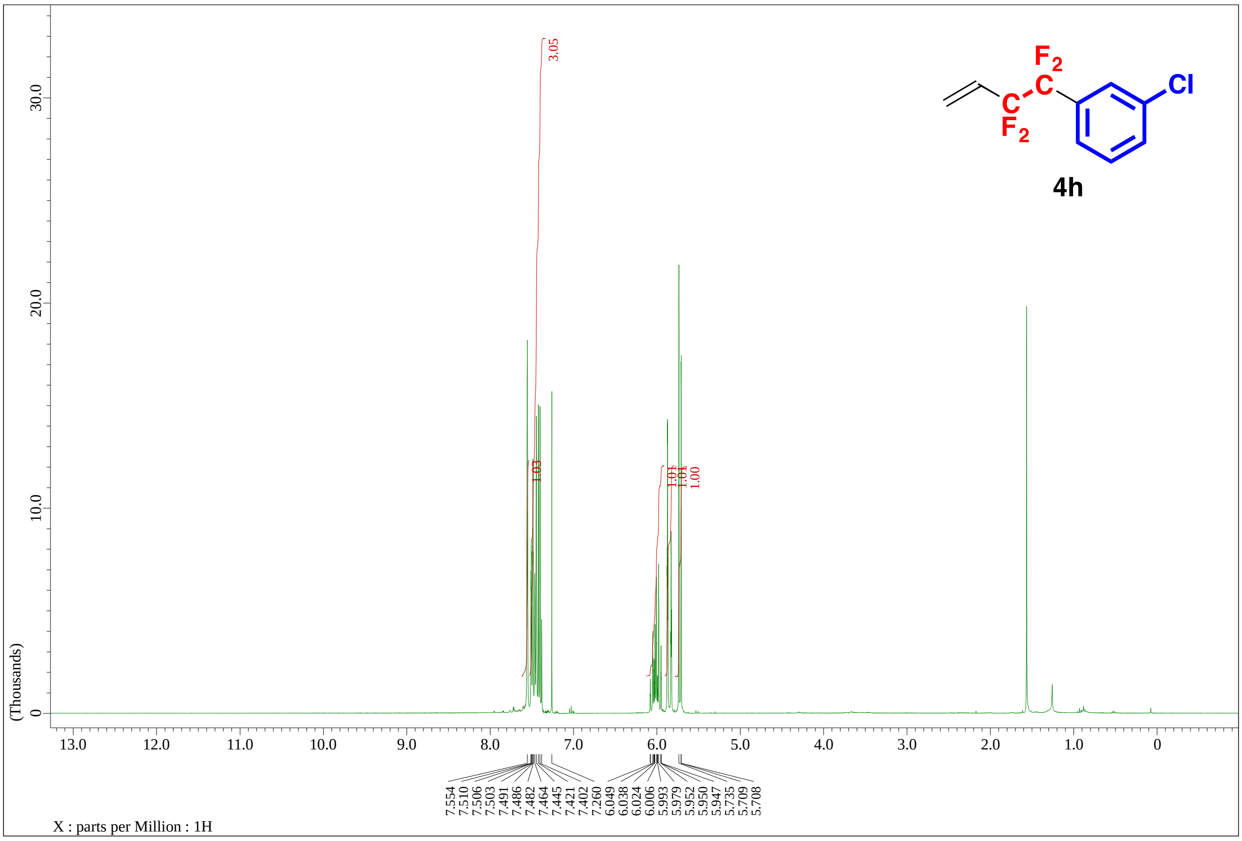

^{13}C NMR Spectrum of 4-(3-chlorophenyl)-3,3,4,4-tetrafluorobut-1-ene (**4h**)

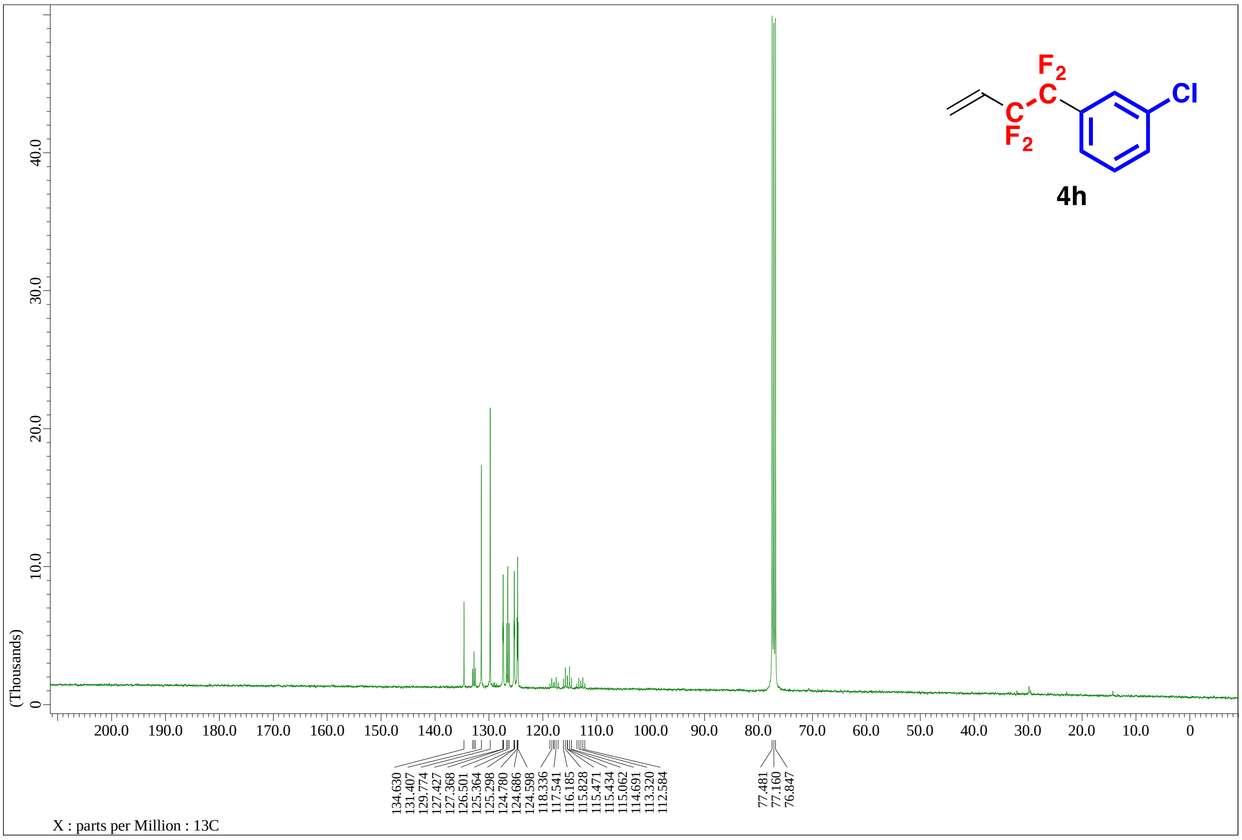

^{19}F NMR Spectrum of 4-(3-chlorophenyl)-3,3,4,4-tetrafluorobut-1-ene (**4h**)

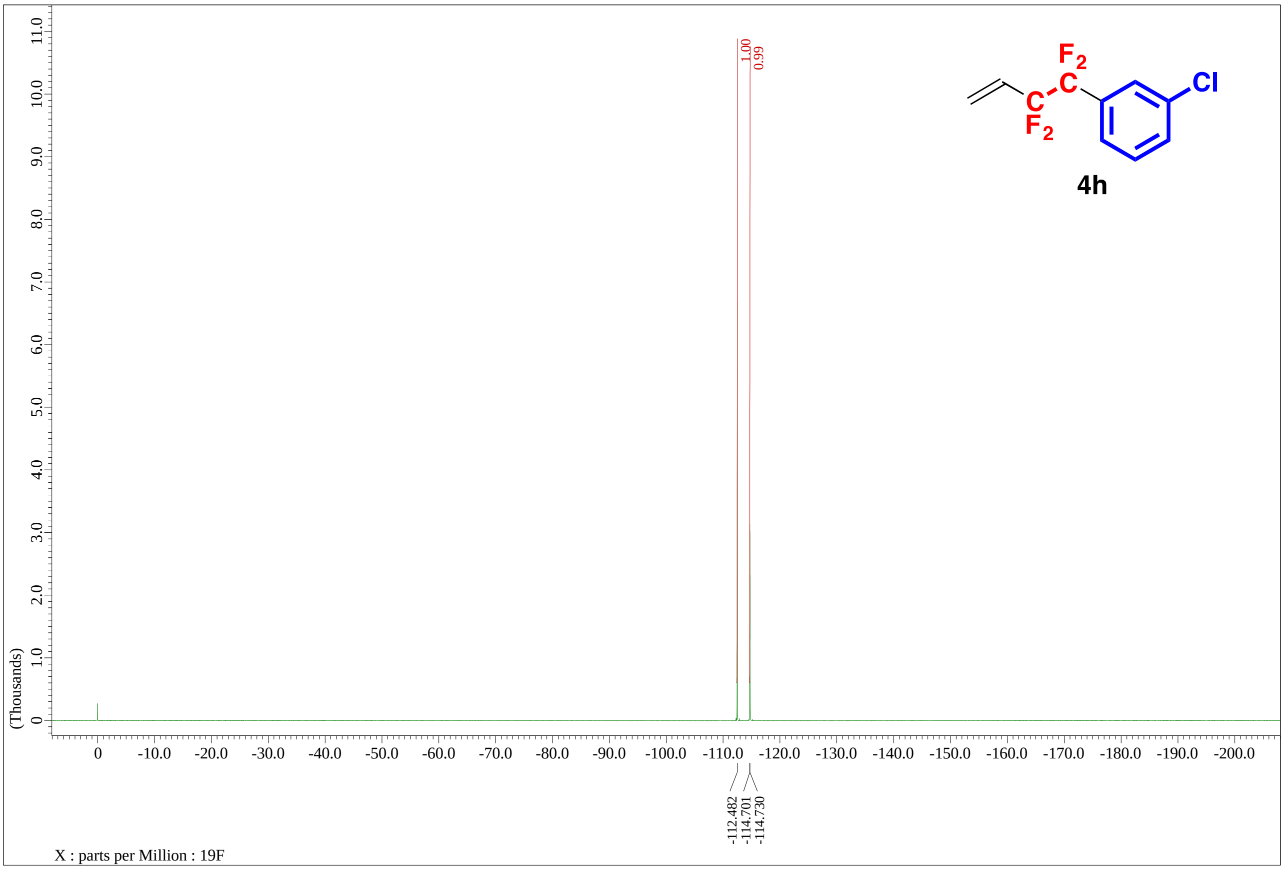

^{1}H NMR Spectrum of 4-(2-chlorophenyl)-3,3,4,4-tetrafluorobut-1-ene (**4i**)

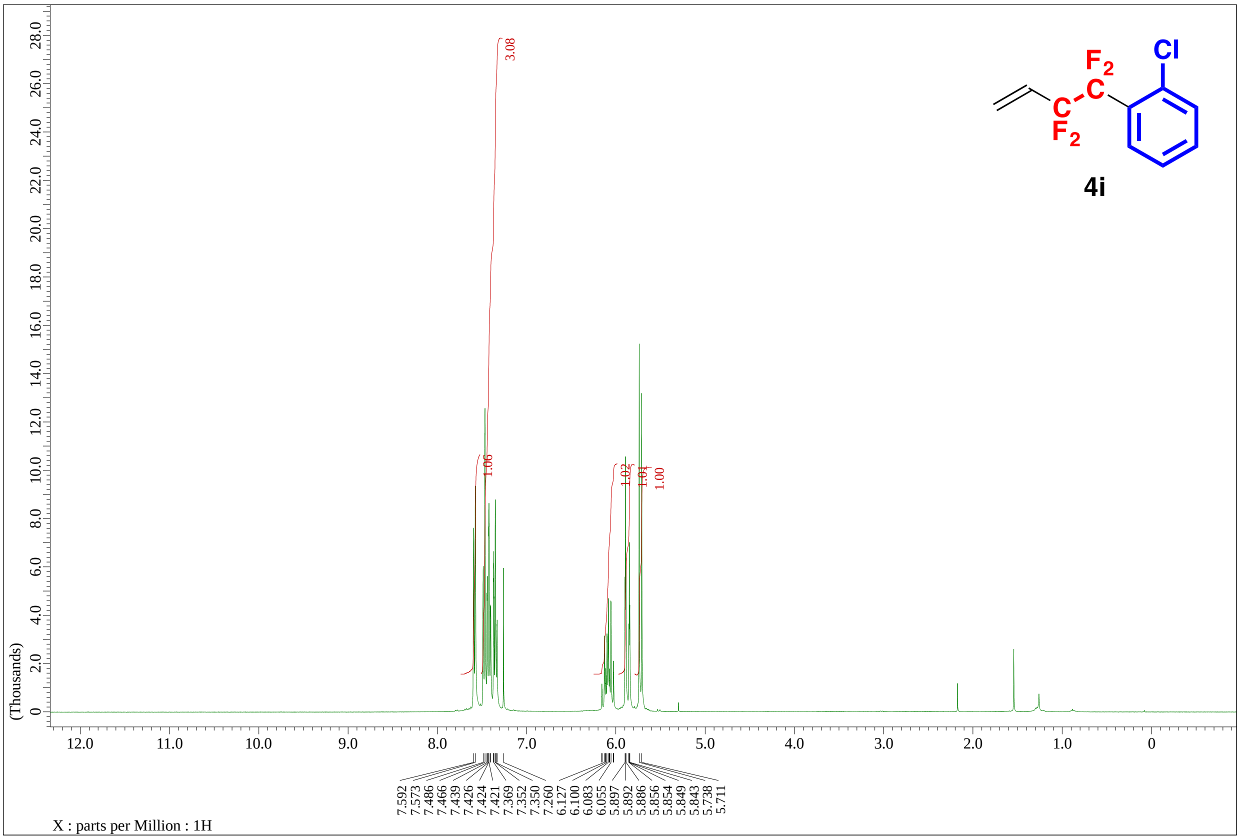

^{13}C NMR Spectrum of 4-(2-chlorophenyl)-3,3,4,4-tetrafluorobut-1-ene (**4i**)

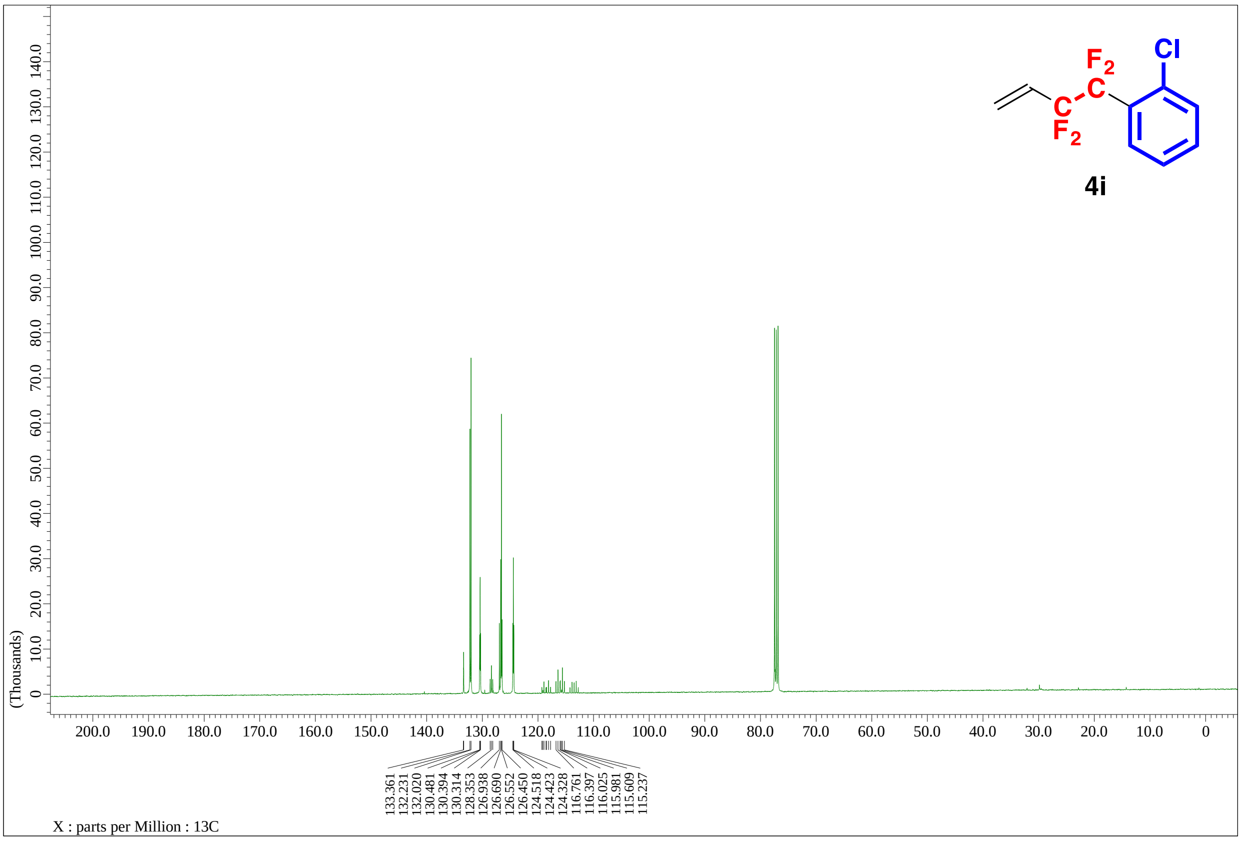

^{19}F NMR Spectrum of 4-(2-chlorophenyl)-3,3,4,4-tetrafluorobut-1-ene (**4i**)

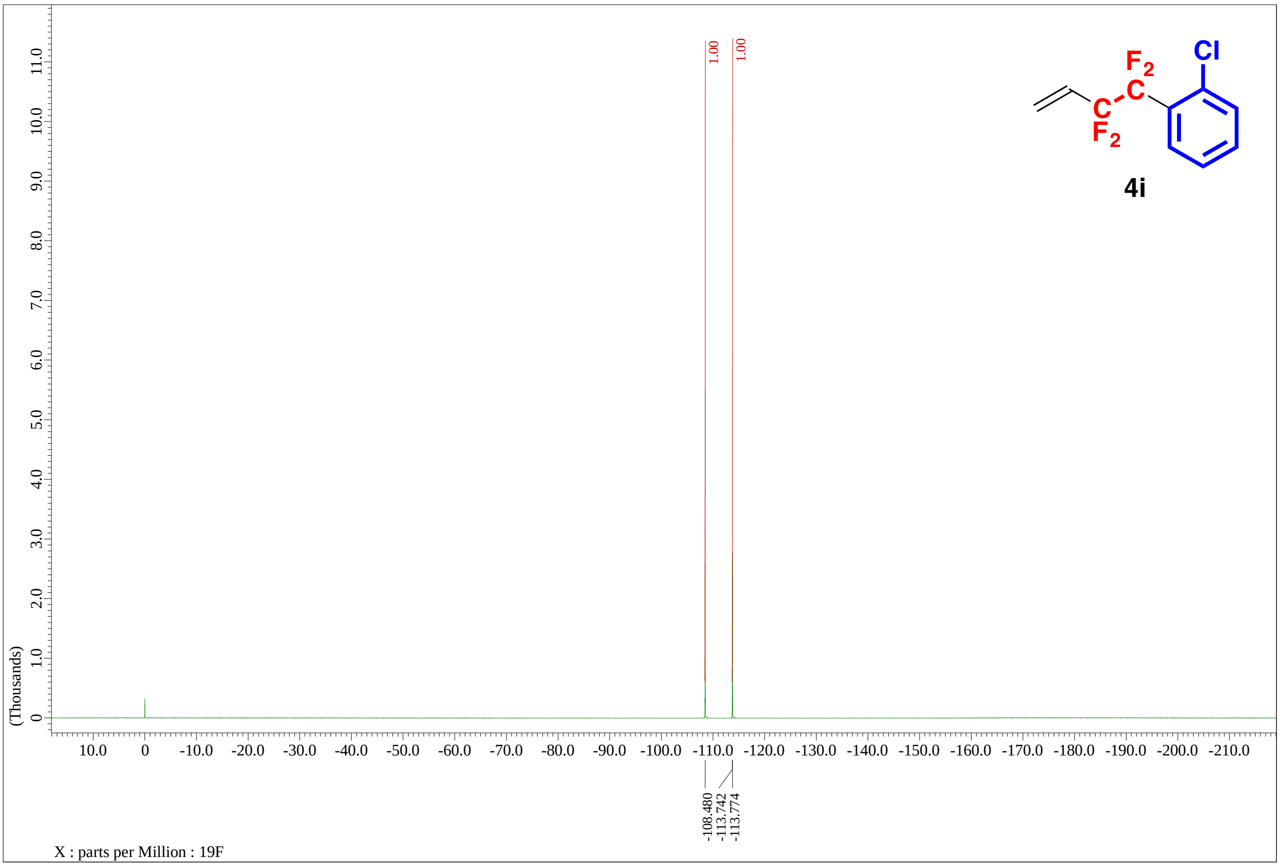

^{1}H NMR Spectrum of ethyl 2-(1,1,2,2-tetrafluorobut-3-en-1-yl)benzoate (**4j**)

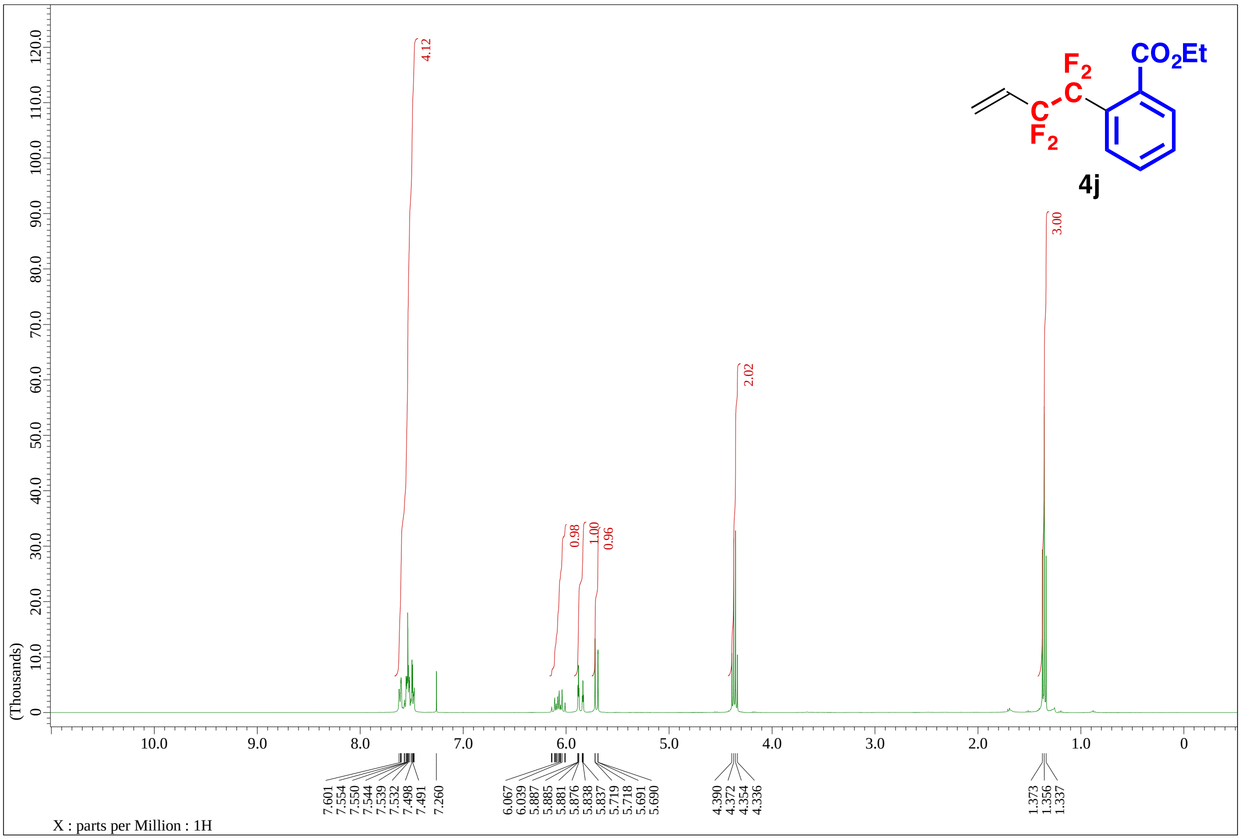

^{13}C NMR Spectrum of ethyl 2-(1,1,2,2-tetrafluorobut-3-en-1-yl)benzoate (**4j**)

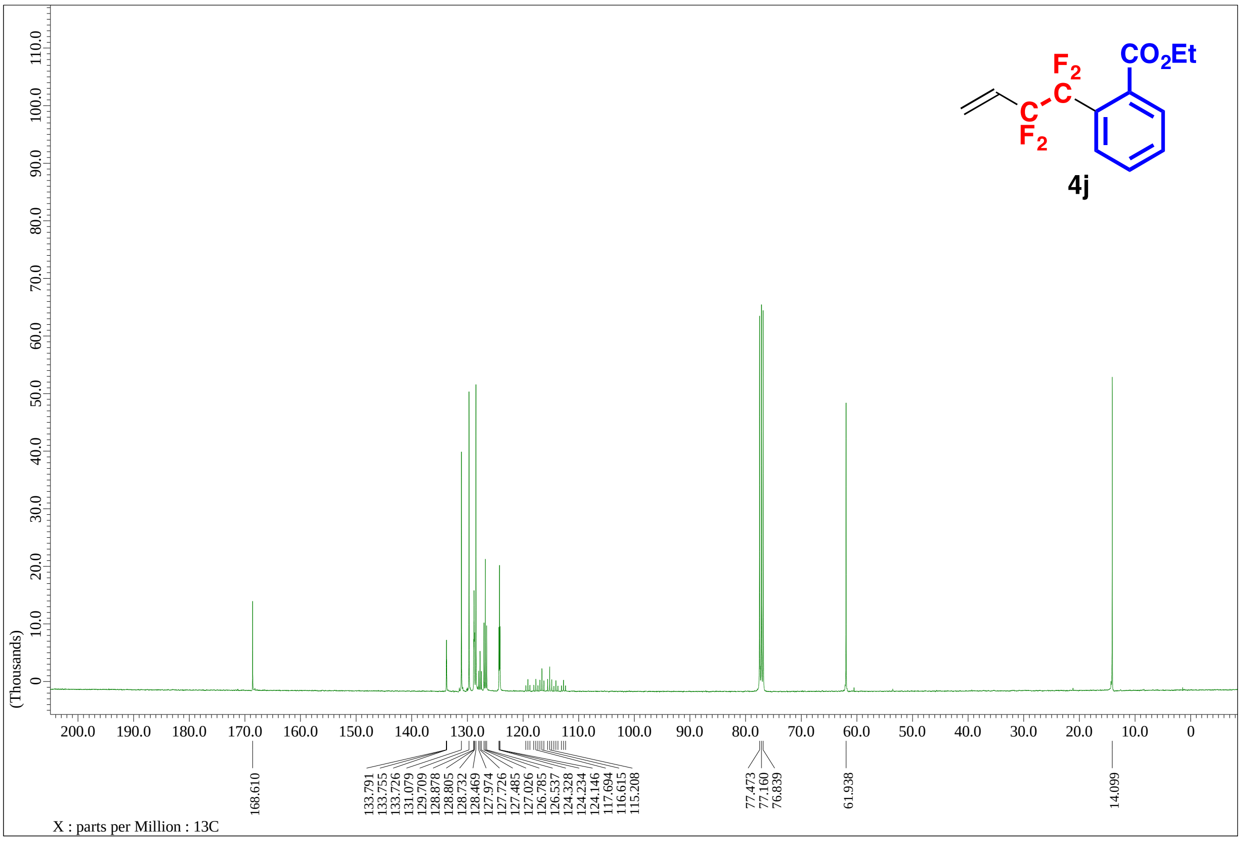

^{19}F NMR Spectrum of ethyl 2-(1,1,2,2-tetrafluorobut-3-en-1-yl)benzoate (**4j**)

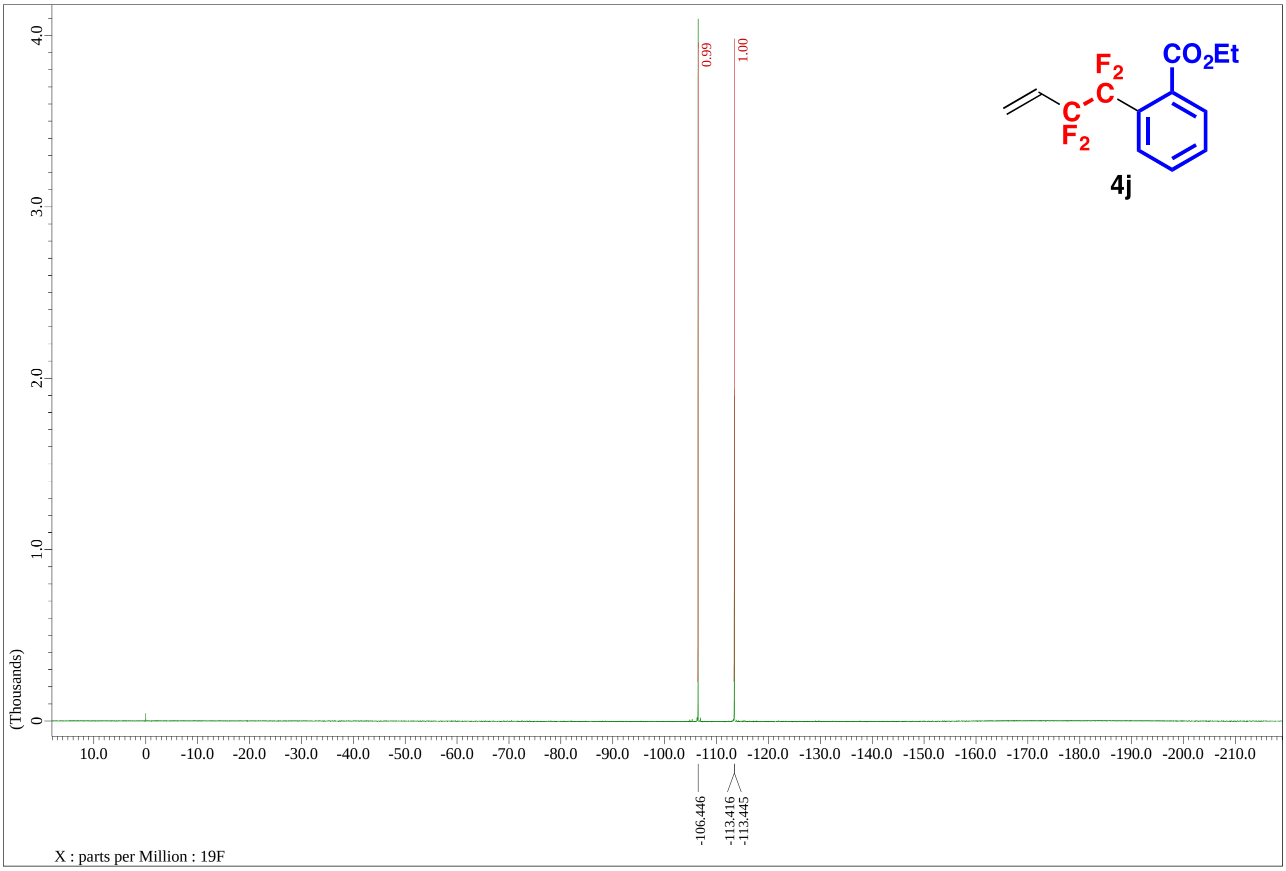

^{1}H NMR Spectrum of 3,3,4,4-tetrafluoro-4-(2-formylphenyl)but-1-ene (**4k**)

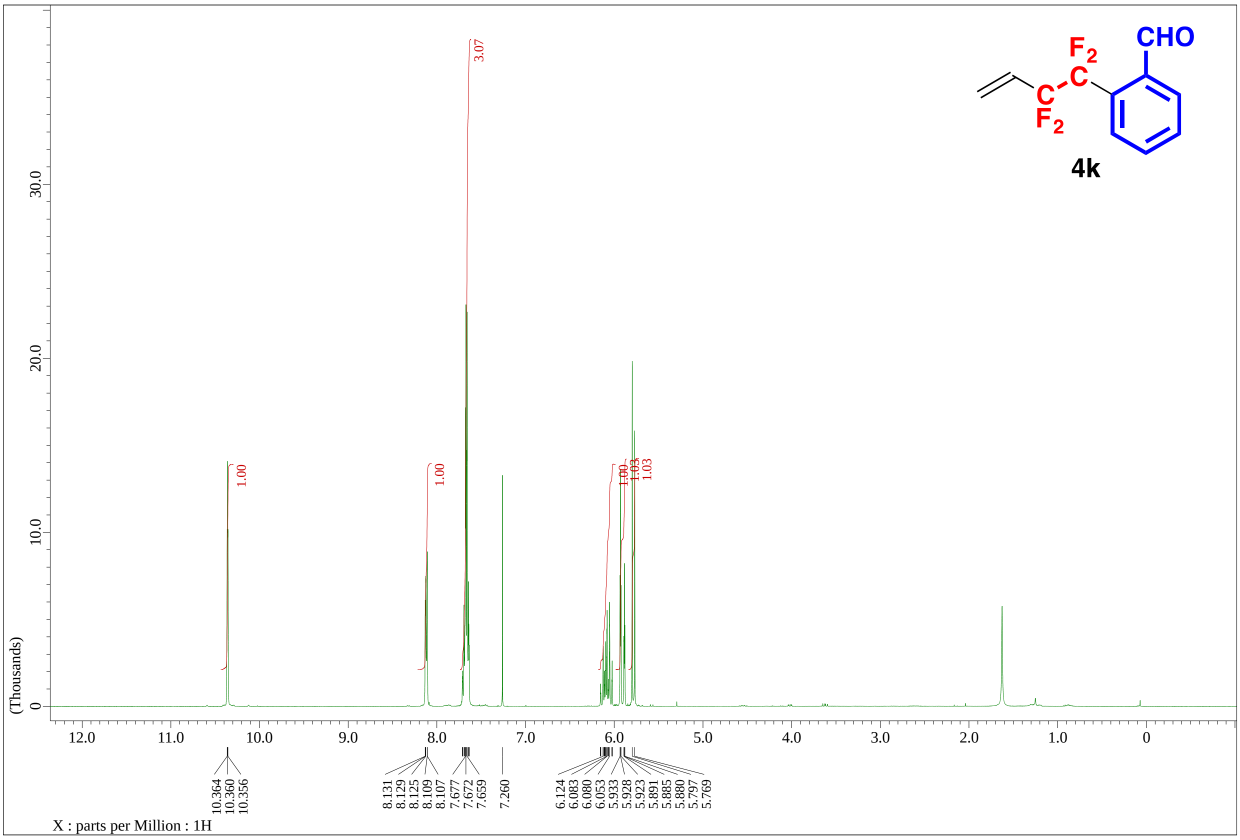

^{13}C NMR Spectrum of 3,3,4,4-tetrafluoro-4-(2-formylphenyl)but-1-ene (**4k**)

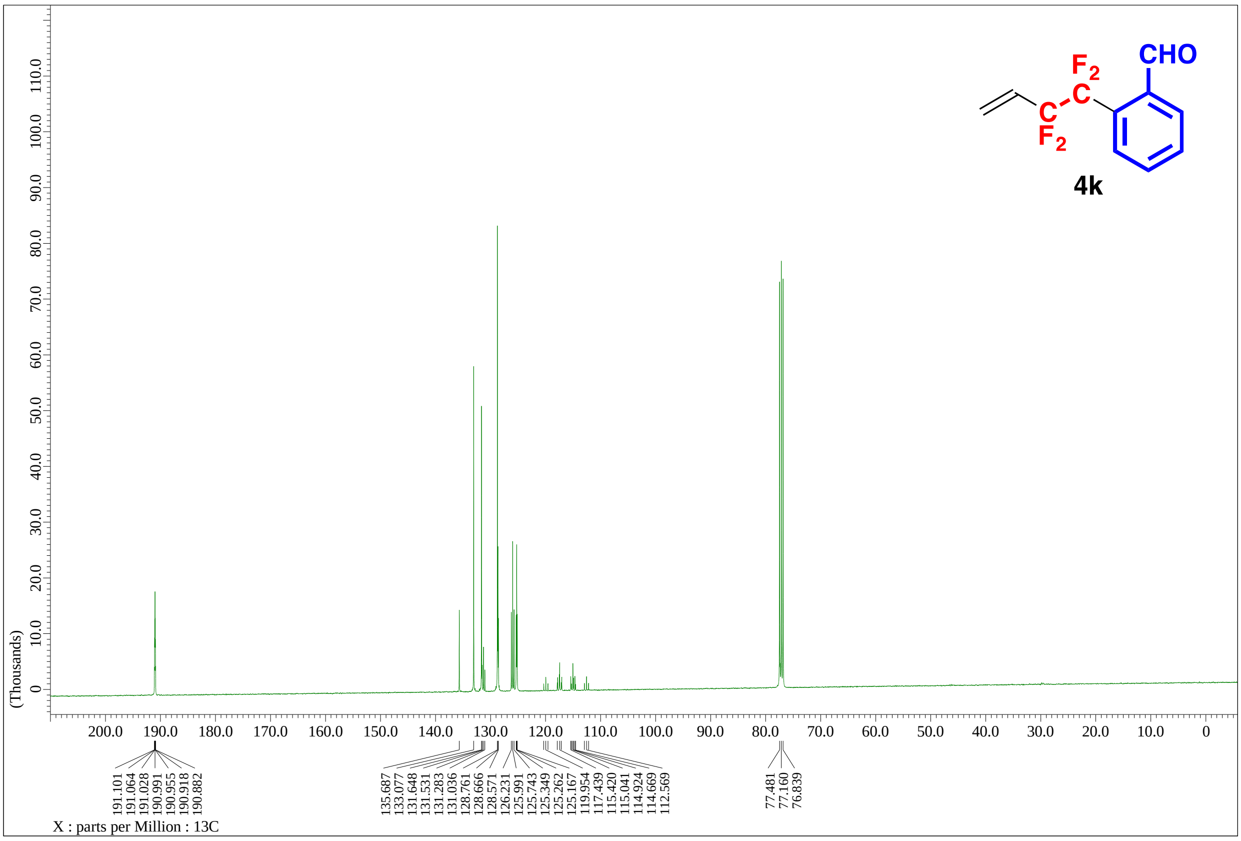

^{19}F NMR Spectrum of 3,3,4,4-tetrafluoro-4-(2-formylphenyl)but-1-ene (**4k**)

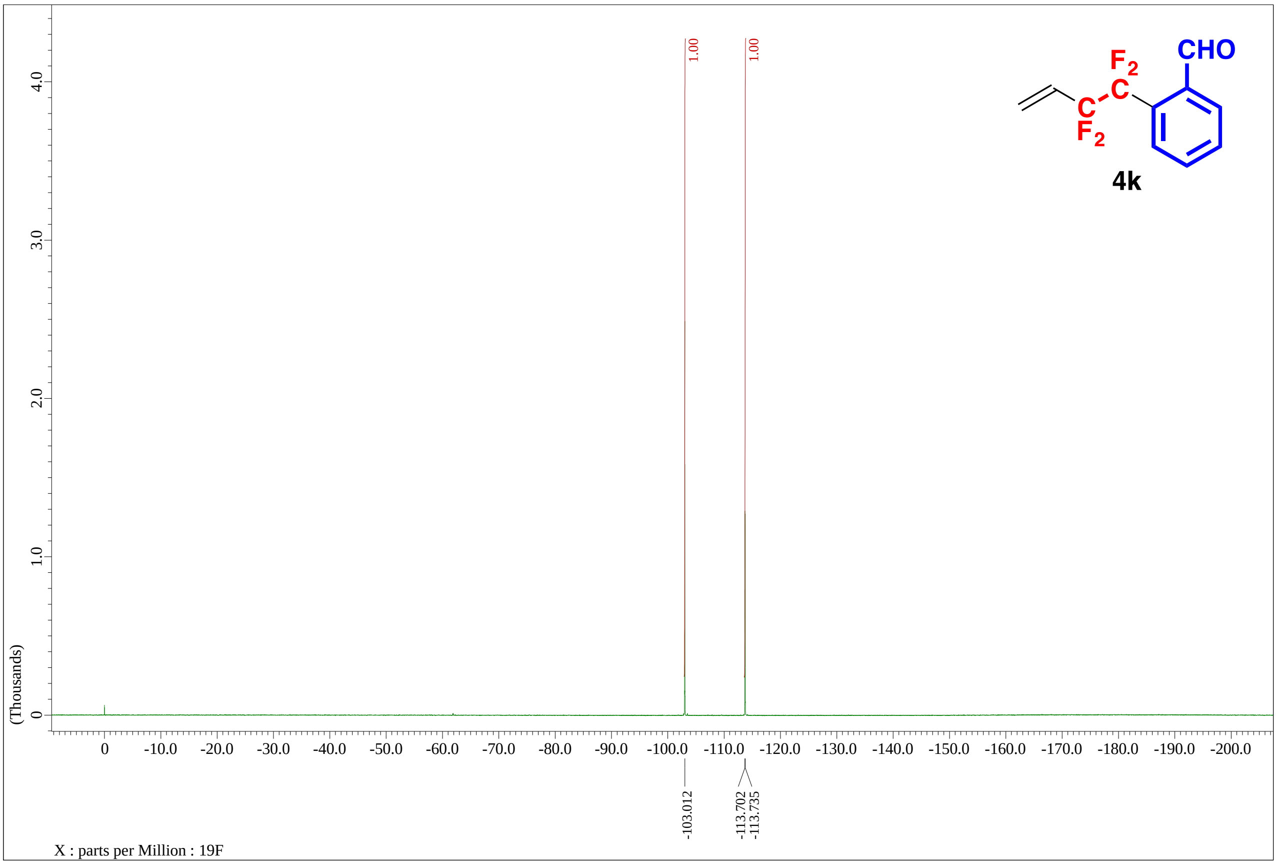

^{1}H NMR Spectrum of 3,3,4,4-tetrafluoro-4-(2-methoxymethylphenyl)but-1-ene (**4m**)

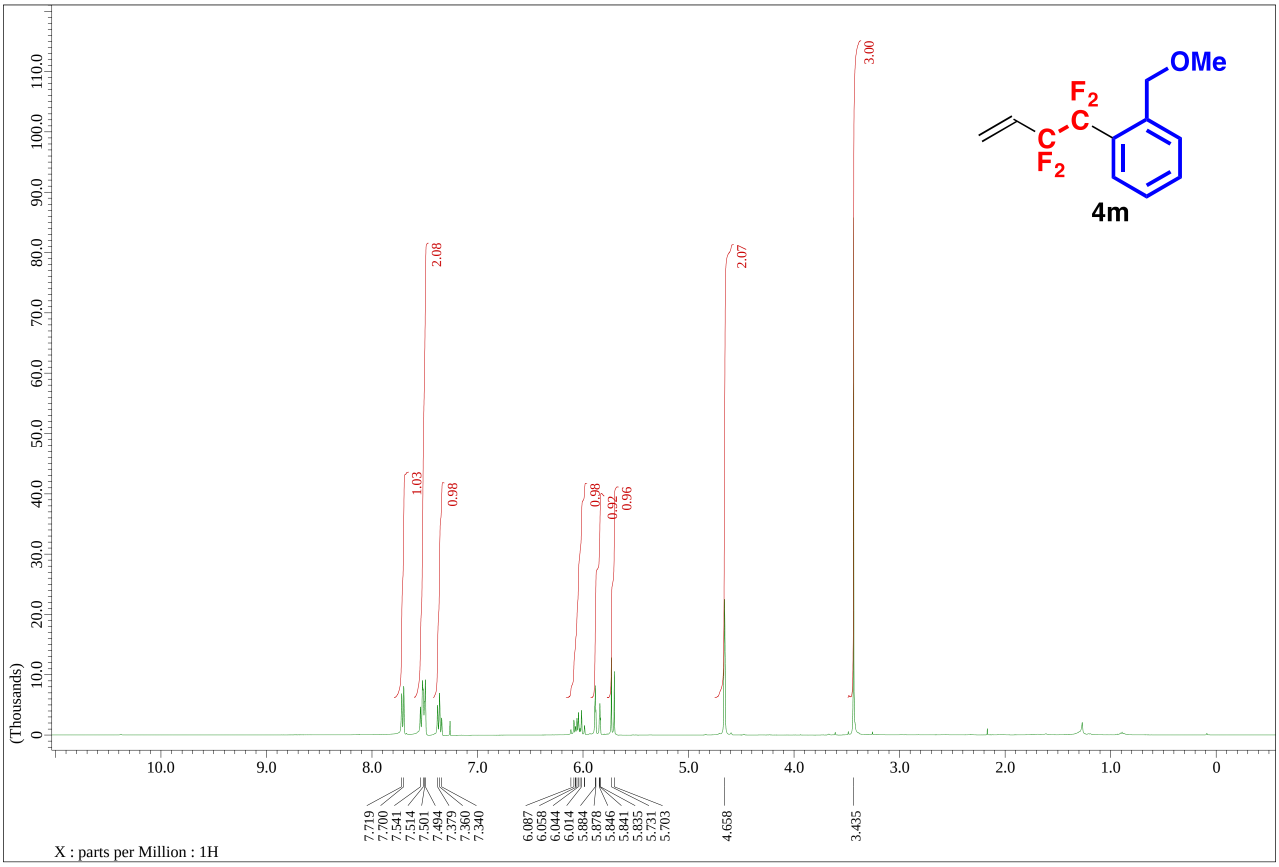

^{13}C NMR Spectrum of 3,3,4,4-tetrafluoro-4-(2-methoxymethylphenyl)but-1-ene (**4m**)

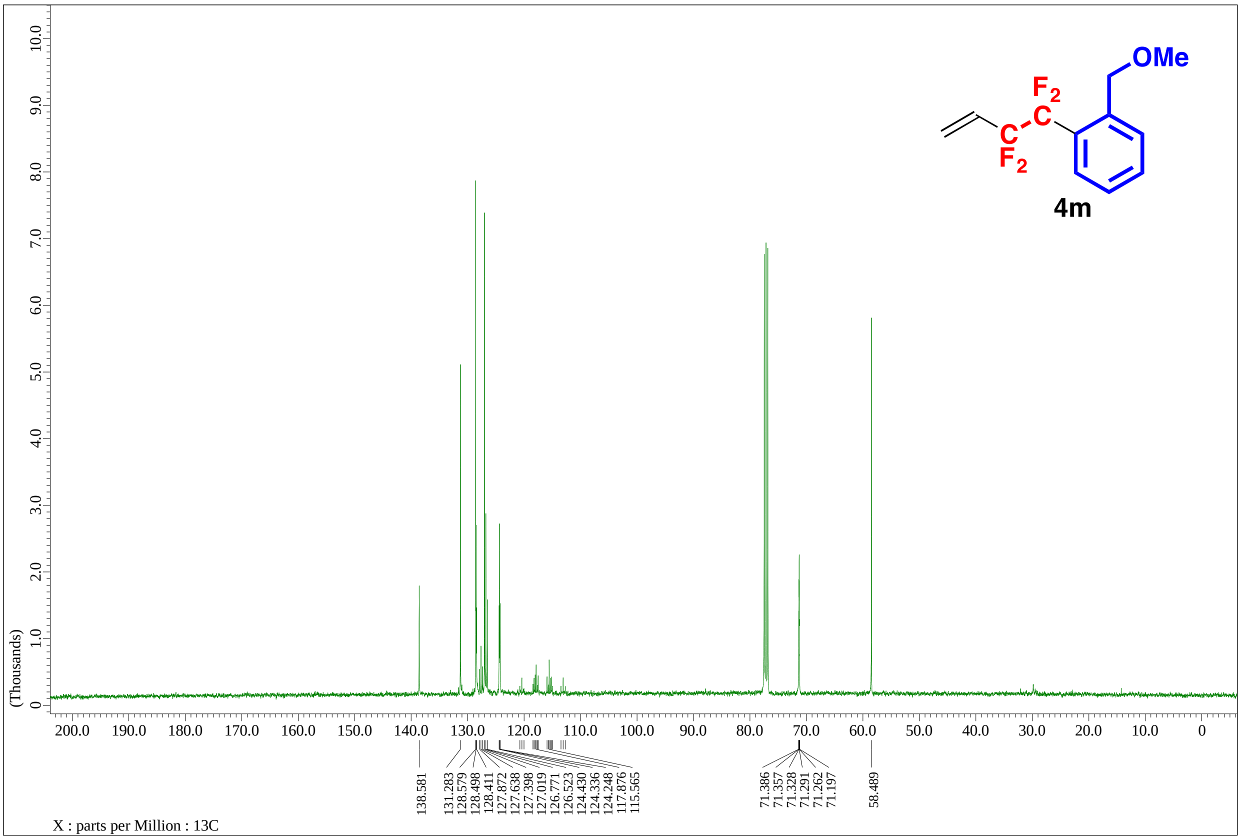

^{19}F NMR Spectrum of 3,3,4,4-tetrafluoro-4-(2-methoxymethylphenyl)but-1-ene (**4m**)

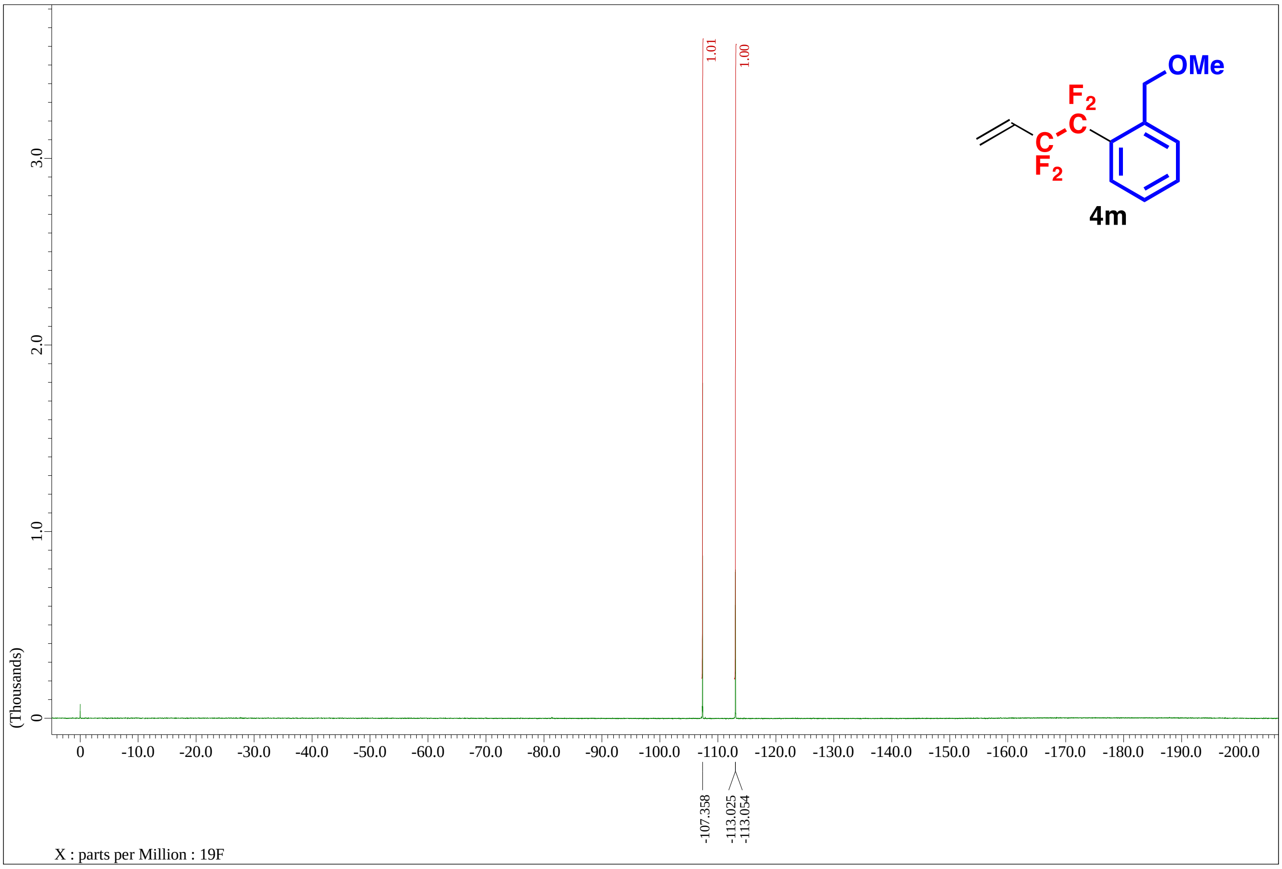

^{1}H NMR Spectrum of 3,3,4,4-tetrafluoro-4-(2-nitrophenyl)but-1-ene (**4n**)

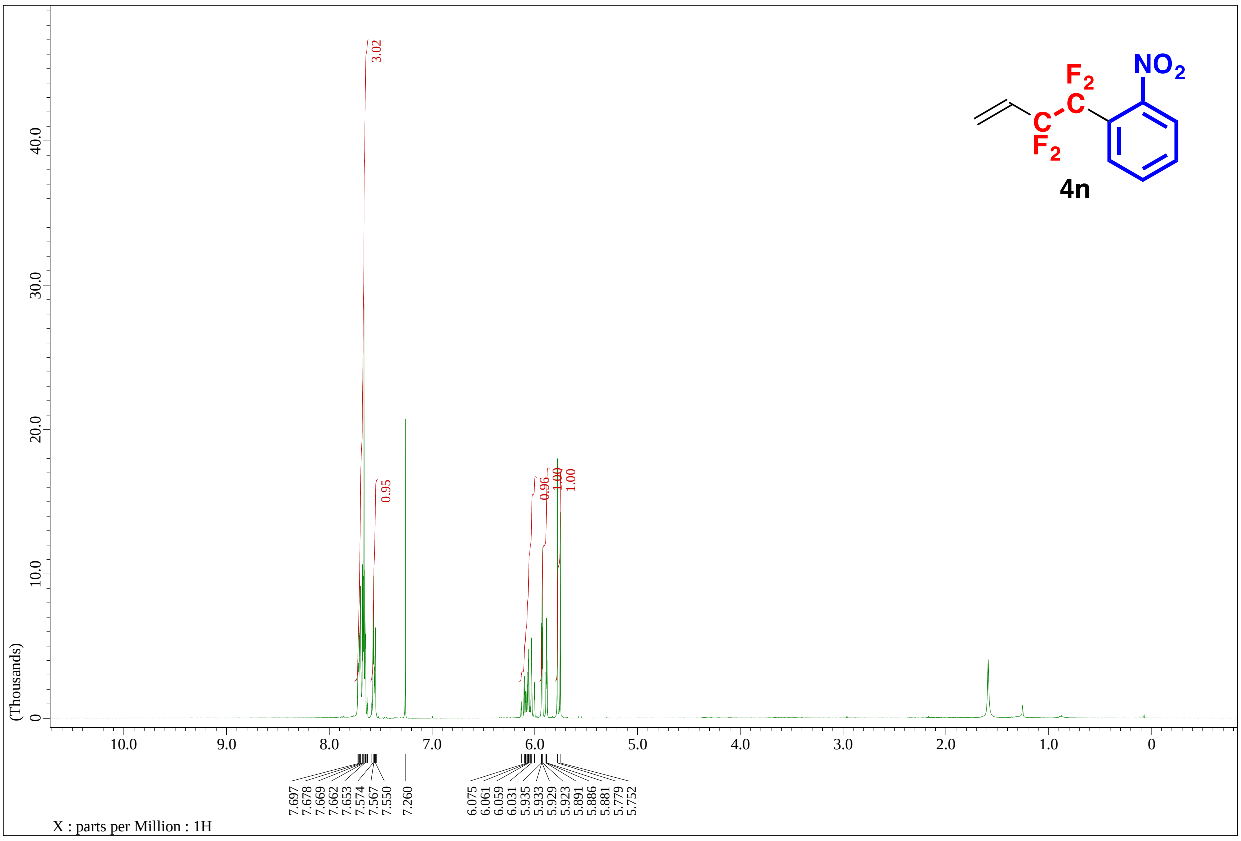

^{13}C NMR Spectrum of 3,3,4,4-tetrafluoro-4-(2-nitrophenyl)but-1-ene (**4n**)

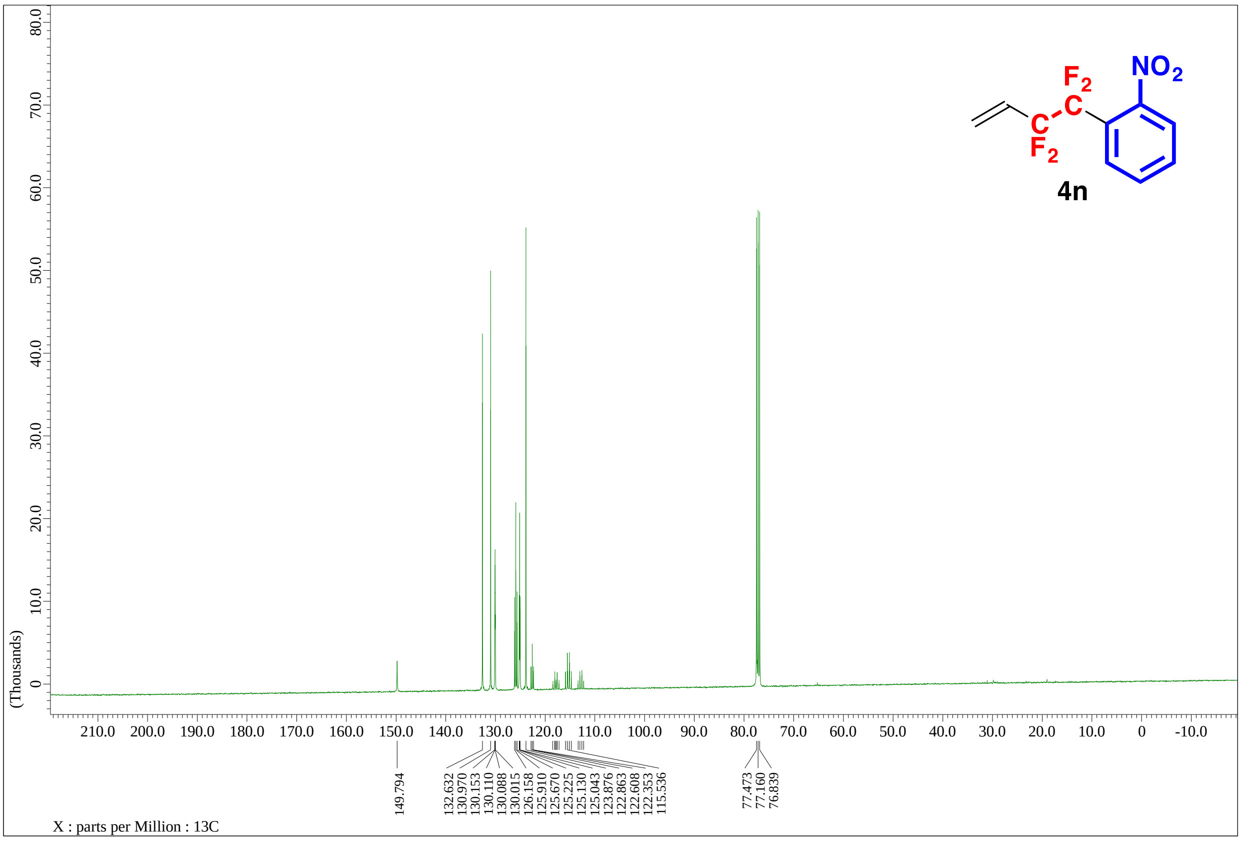

^{19}F NMR Spectrum of 3,3,4,4-tetrafluoro-4-(2-nitrophenyl)but-1-ene (**4n**)

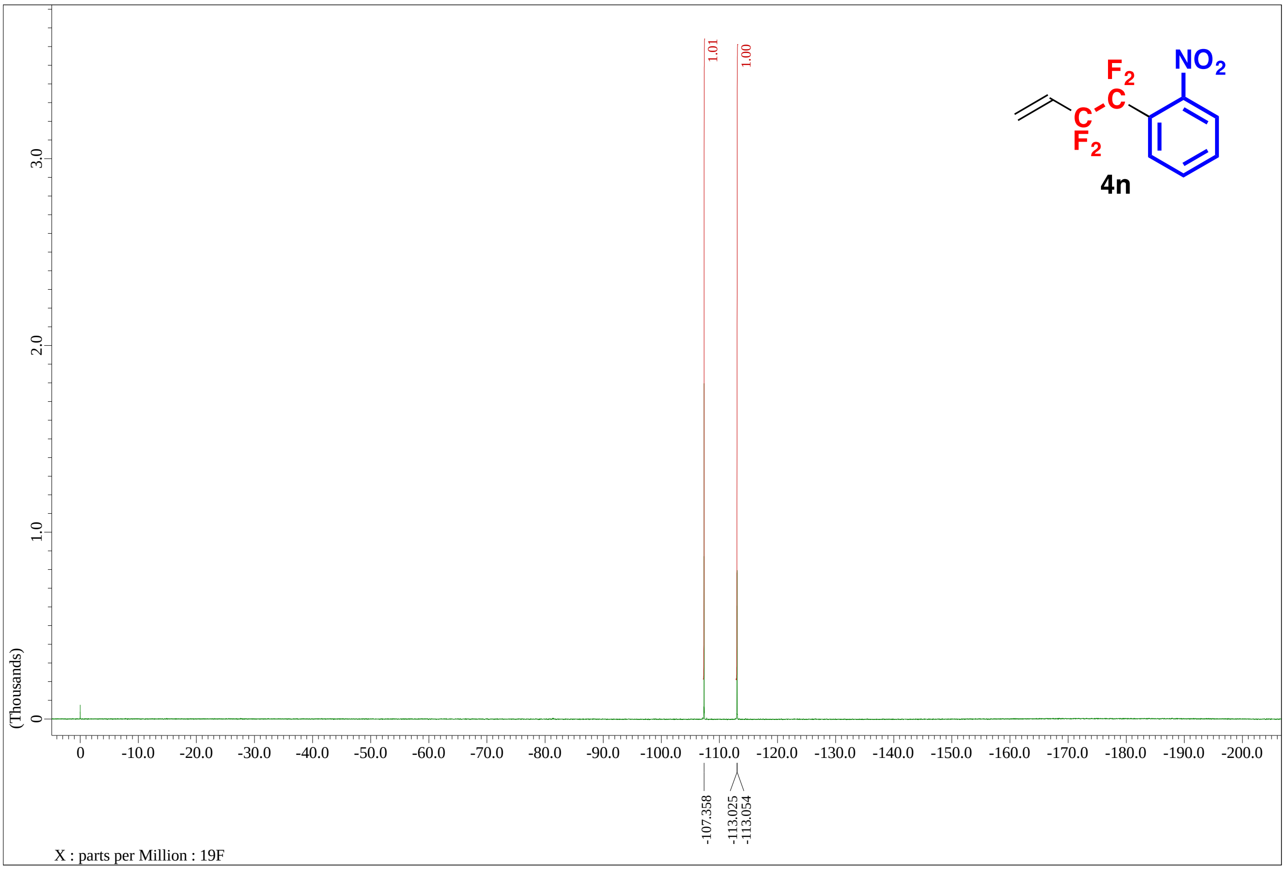

^{1}H NMR Spectrum of 4-(4-fluoro-2-nitrophenyl)-3,3,4,4-tetrafluorobut-1-ene (**4o**)

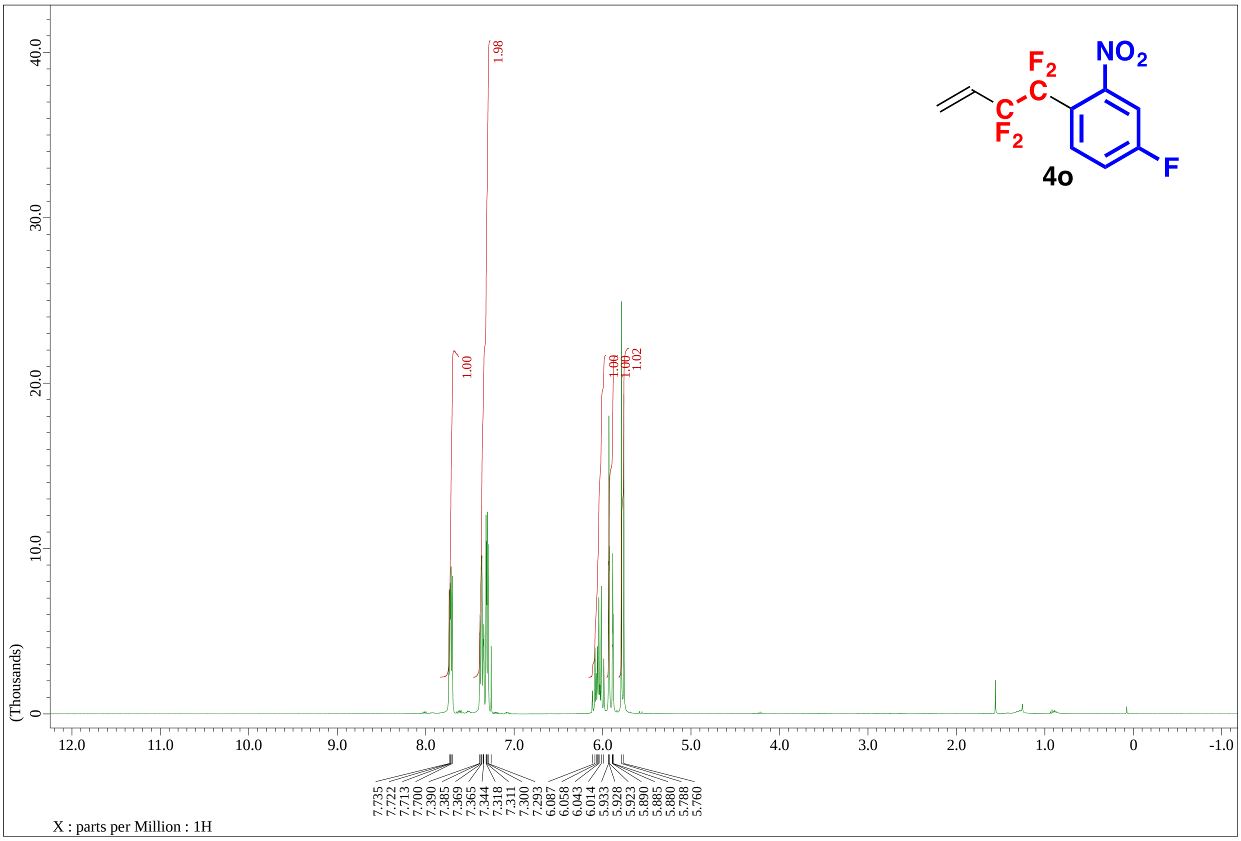

^{13}C NMR Spectrum of 4-(4-fluoro-2-nitrophenyl)-3,3,4,4-tetrafluorobut-1-ene (**4o**)

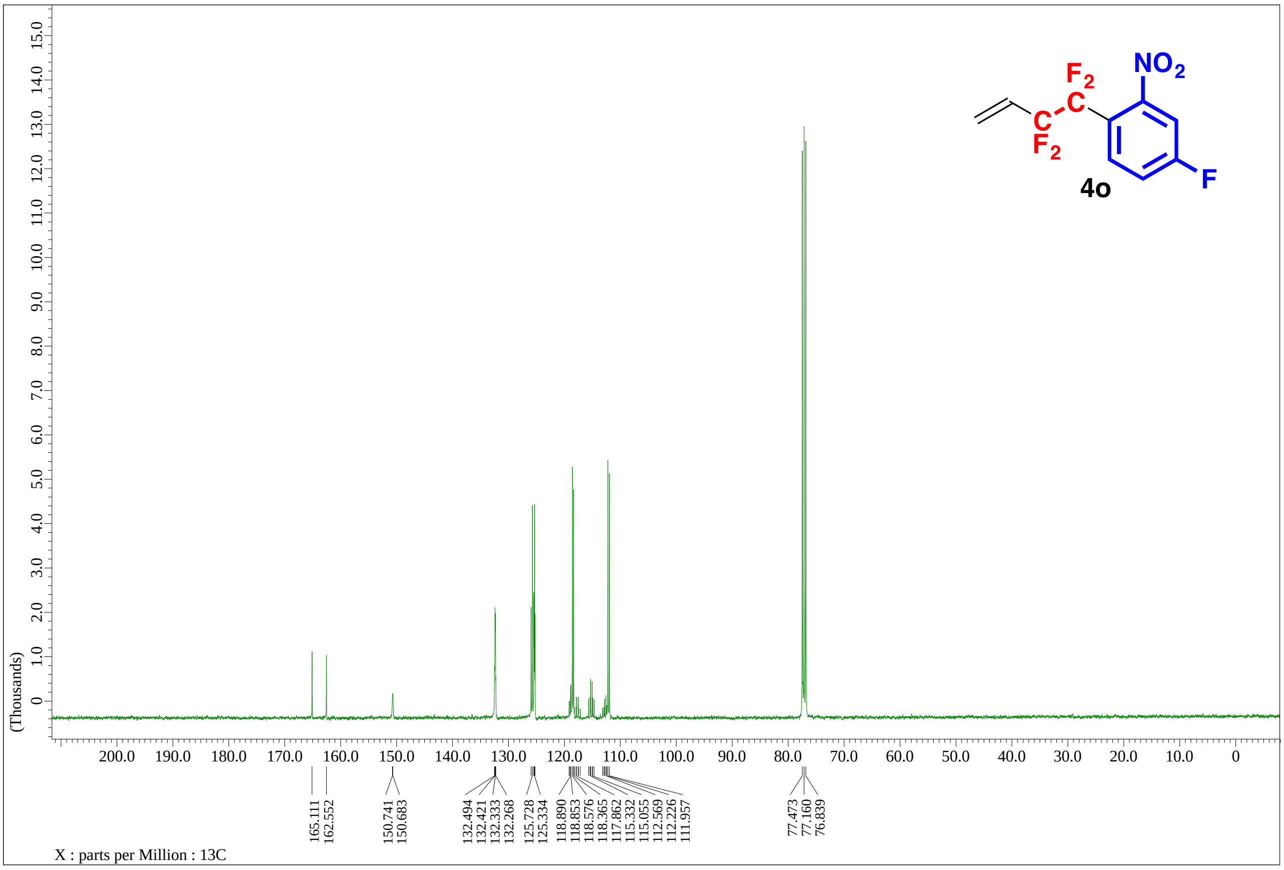

^{19}F NMR Spectrum of 4-(4-fluoro-2-nitrophenyl)-3,3,4,4-tetrafluorobut-1-ene (**4o**)

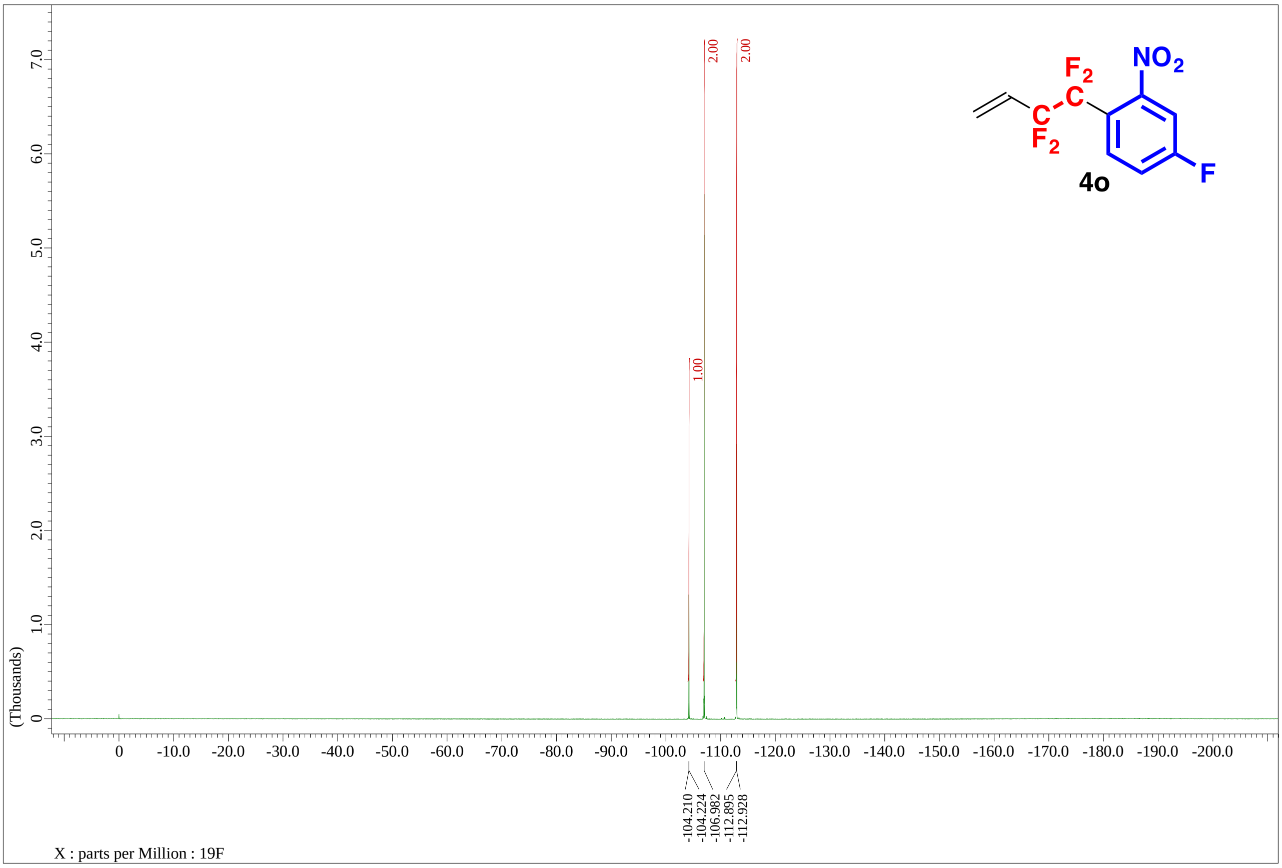

^{1}H NMR Spectrum of 3,3,4,4-tetrafluoro-4-(4-methoxy-2-nitrophenyl)but-1-ene (**4p**)

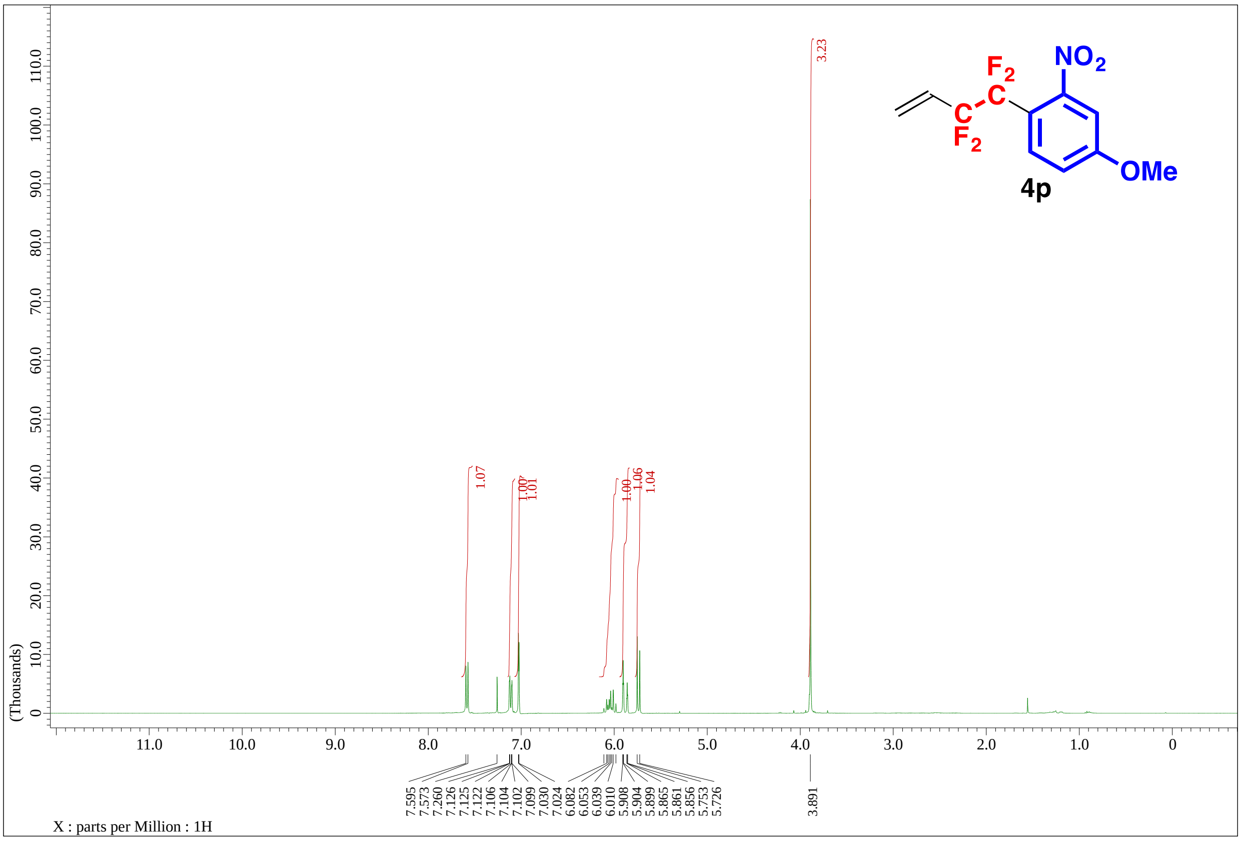

^{13}C NMR Spectrum of 3,3,4,4-tetrafluoro-4-(4-methoxy-2-nitrophenyl)but-1-ene (**4p**)

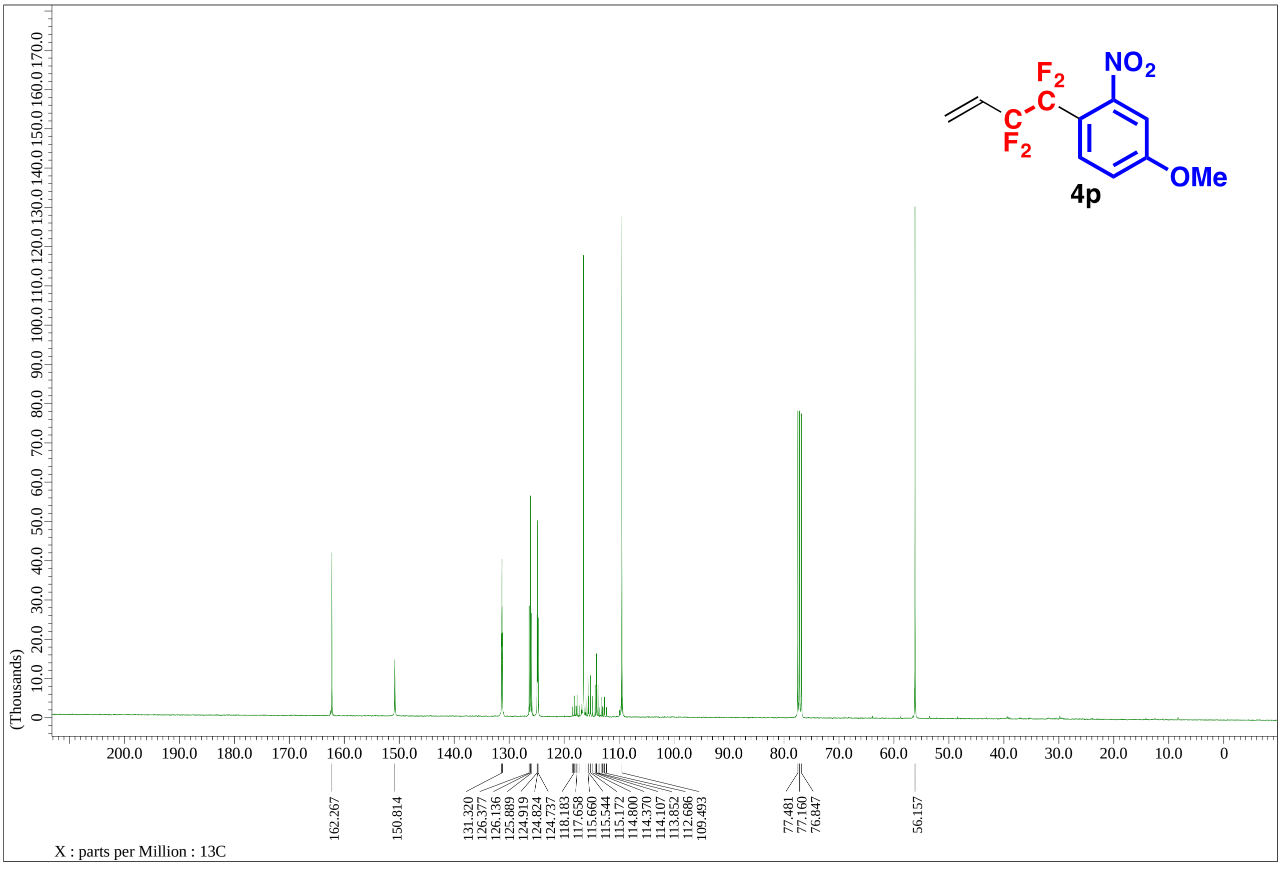

^{19}F NMR Spectrum of 3,3,4,4-tetrafluoro-4-(4-methoxy-2-nitrophenyl)but-1-ene (**4p**)

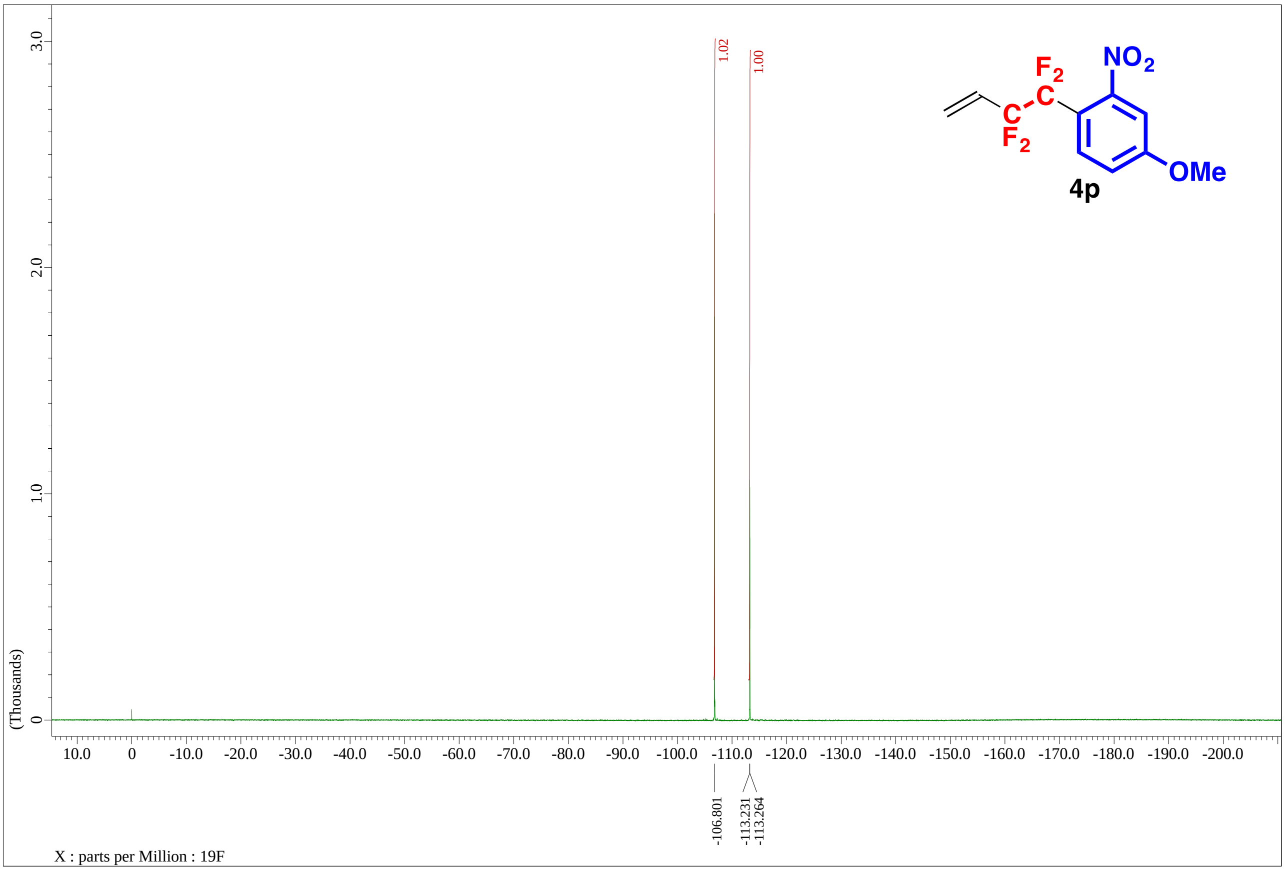

^{1}H NMR Spectrum of 3,3,4,4-tetrafluoro-4-(5-methoxy-2-nitrophenyl)but-1-ene (**4q**)

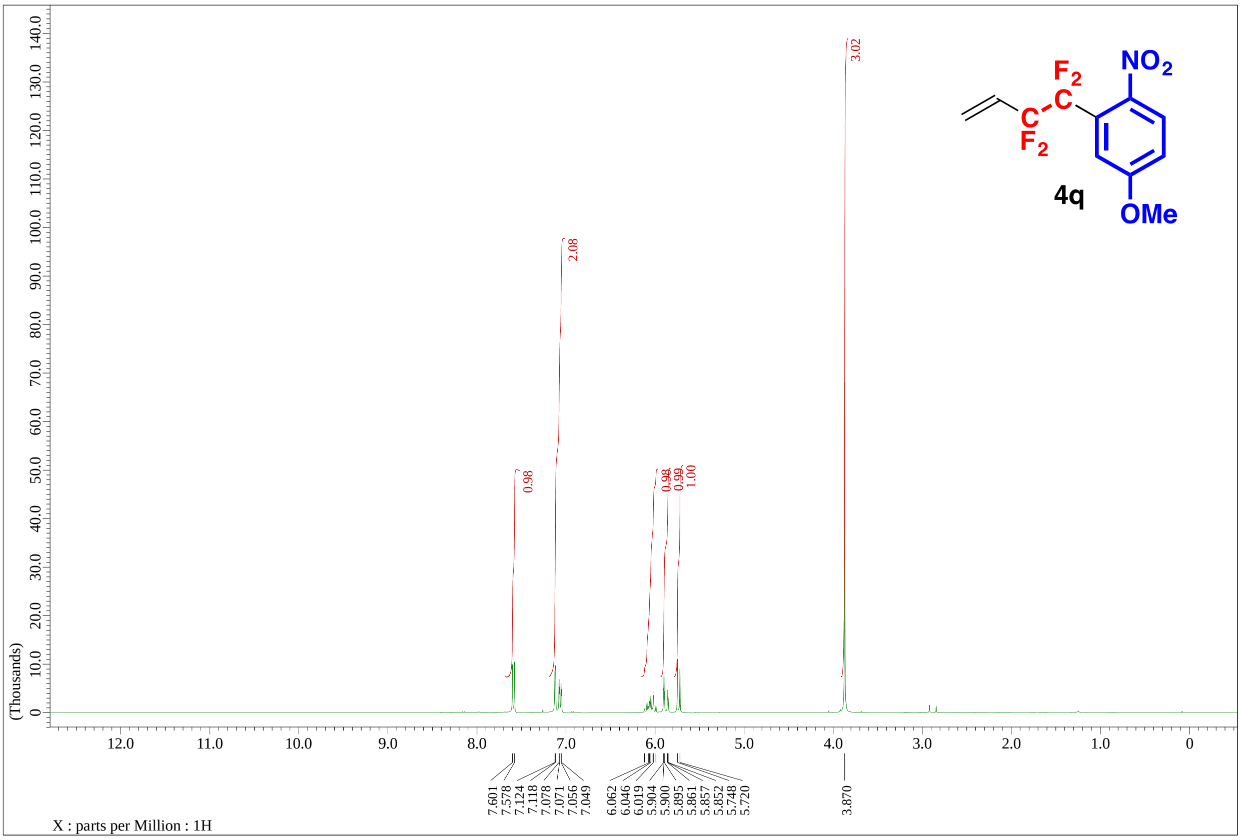

^{13}C NMR Spectrum of 3,3,4,4-tetrafluoro-4-(5-methoxy-2-nitrophenyl)but-1-ene (**4q**)

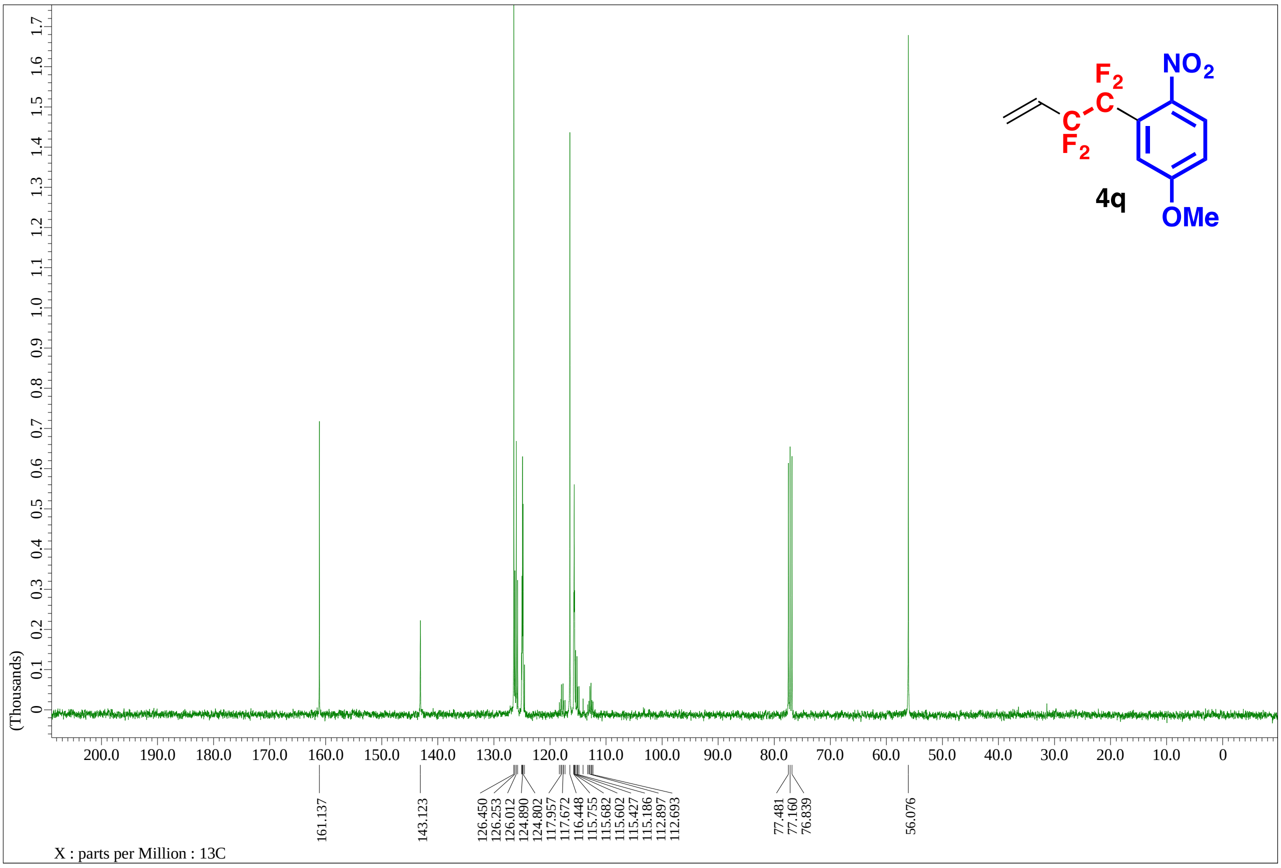

^{19}F NMR Spectrum of 3,3,4,4-tetrafluoro-4-(5-methoxy-2-nitrophenyl)but-1-ene (**4q**)

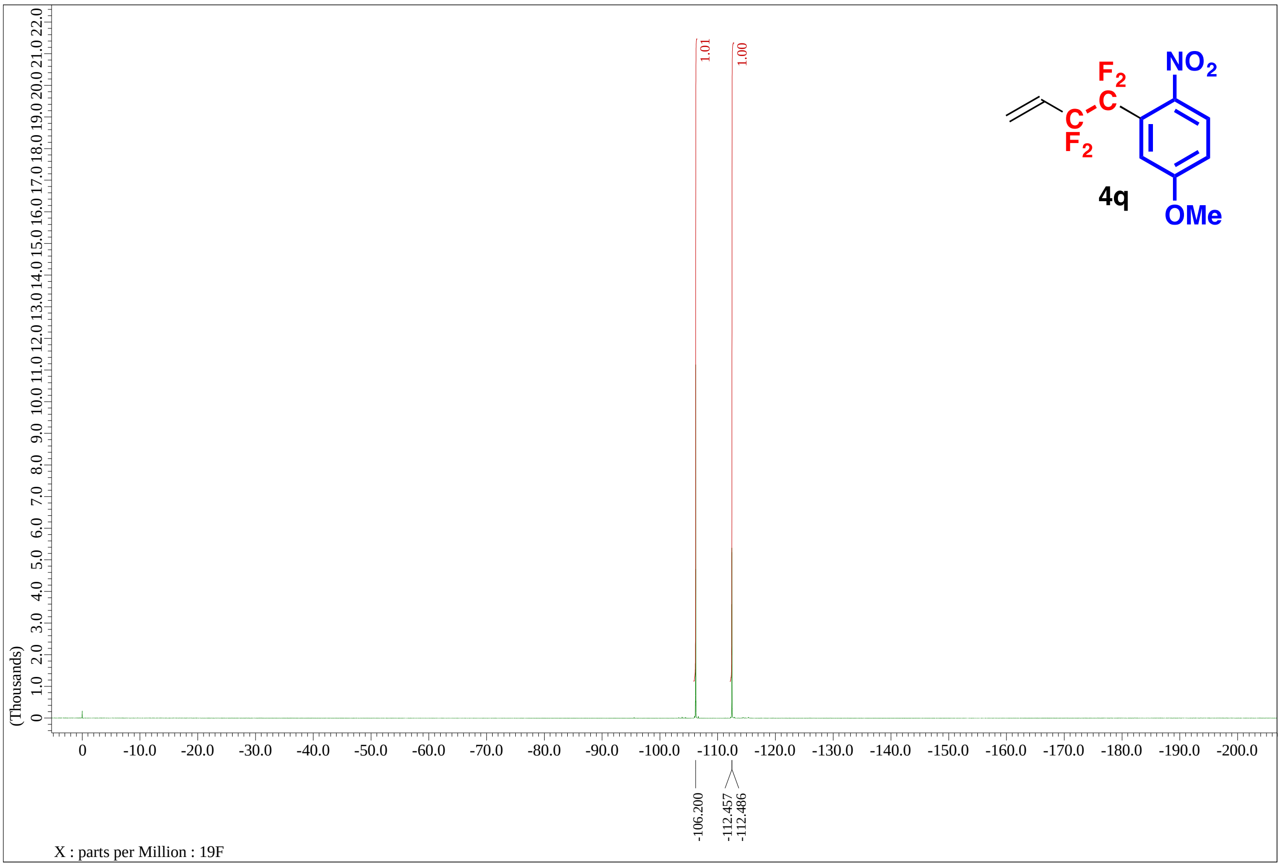

^{1}H NMR Spectrum of 2-(1,1,2,2-tetafluorobut-3-en-1-yl)aniline (**4s**)

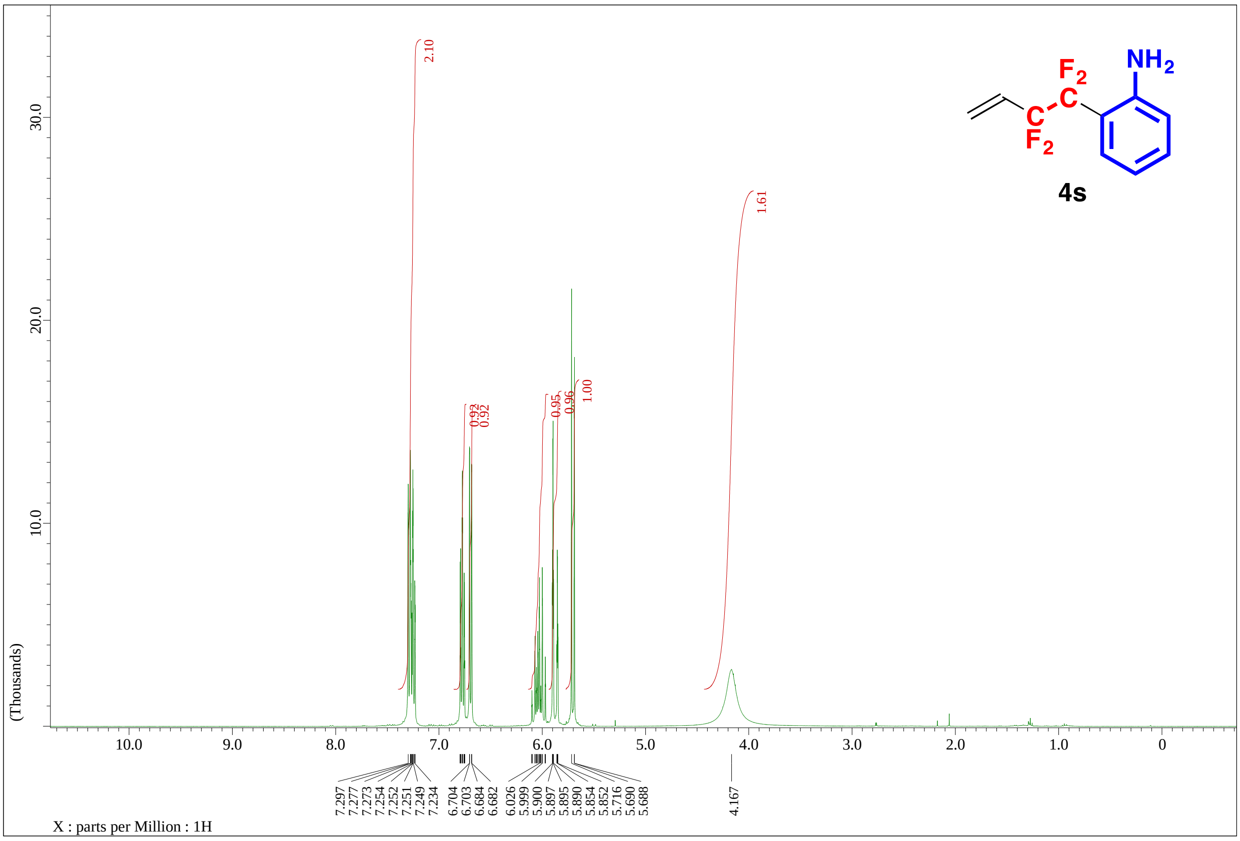

^{13}C NMR Spectrum of 2-(1,1,2,2-tetafluorobut-3-en-1-yl)aniline (**4s**)

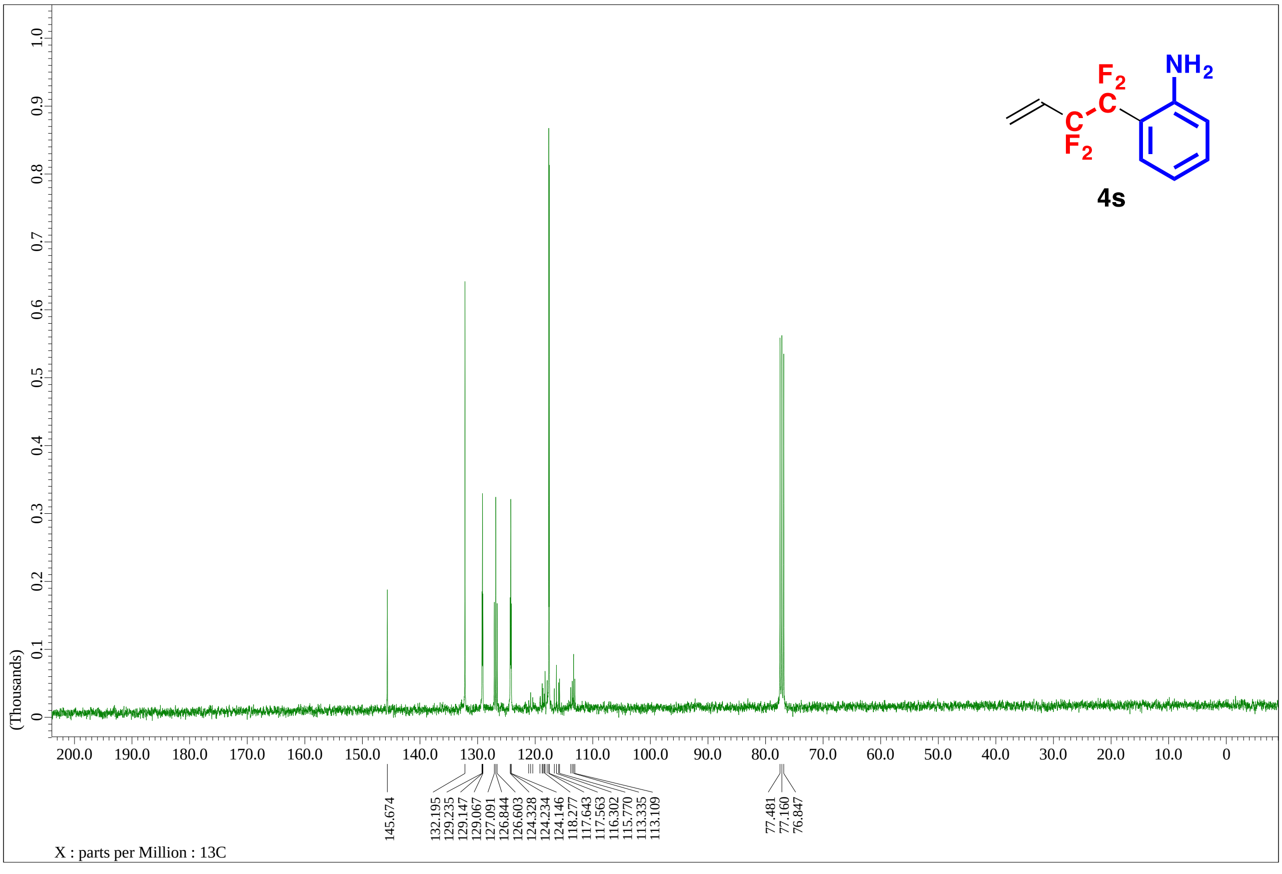

^{19}F NMR Spectrum of 2-(1,1,2,2-tetafluorobut-3-en-1-yl)aniline (**4s**)

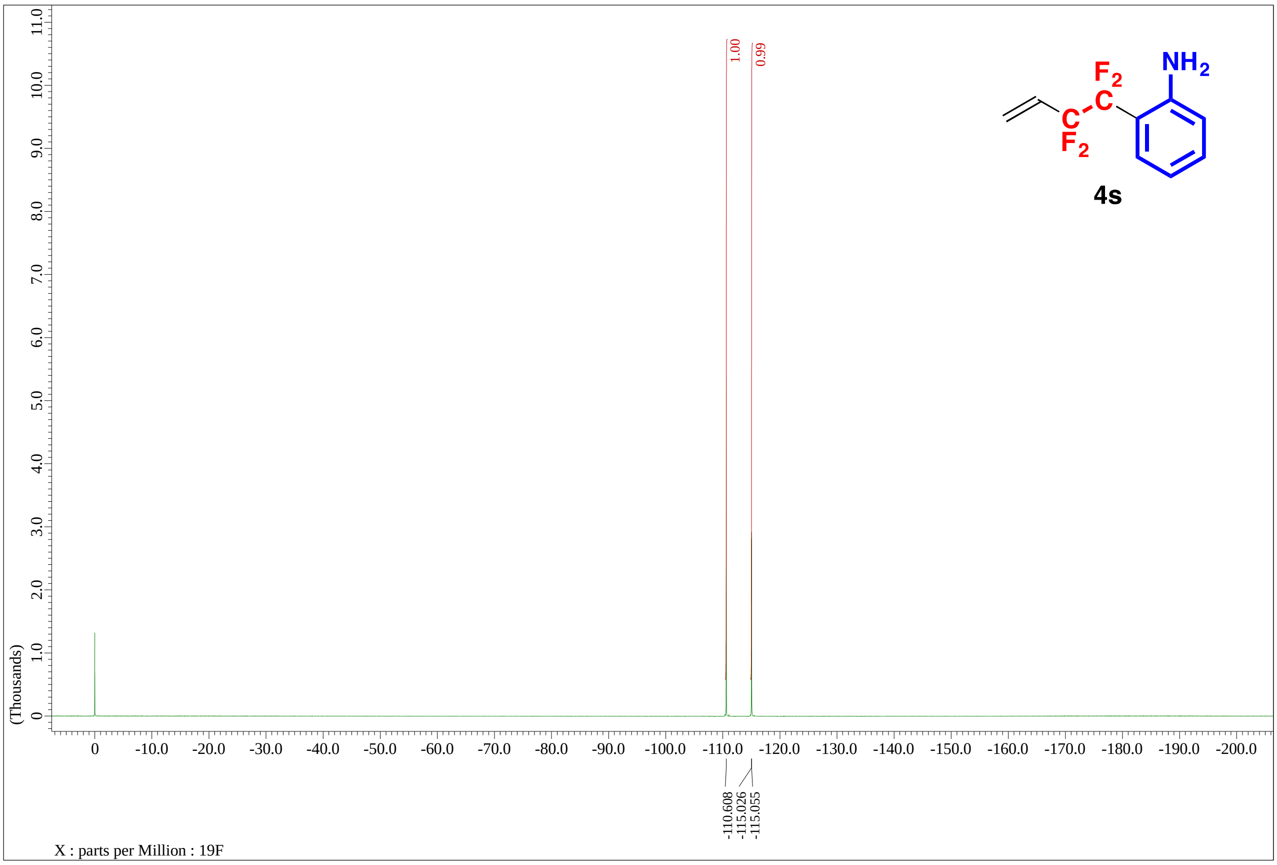

^{1}H NMR Spectrum of 3,3,4,4-tetrafluoro-4-(2-iodophenyl)but-1-ene (**4t**)

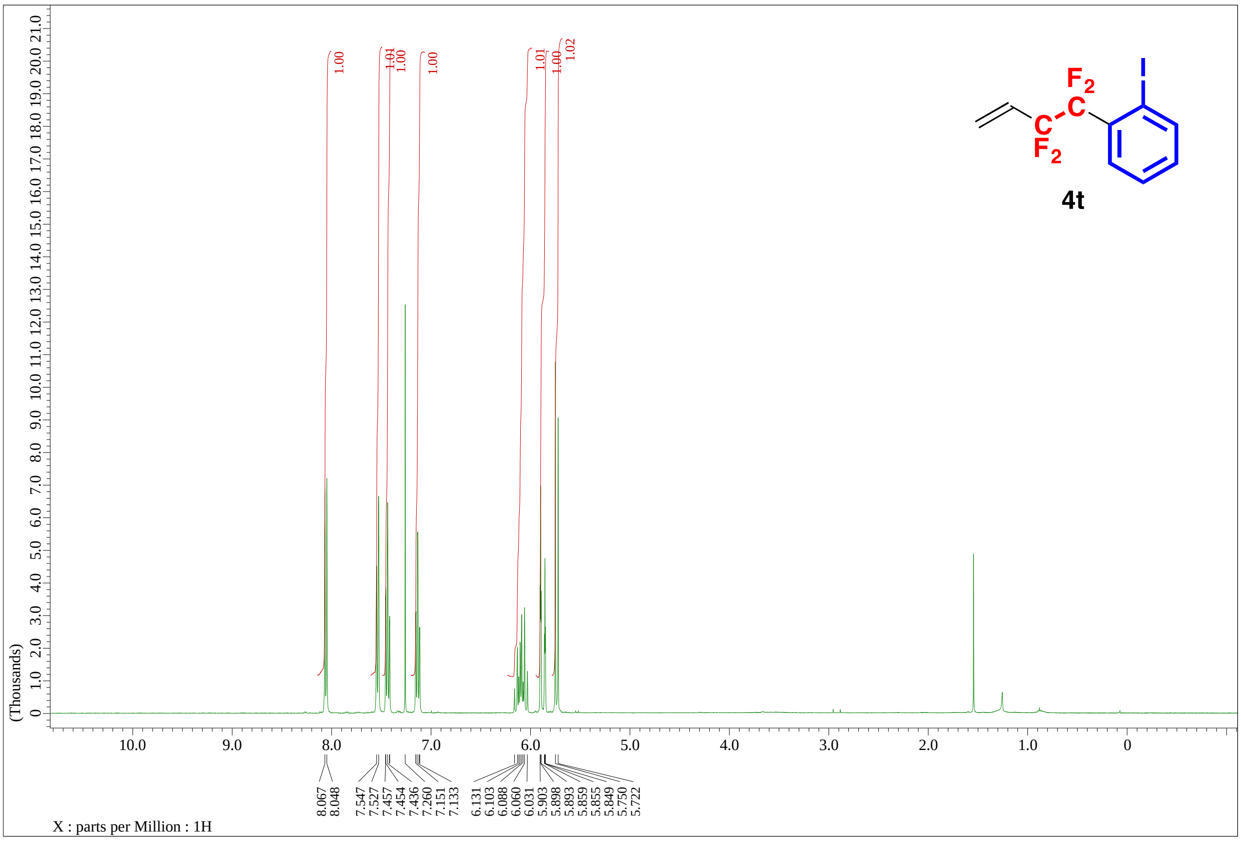

^{13}C NMR Spectrum of 3,3,4,4-tetrafluoro-4-(2-iodophenyl)but-1-ene (**4t**)

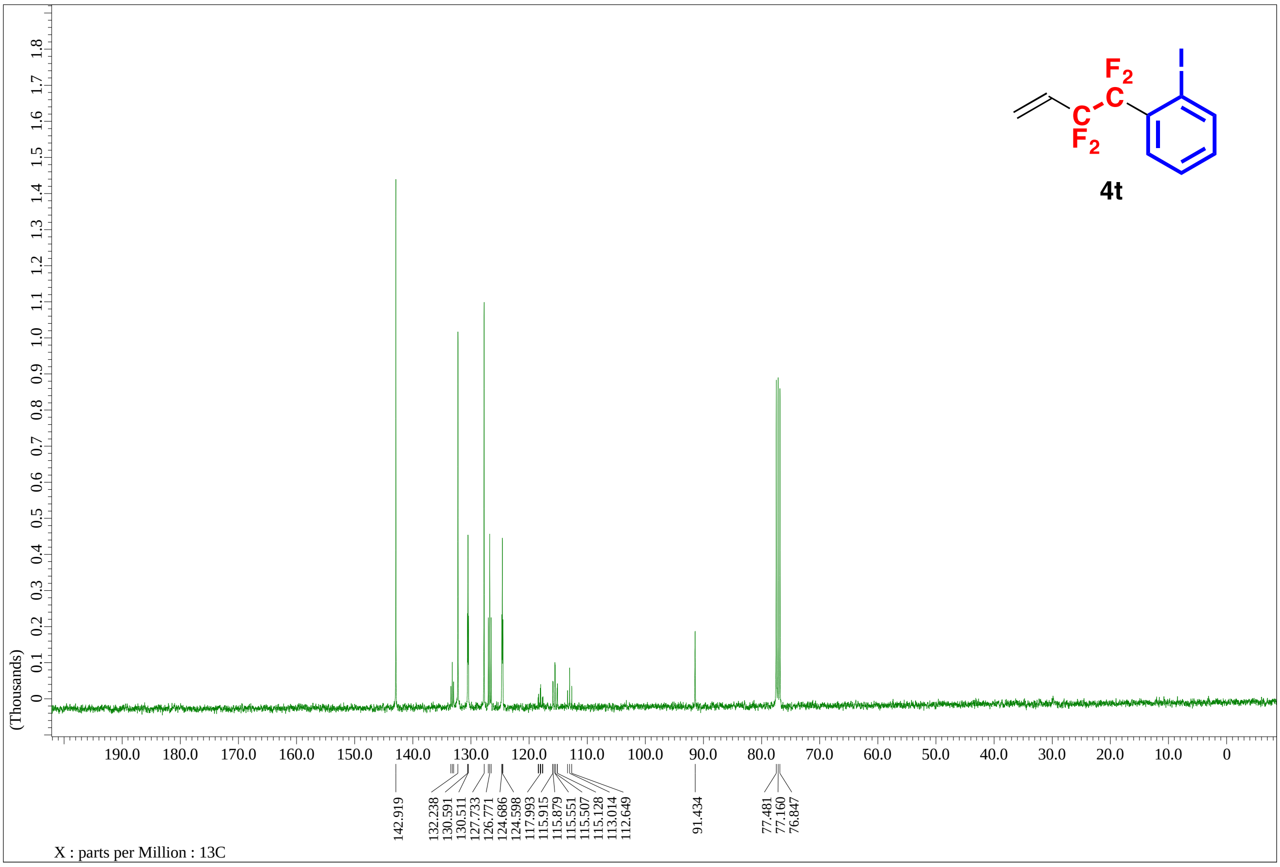

^{13}C NMR Spectrum of 3,3,4,4-tetrafluoro-4-(2-iodophenyl)but-1-ene (**4t**)

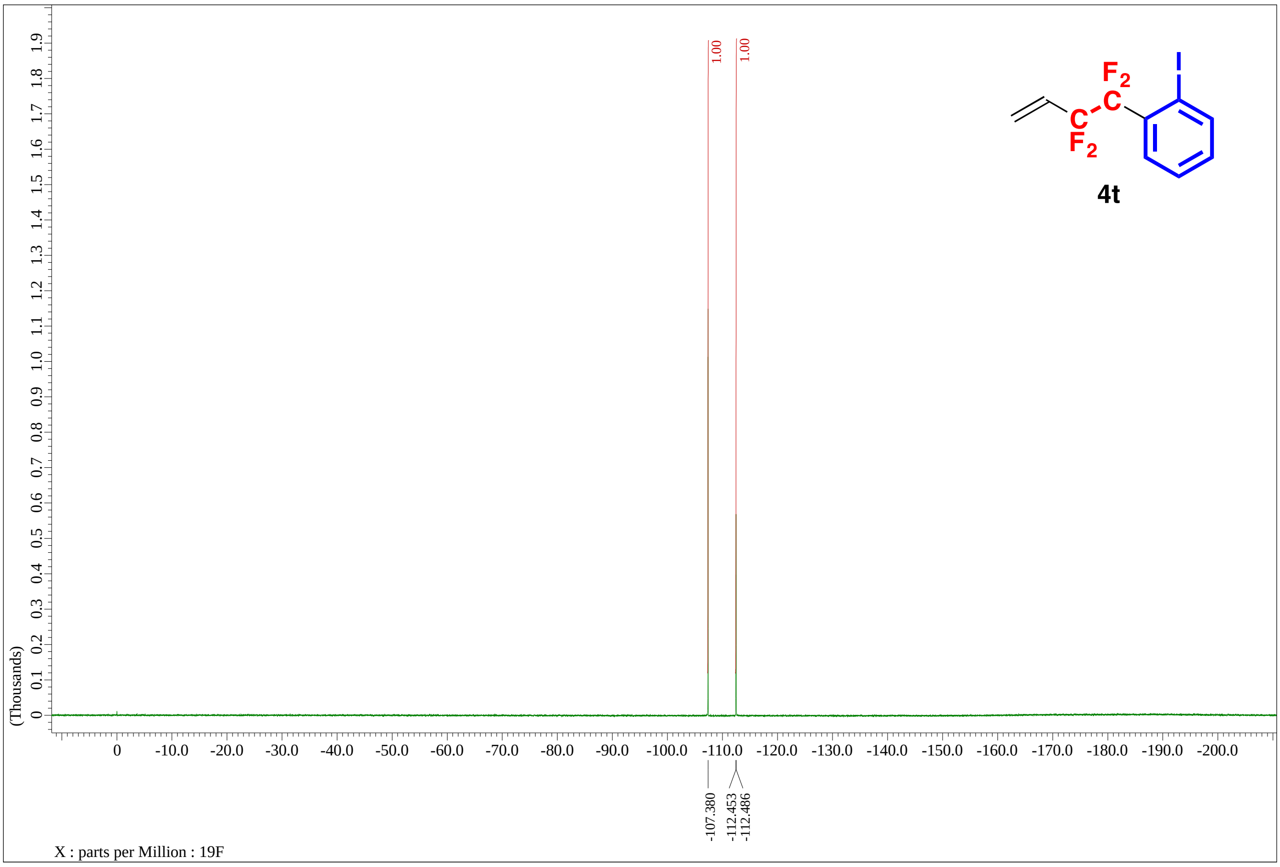

^{1}H NMR Spectrum of 1-(2-phenylethynyl)-2-(2,2,3,3-tetrafluorobut-3-en-1-yl)benzene (**4u**)

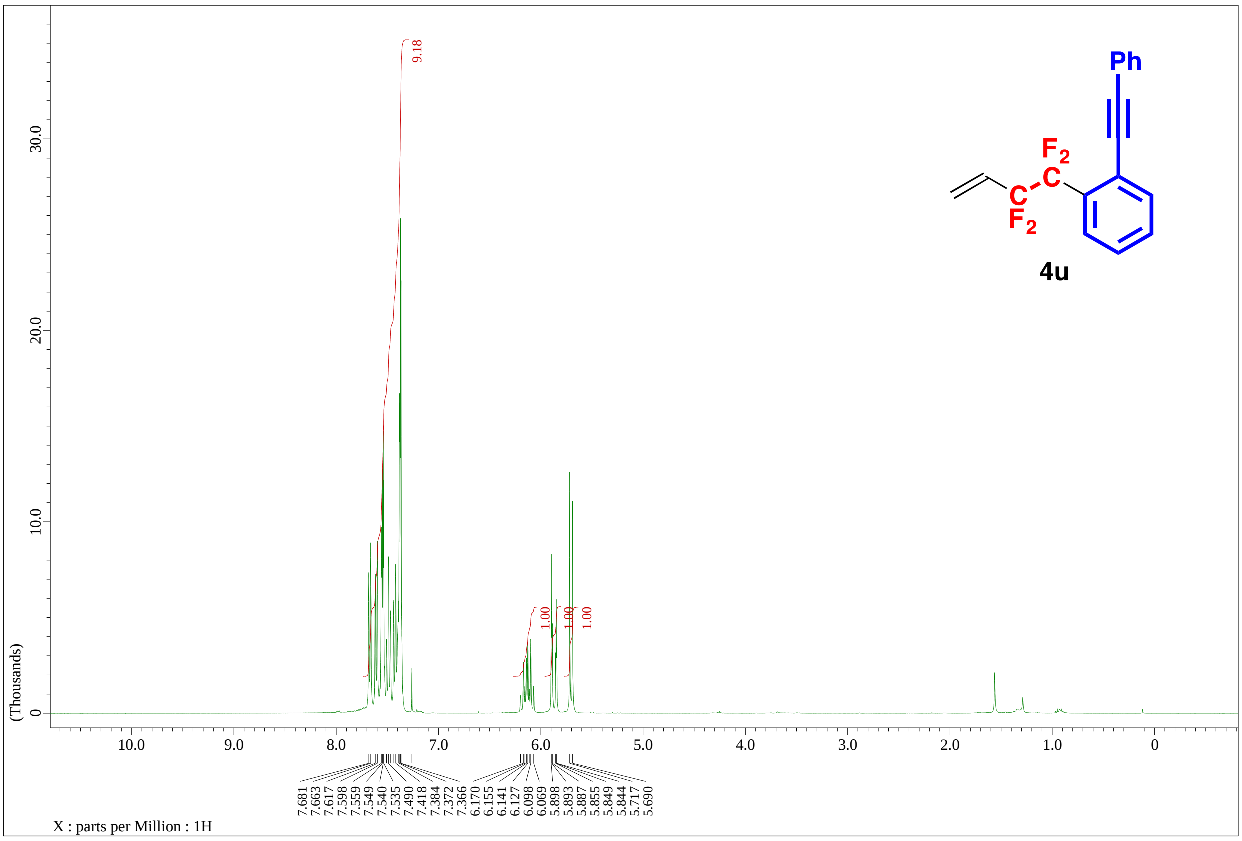

^{13}C NMR Spectrum of 1-(2-phenylethynyl)-2-(2,2,3,3-tetrafluorobut-3-en-1-yl)benzene (**4u**)

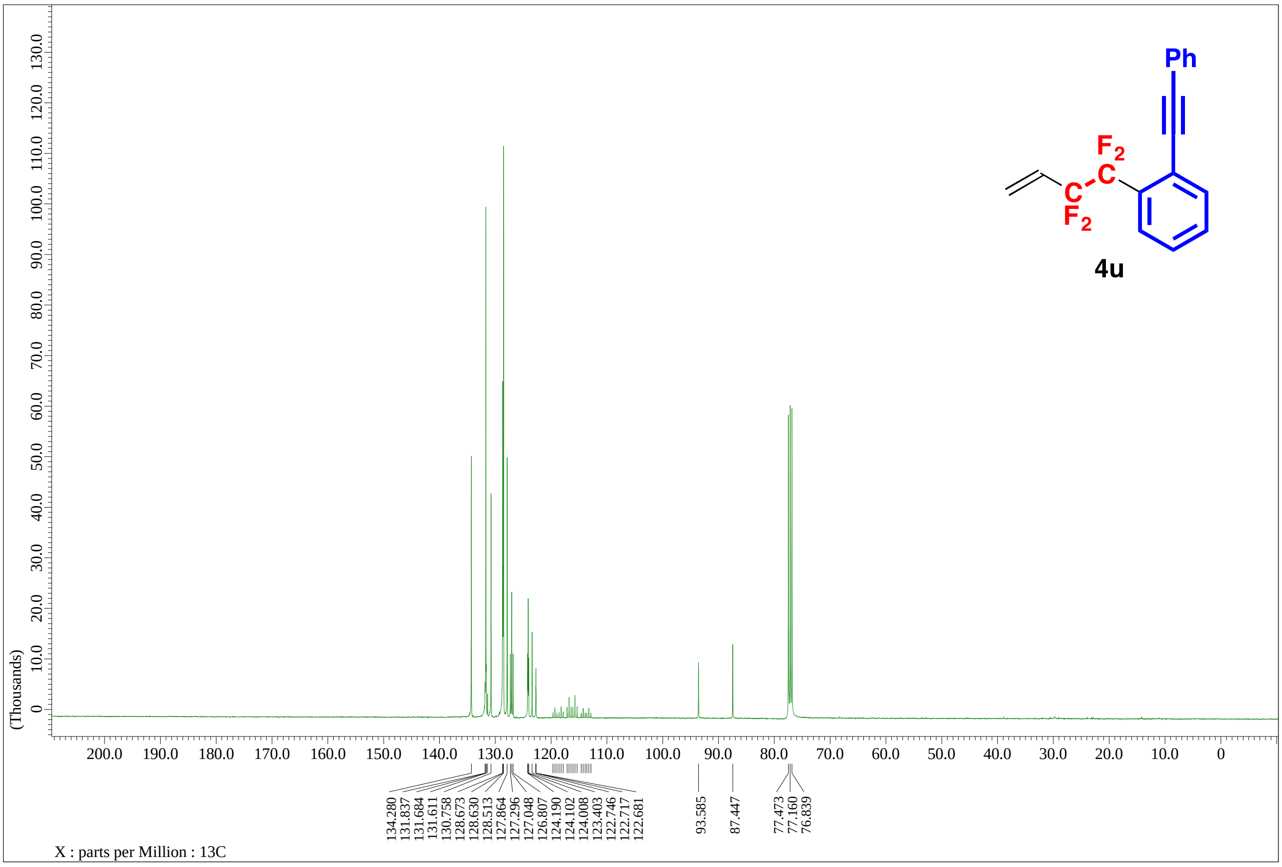

^{19}F NMR Spectrum of 1-(2-phenylethynyl)-2-(2,2,3,3-tetrafluorobut-3-en-1-yl)benzene (**4u**)

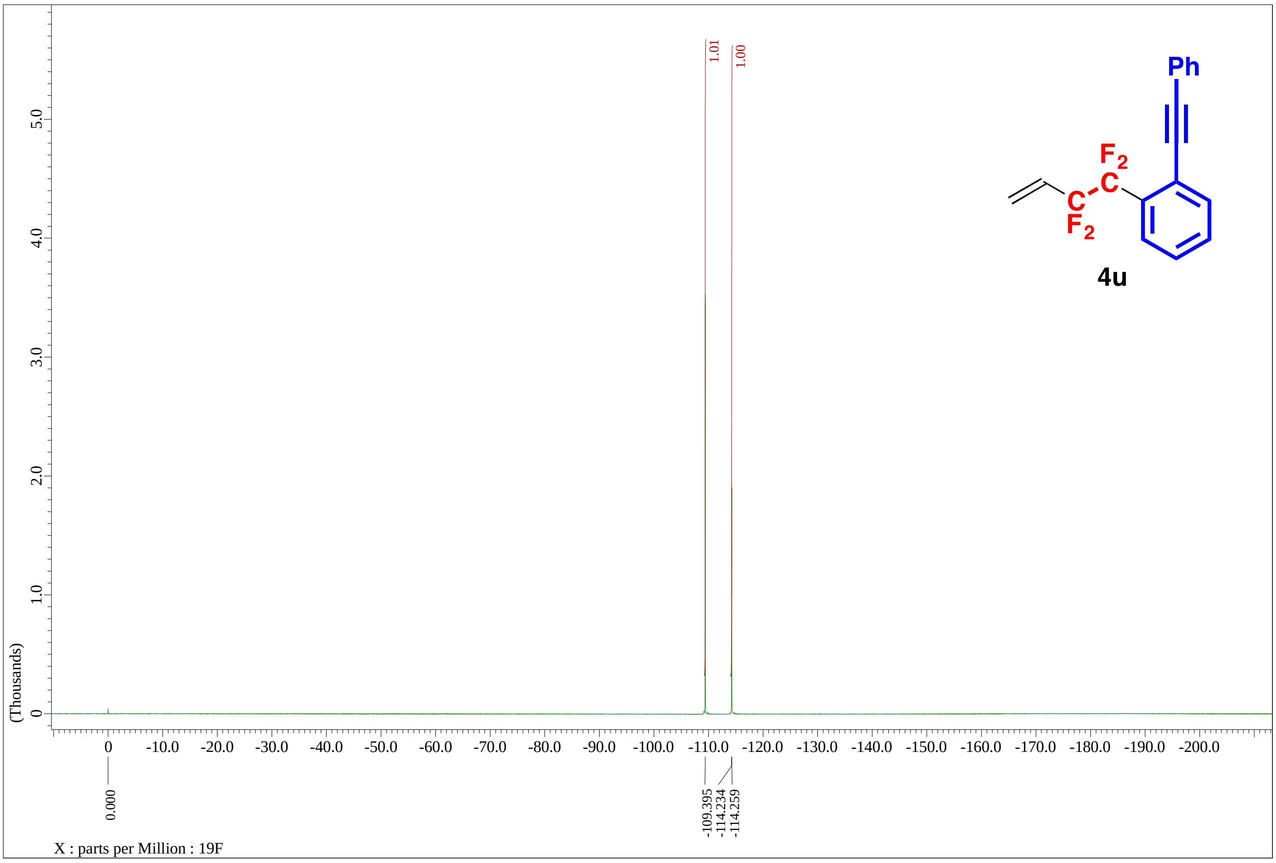

^{1}H NMR Spectrum of 2,2,3,3-tetrafluoro-1-phenylpent-4-en-1-one (**6a**)

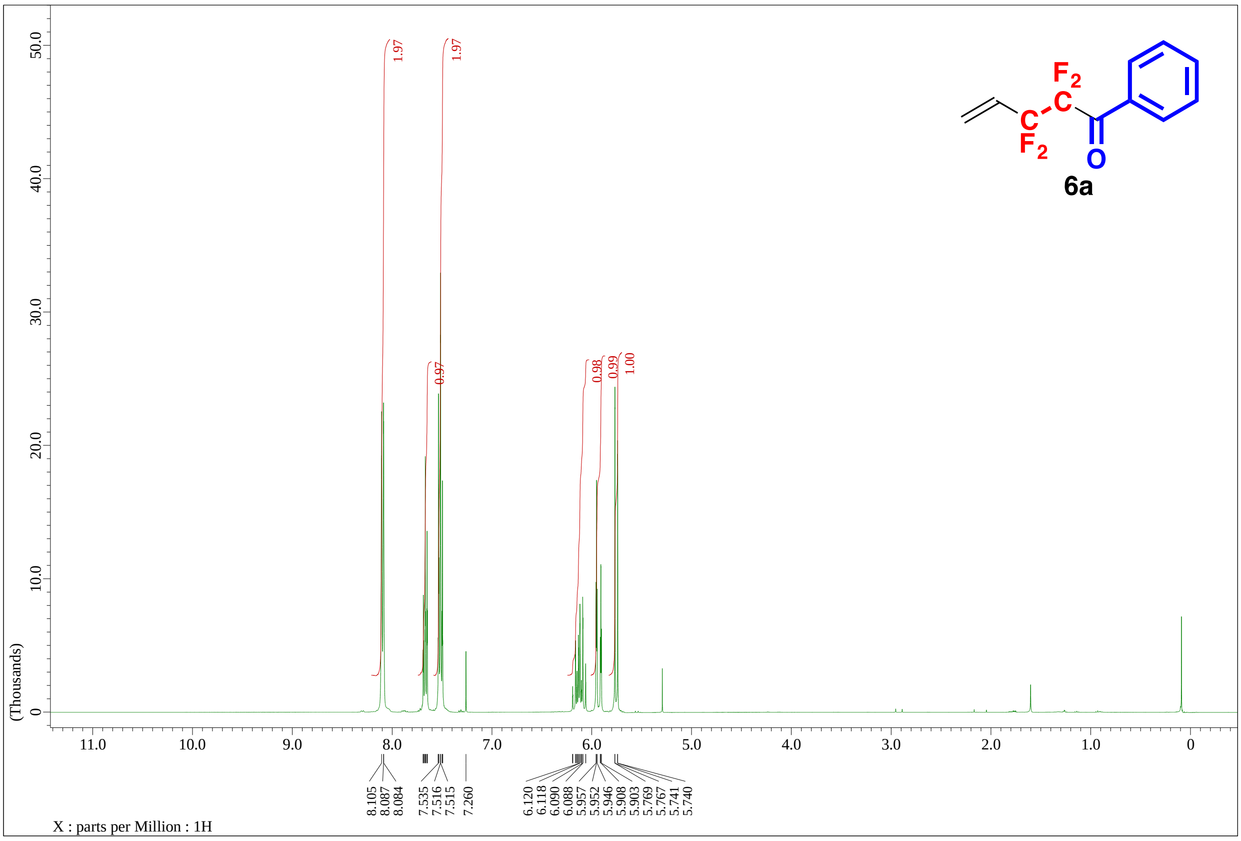

^{13}C NMR Spectrum of 2,2,3,3-tetrafluoro-1-phenylpent-4-en-1-one (**6a**)

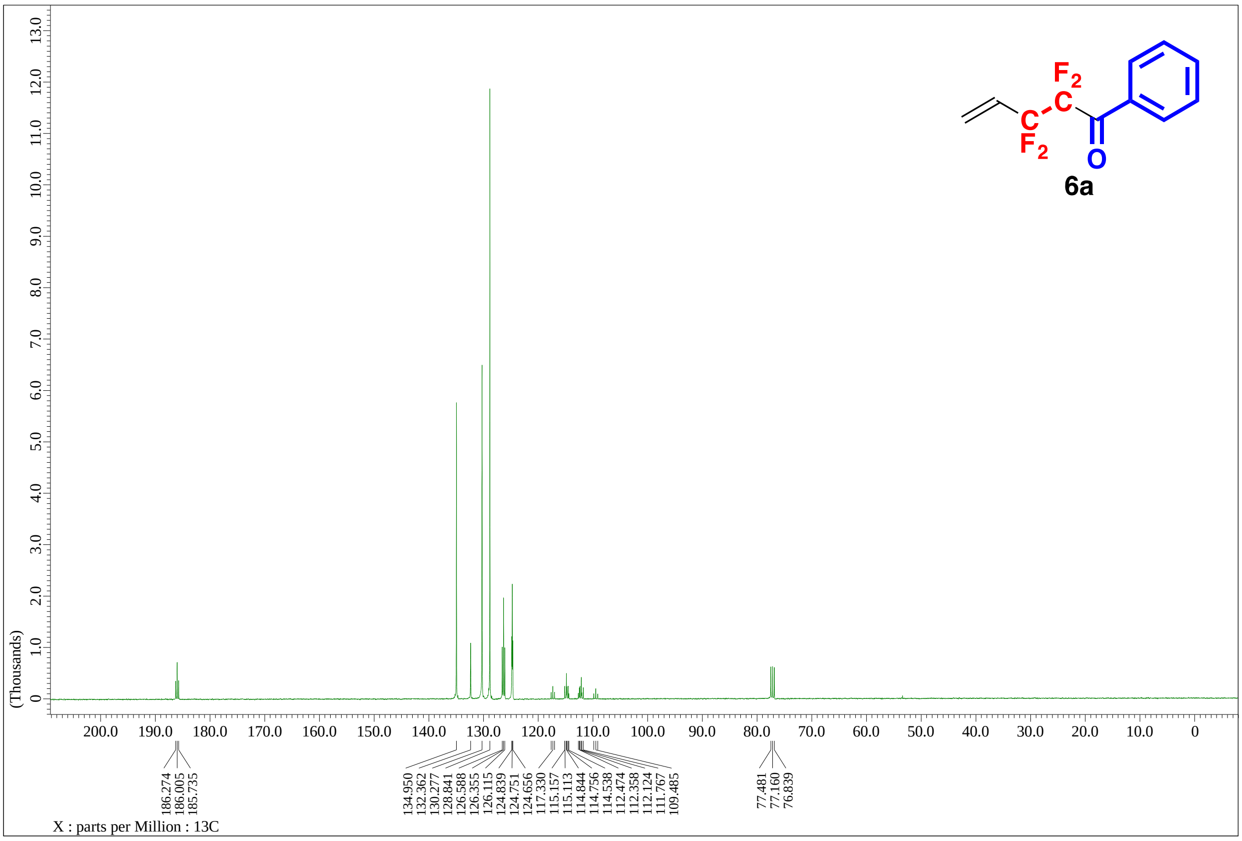

^{19}F NMR Spectrum of 2,2,3,3-tetrafluoro-1-phenylpent-4-en-1-one (**6a**)

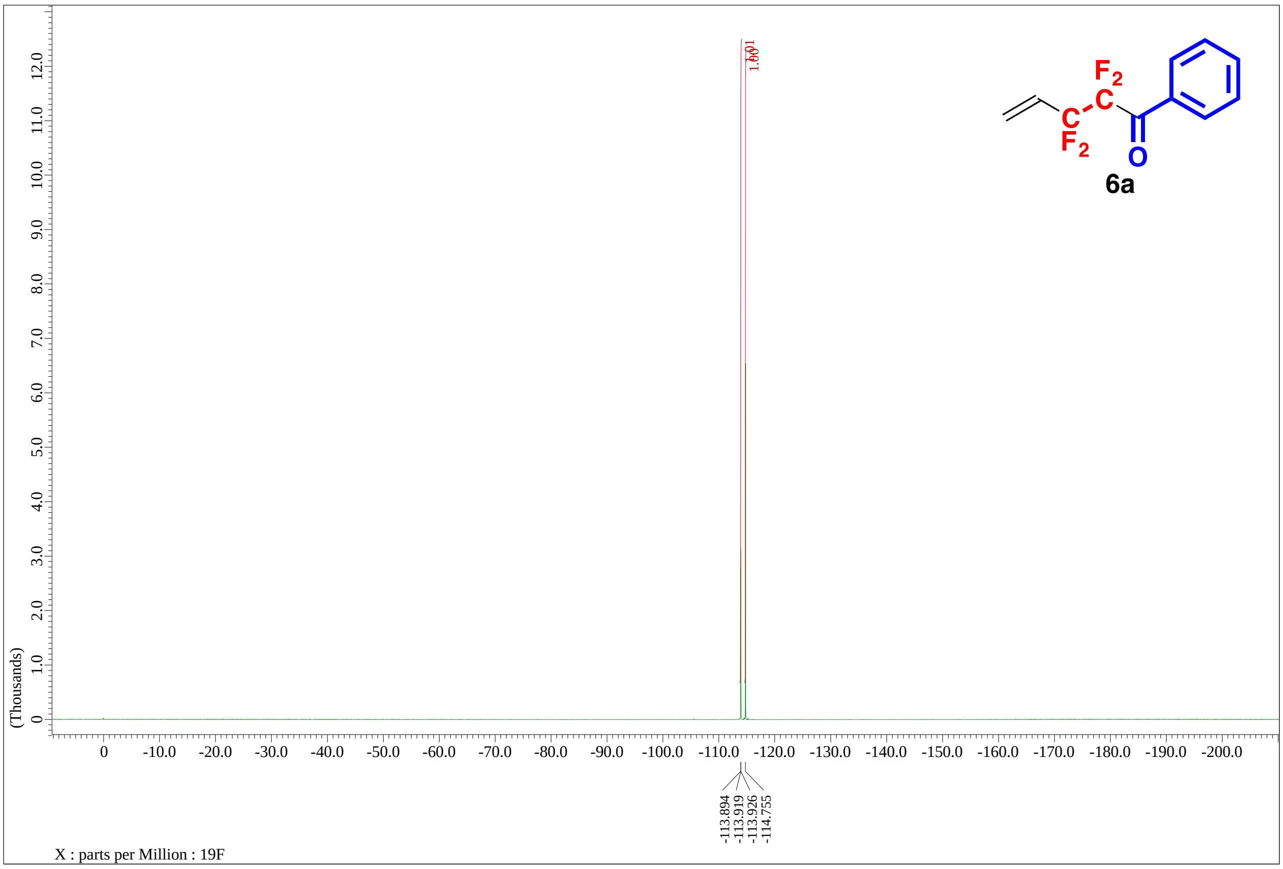

^{1}H NMR Spectrum of 2,2,3,3-tetrafluoro-1-(4-methylphenyl)pent-4-en-1-one (**6b**)

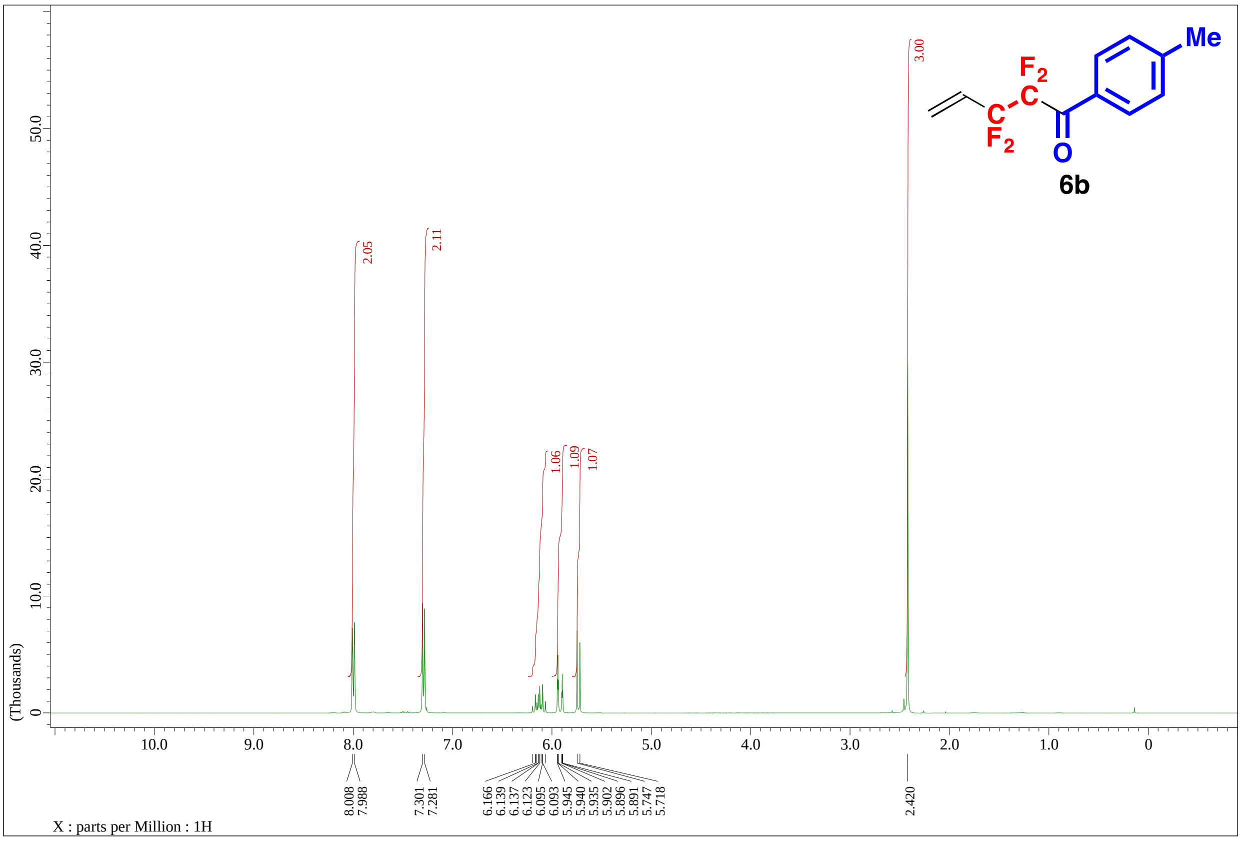

^{13}C NMR Spectrum of 2,2,3,3-tetrafluoro-1-(4-methylphenyl)pent-4-en-1-one (**6b**)

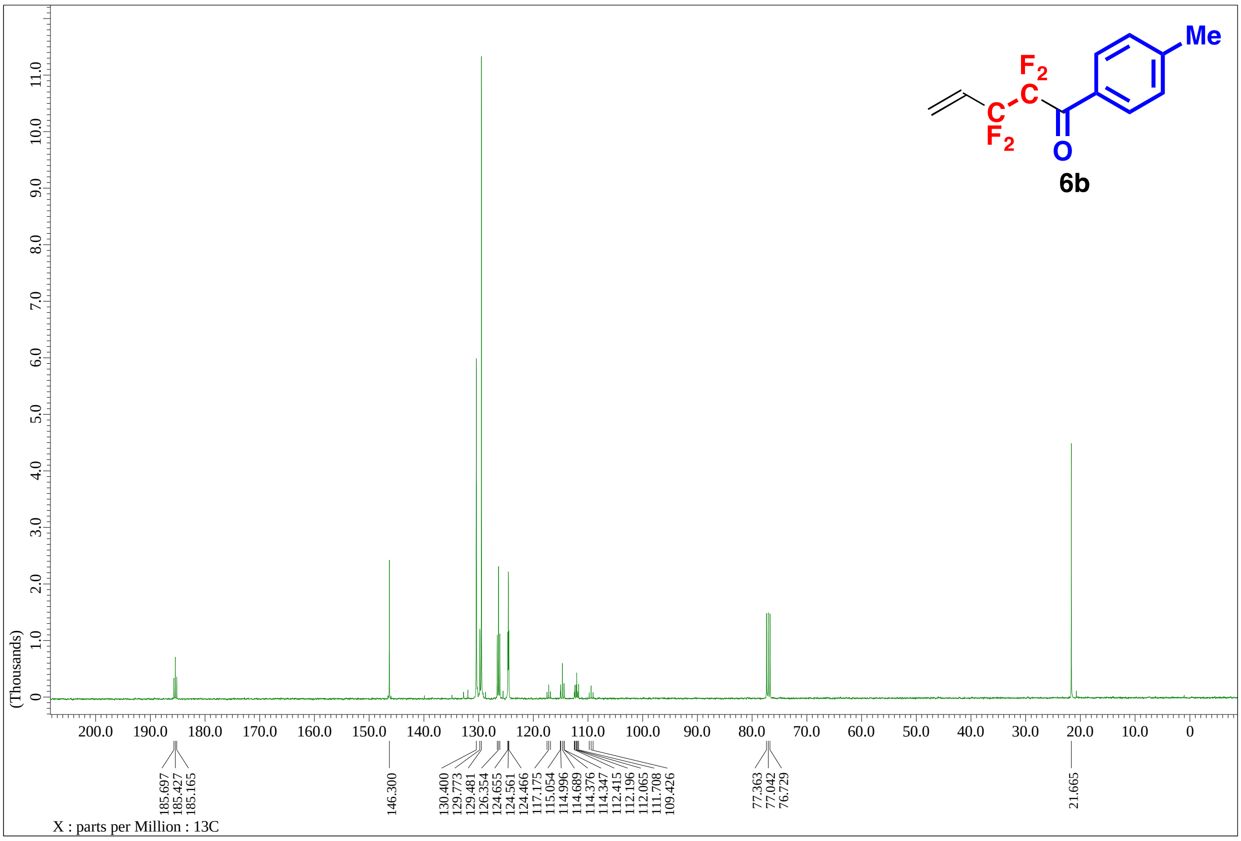

^{19}F NMR Spectrum of 2,2,3,3-tetrafluoro-1-(4-methylphenyl)pent-4-en-1-one (**6b**)

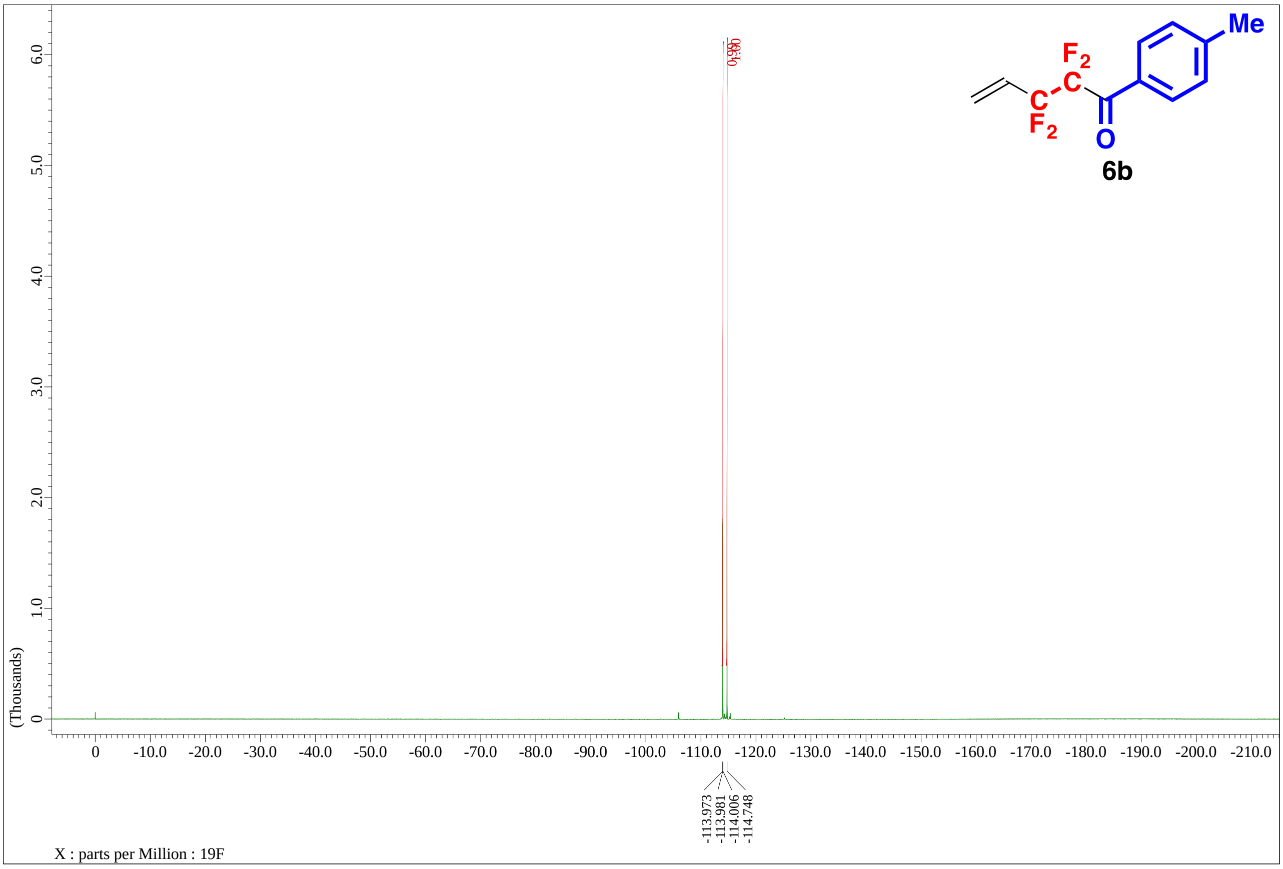

^{1}H NMR Spectrum of 2,2,3,3-tetrafluoro-1-(2-methylphenyl)pent-4-en-1-one (**6c**)

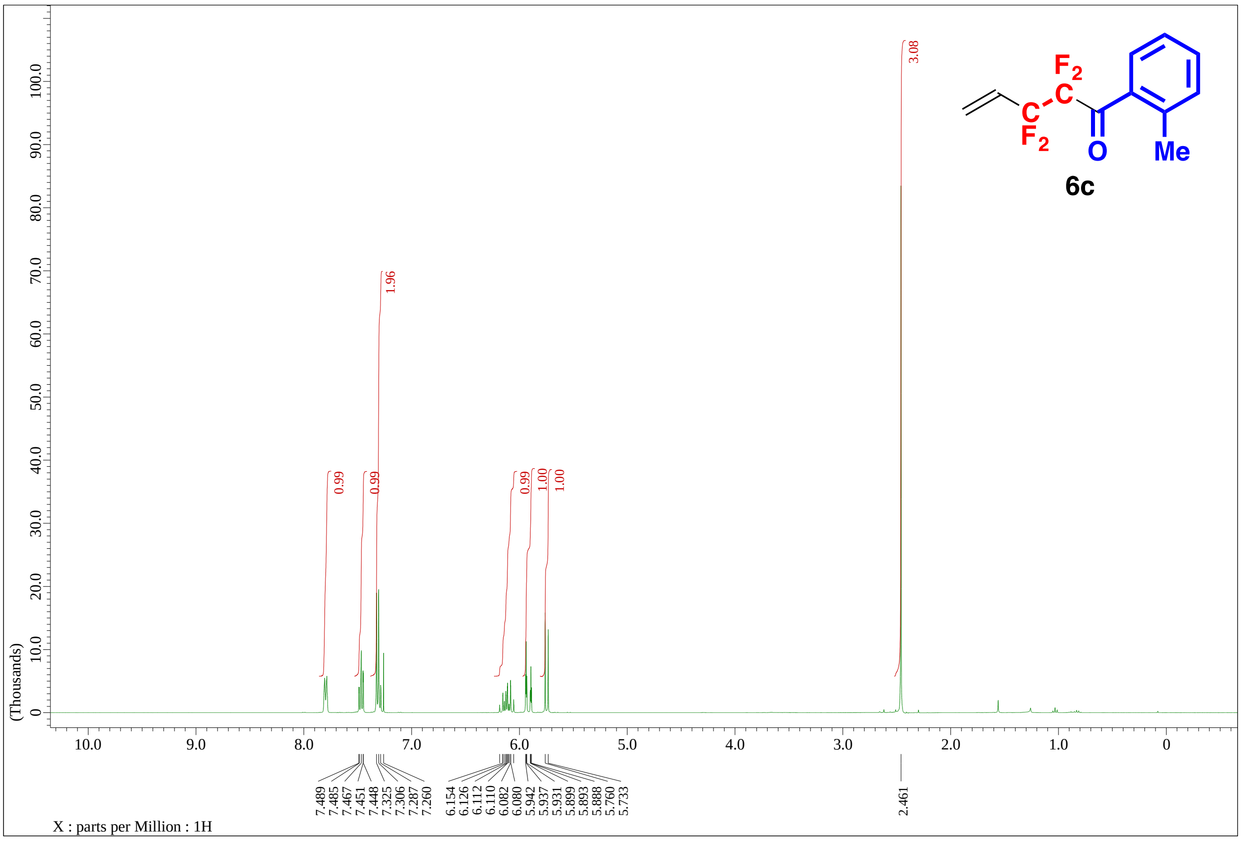

^{13}C NMR Spectrum of 2,2,3,3-tetrafluoro-1-(2-methylphenyl)pent-4-en-1-one (**6c**)

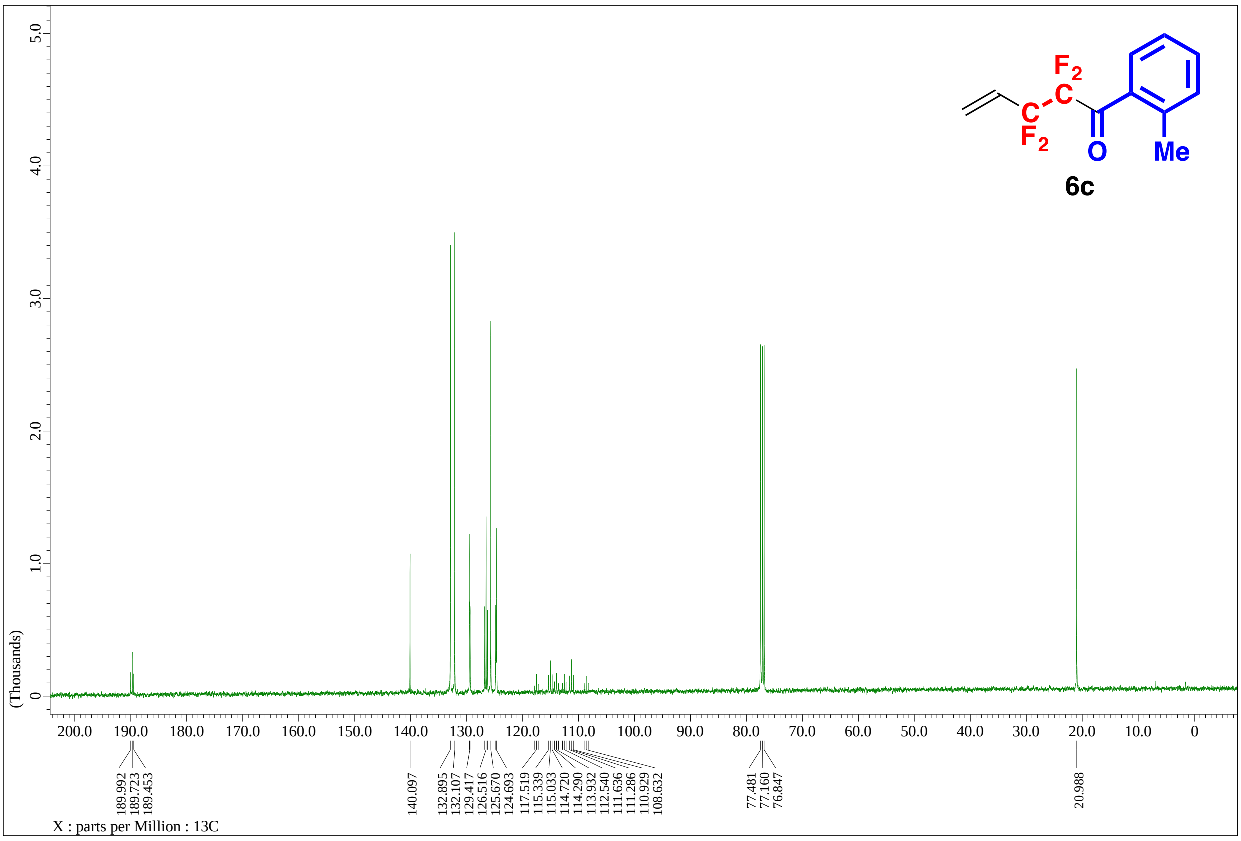

^{19}F NMR Spectrum of 2,2,3,3-tetrafluoro-1-(2-methylphenyl)pent-4-en-1-one (**6c**)

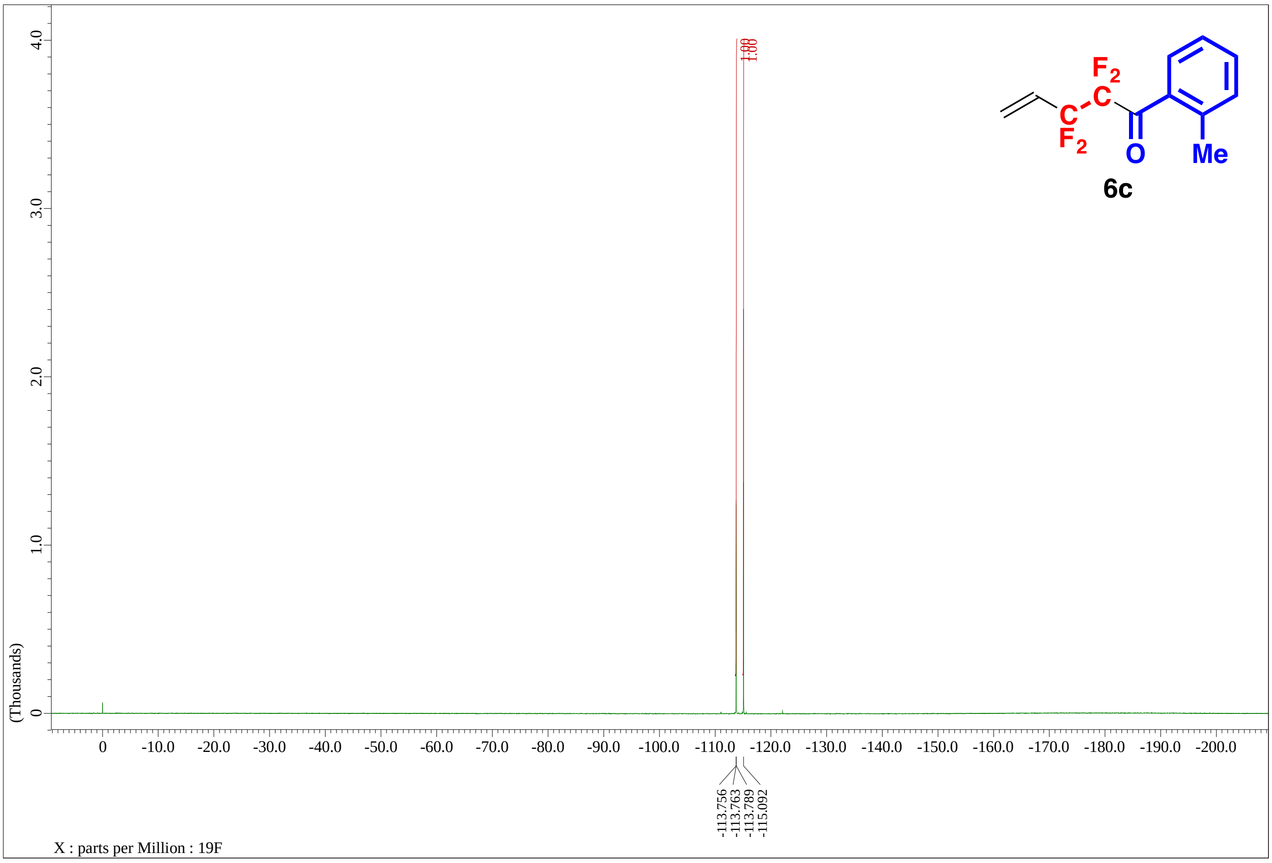

^{1}H NMR Spectrum of 1-(4-*tert*-butylphenyl)-2,2,3,3-tetrafluoropent-4-en-1-one (**6d**)

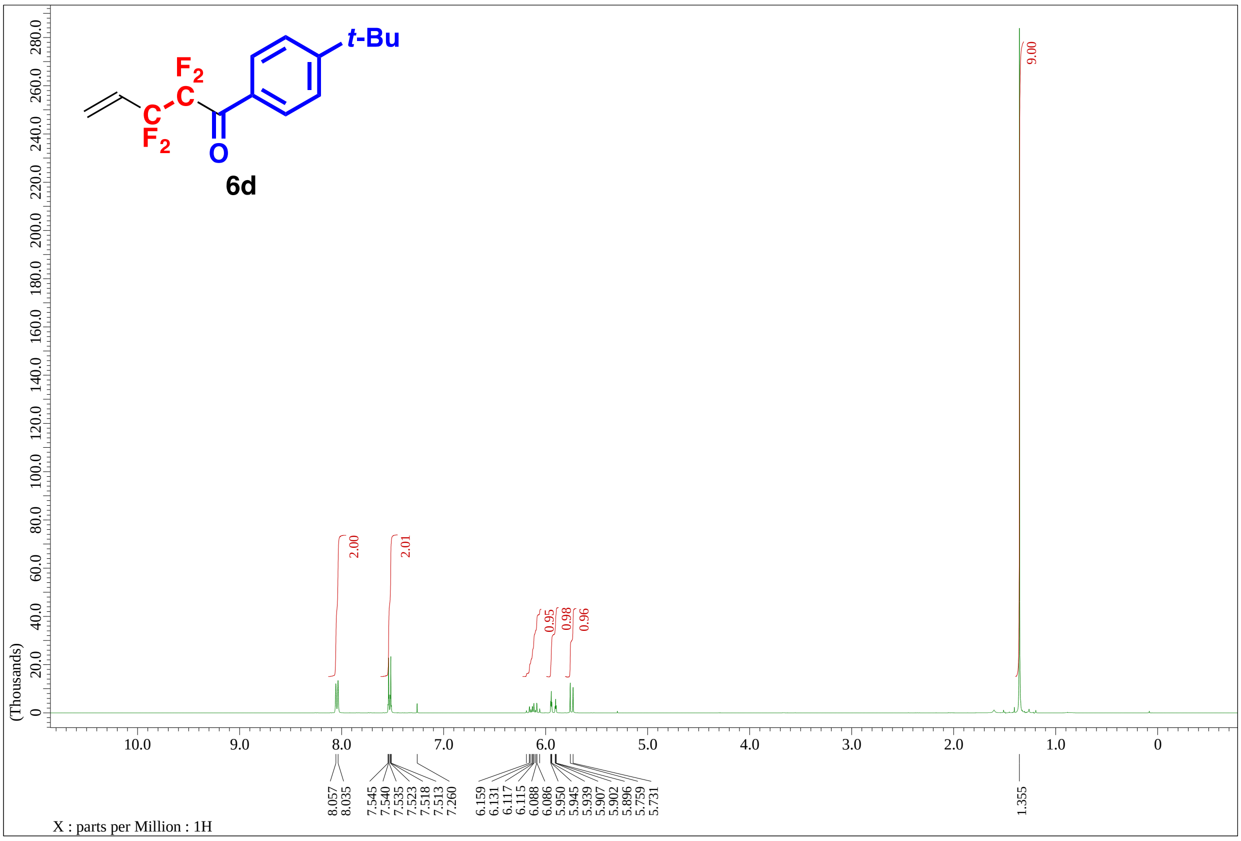

^{13}C NMR Spectrum of 1-(4-*tert*-butylphenyl)-2,2,3,3-tetrafluoropent-4-en-1-one (**6d**)

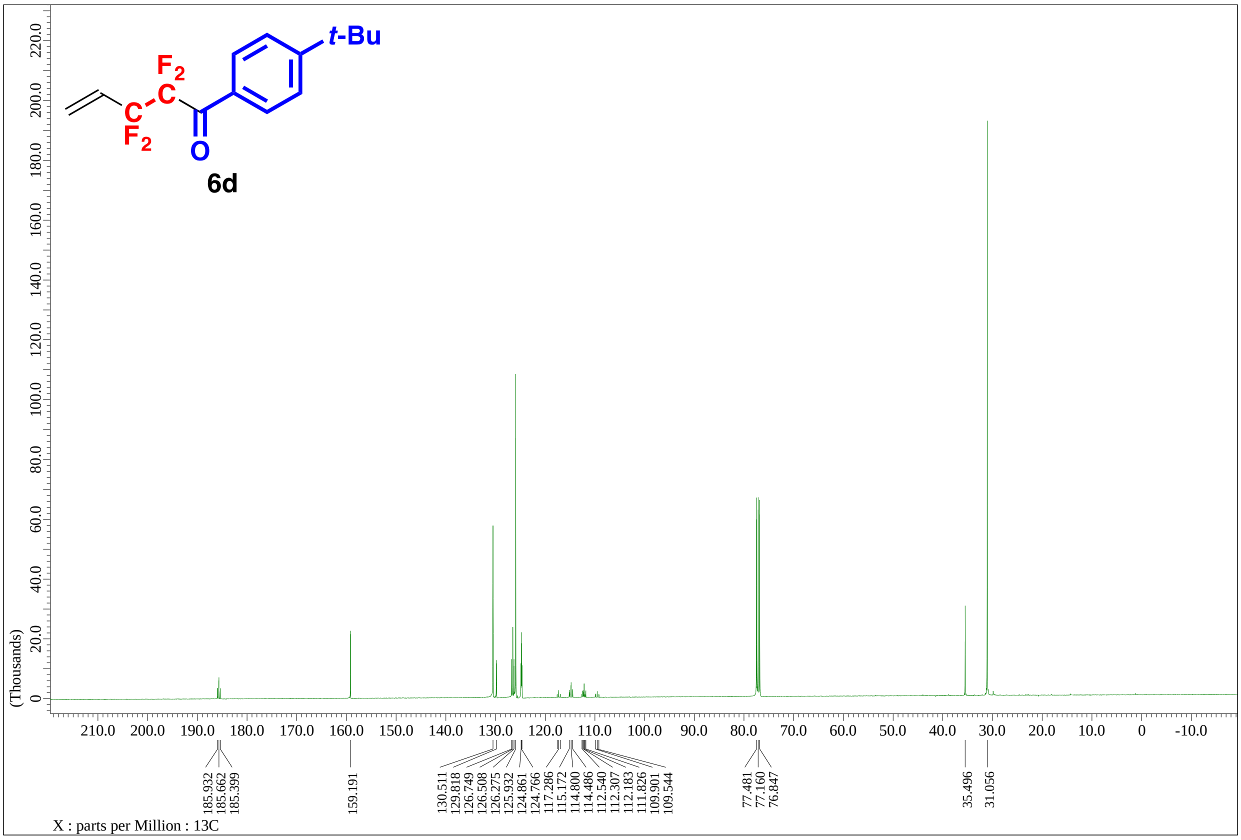

^{19}F NMR Spectrum of 1-(4-*tert*-butylphenyl)-2,2,3,3-tetrafluoropent-4-en-1-one (**6d**)

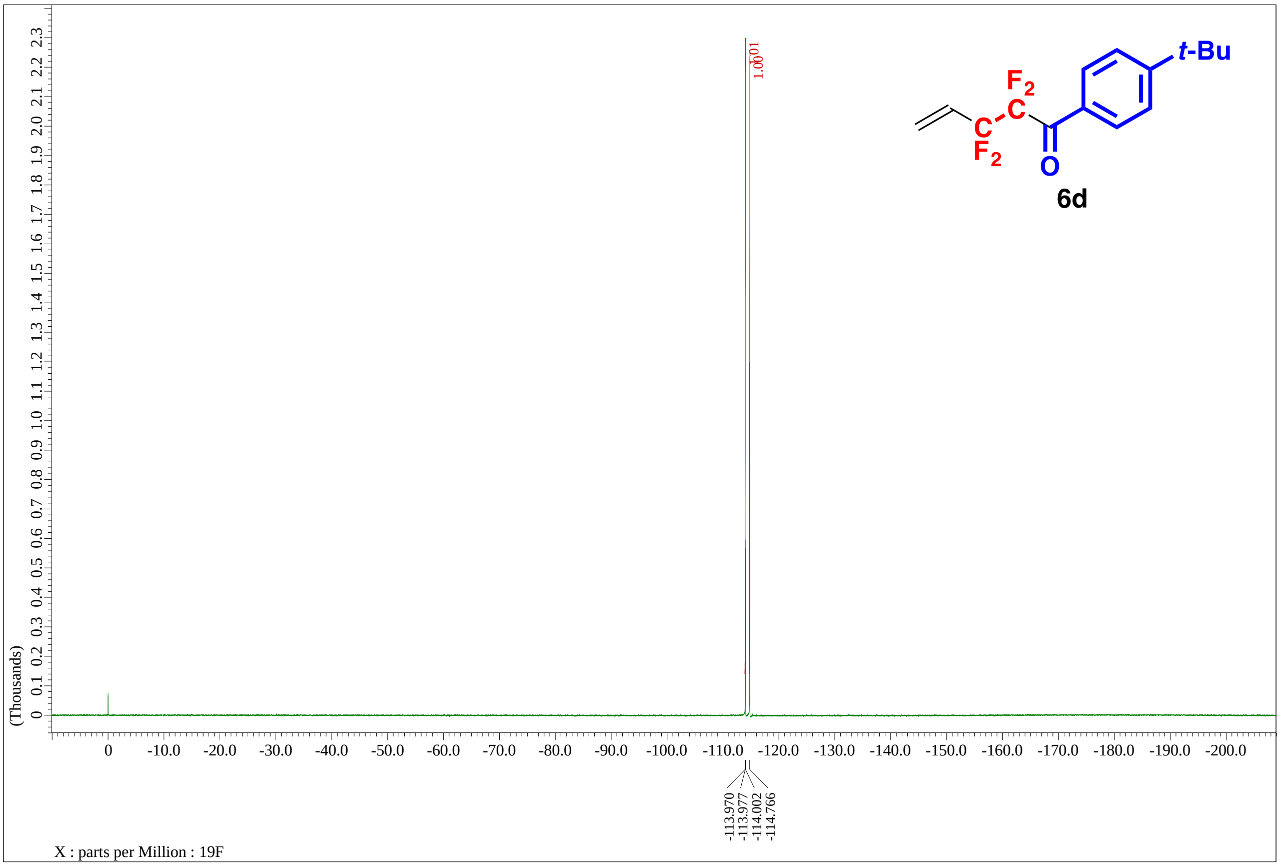

^{1}H NMR Spectrum of 1-(4-bromophenyl)-2,2,3,3-tetrafluoropent-4-en-1-one (**6e**)

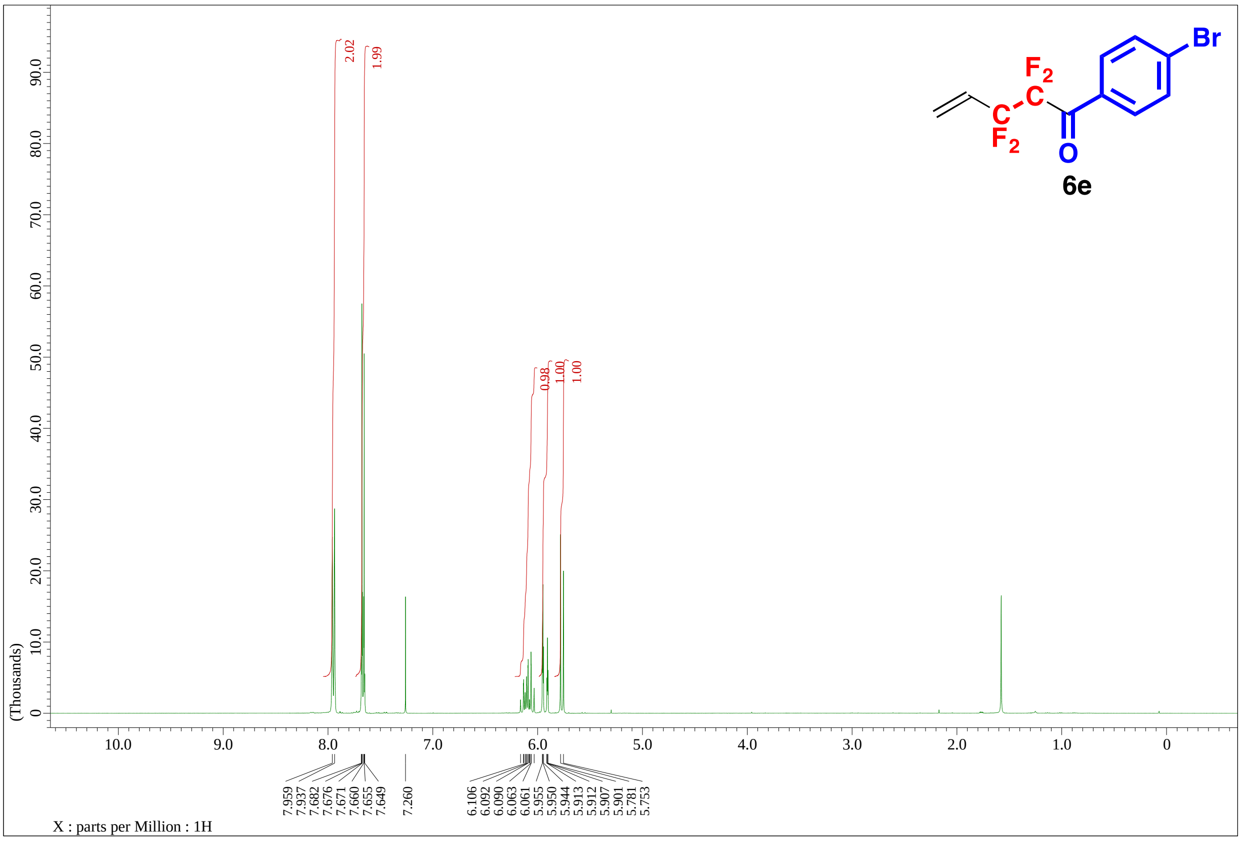

^{13}C NMR Spectrum of 1-(4-bromophenyl)-2,2,3,3-tetrafluoropent-4-en-1-one (**6e**)

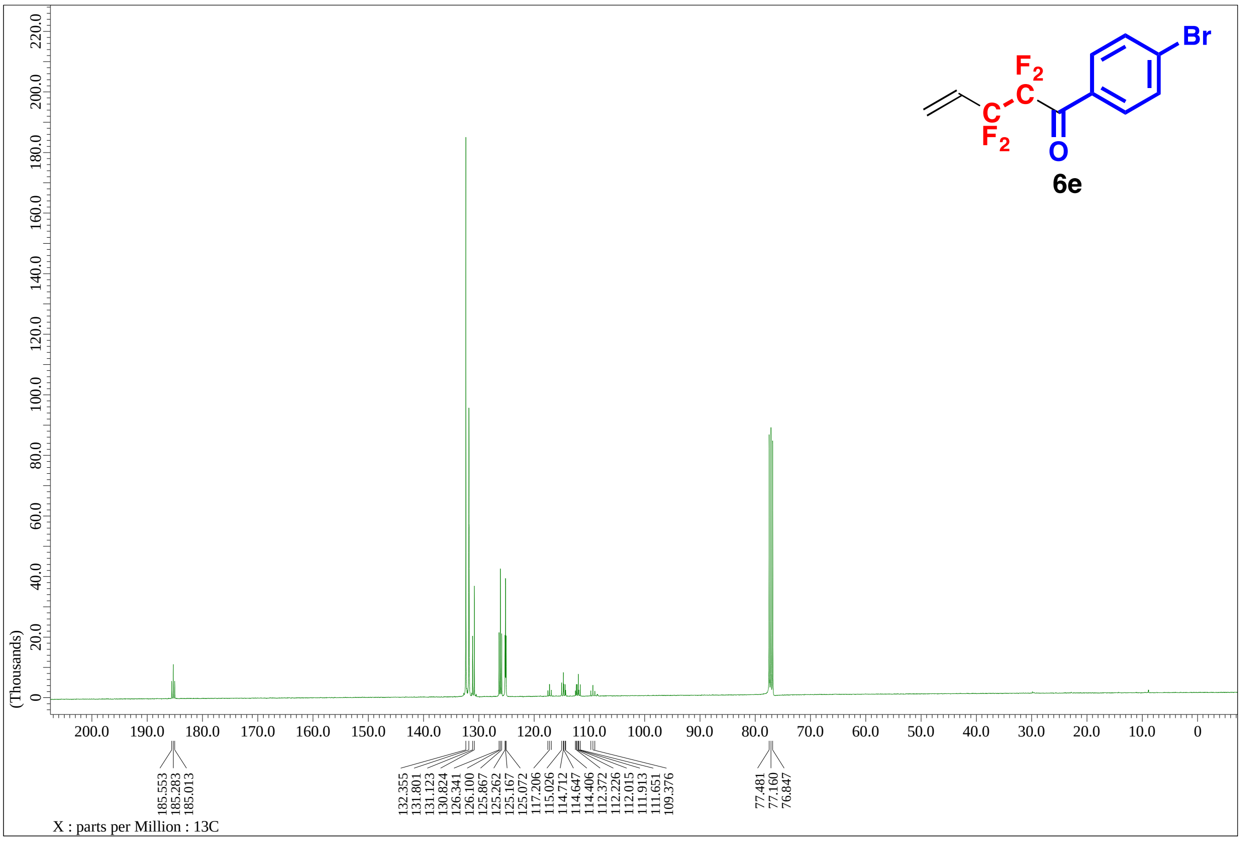

^{19}F NMR Spectrum of 1-(4-bromophenyl)-2,2,3,3-tetrafluoropent-4-en-1-one (**6e**)

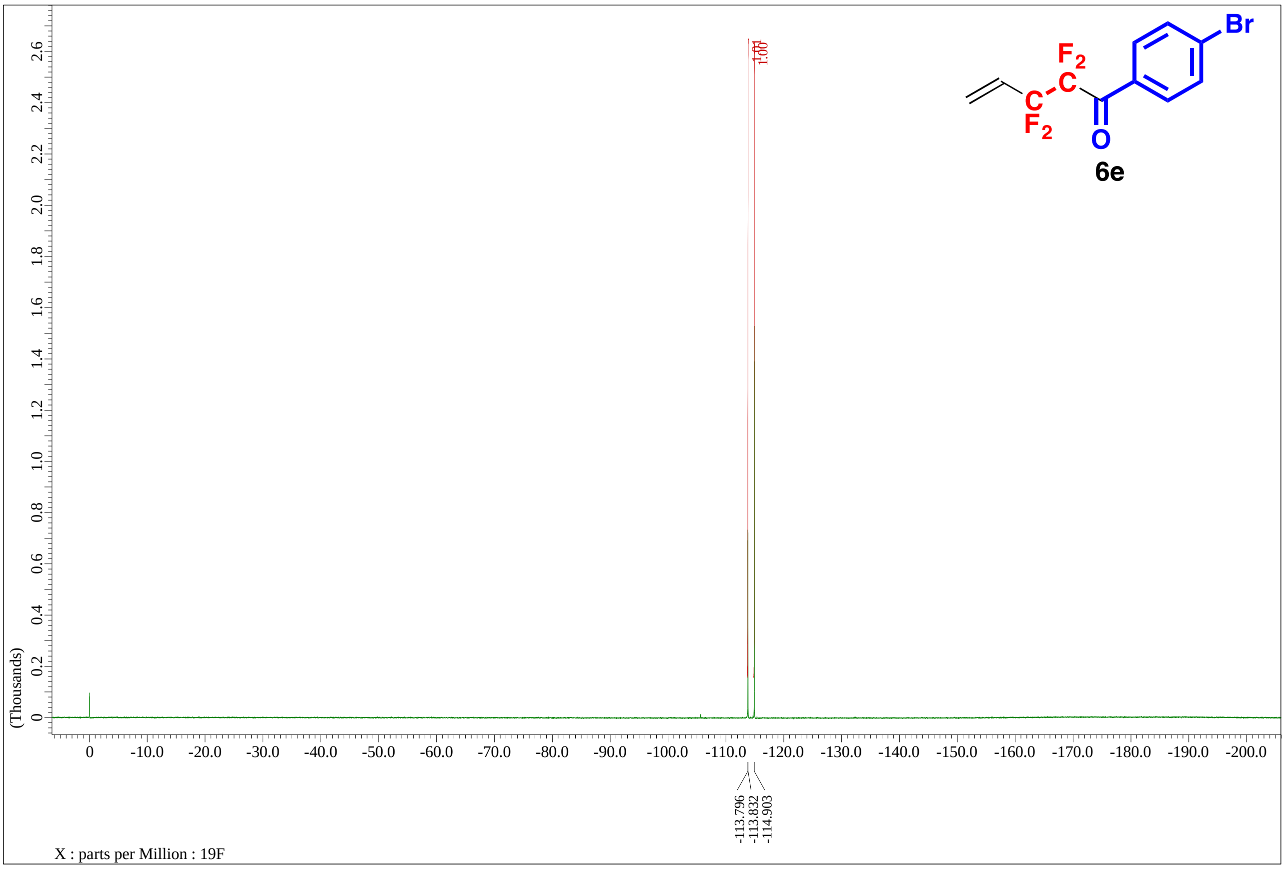

^{1}H NMR Spectrum of 2,2,3,3-tetrafluoro-1-(4-trifluoromethylphenyl)pent-4-en-1-one (**6f**)

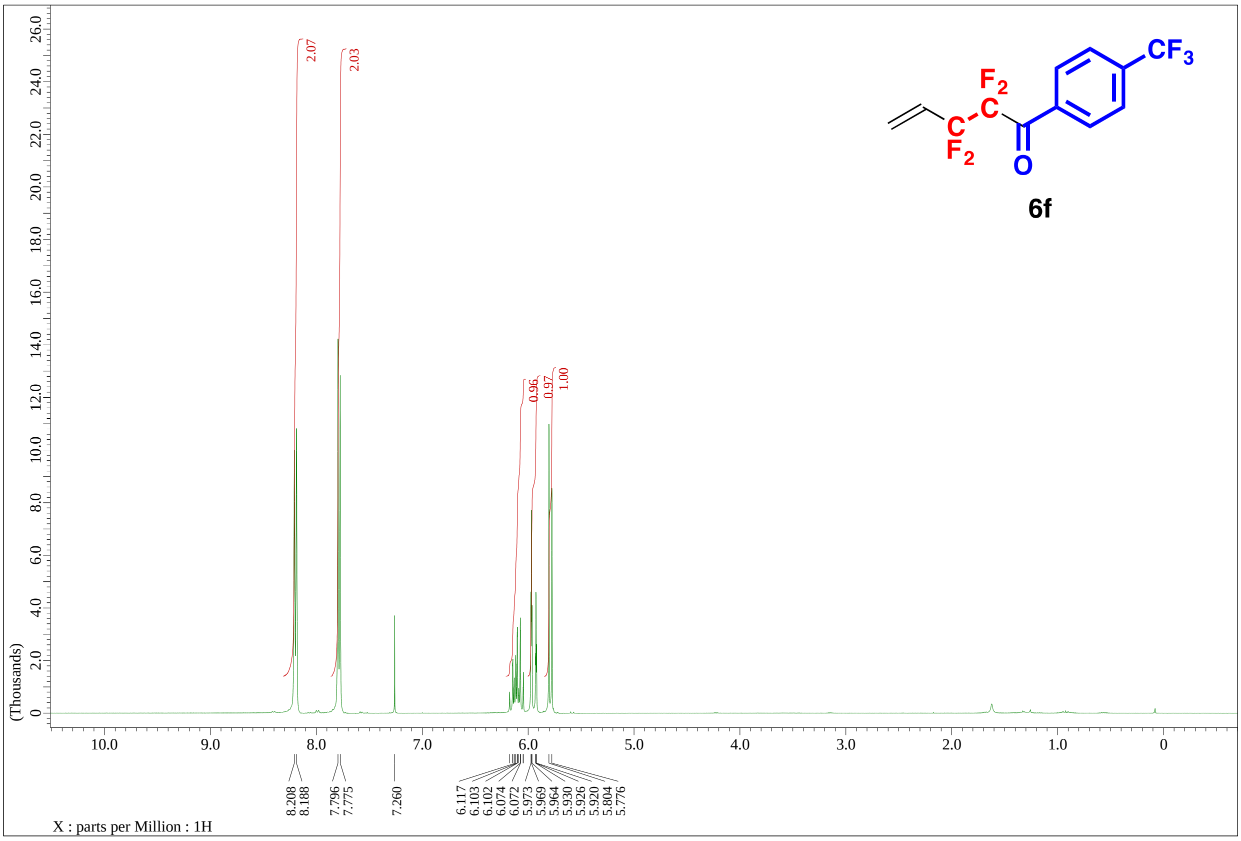

^{13}C NMR Spectrum of 2,2,3,3-tetrafluoro-1-(4-trifluoromethylphenyl)pent-4-en-1-one (**6f**)

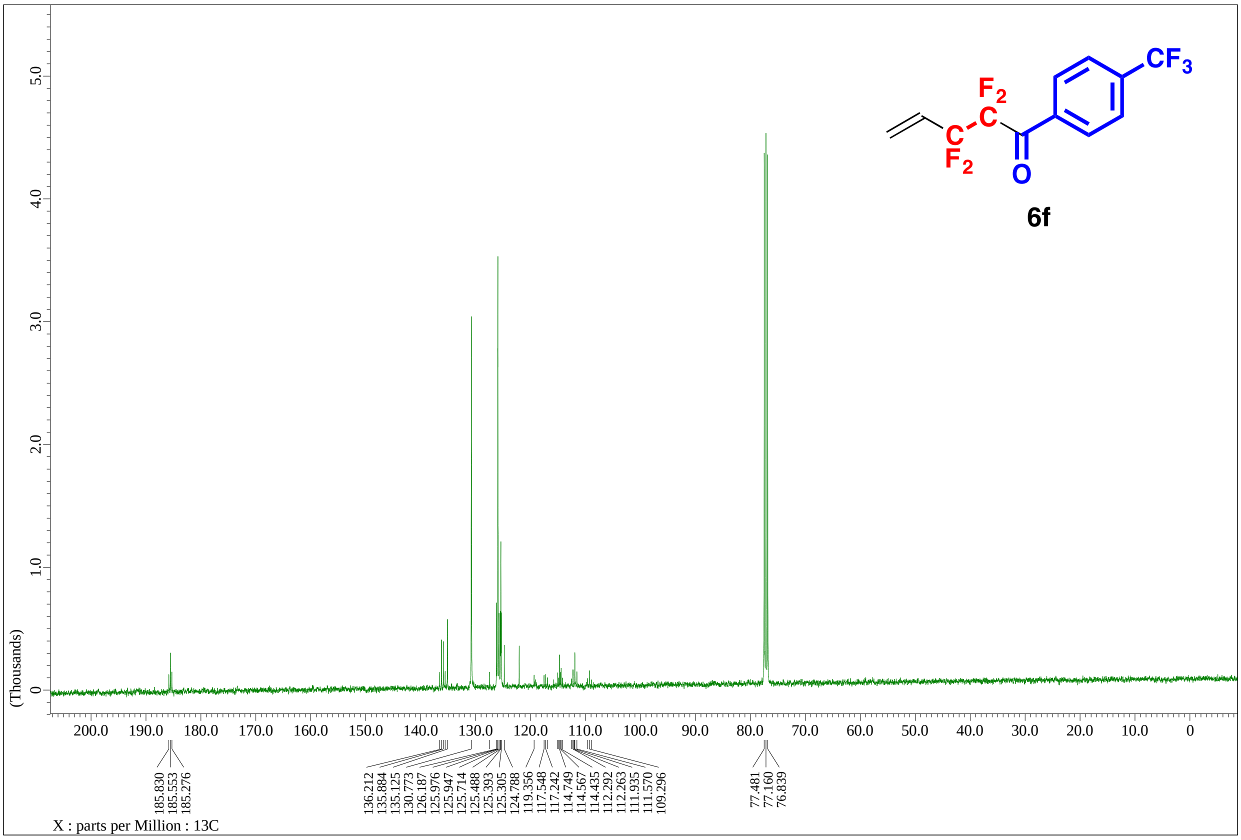

^{19}F NMR Spectrum of 2,2,3,3-tetrafluoro-1-(4-trifluoromethylphenyl)pent-4-en-1-one (**6f**)

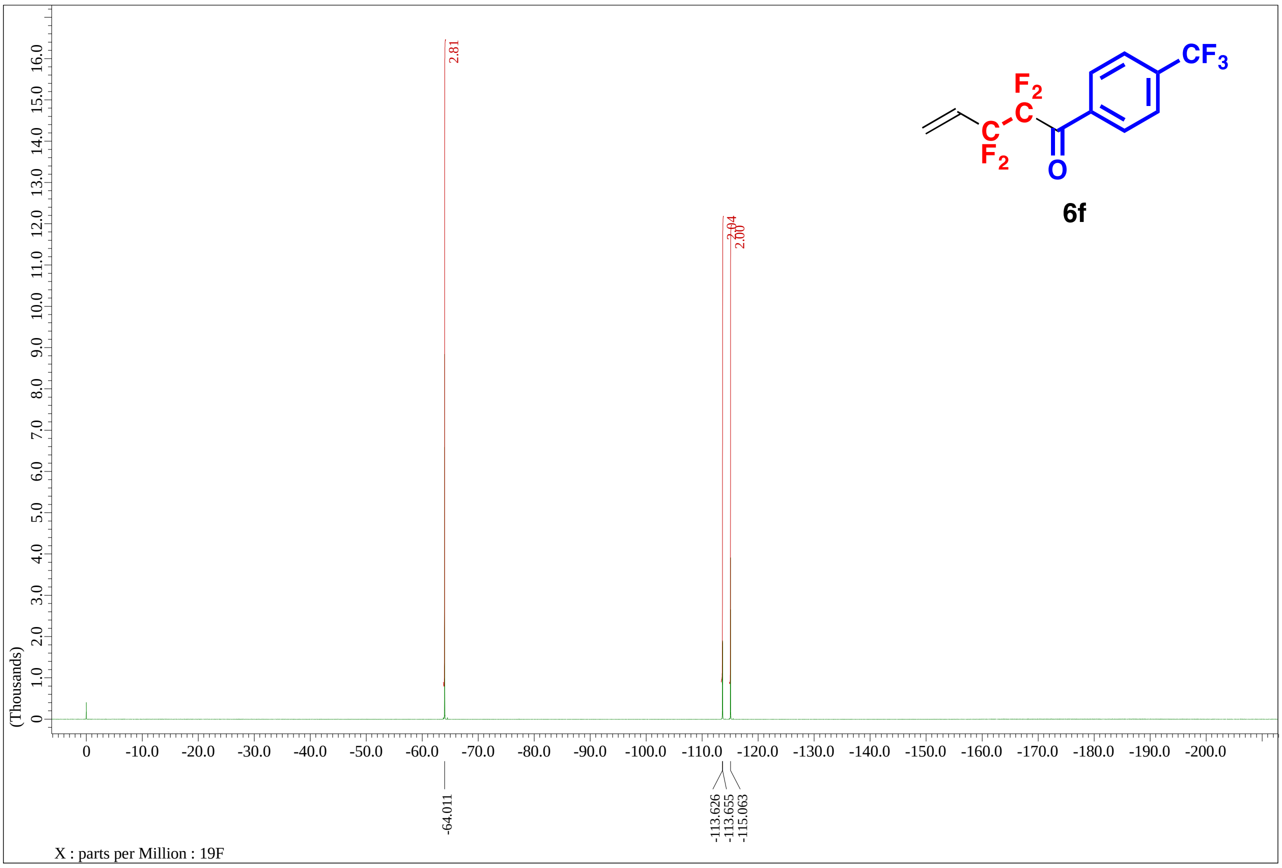

^{1}H NMR Spectrum of 2,2,3,3-tetrafluoro-1-(4-nitrophenyl)pent-4-en-1-one (**6g**)

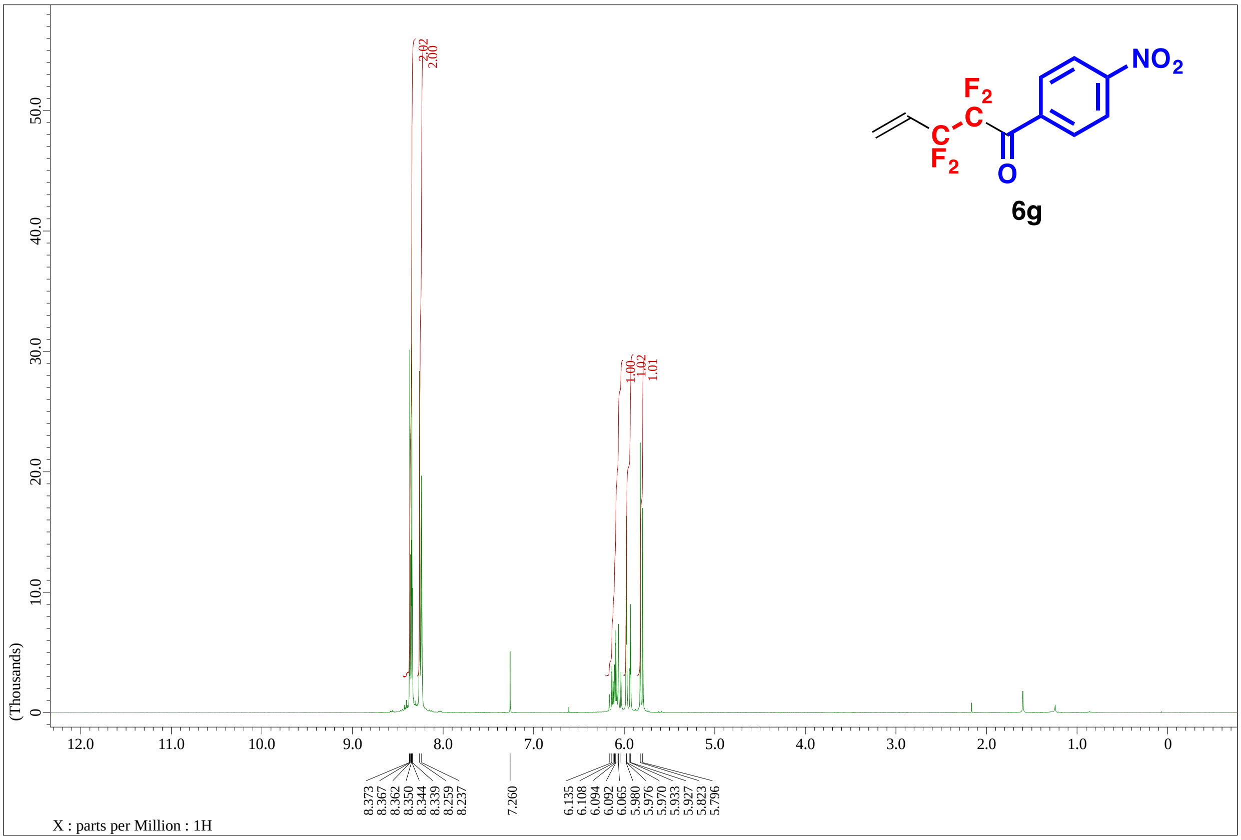

^{13}C NMR Spectrum of 2,2,3,3-tetrafluoro-1-(4-nitrophenyl)pent-4-en-1-one (**6g**)

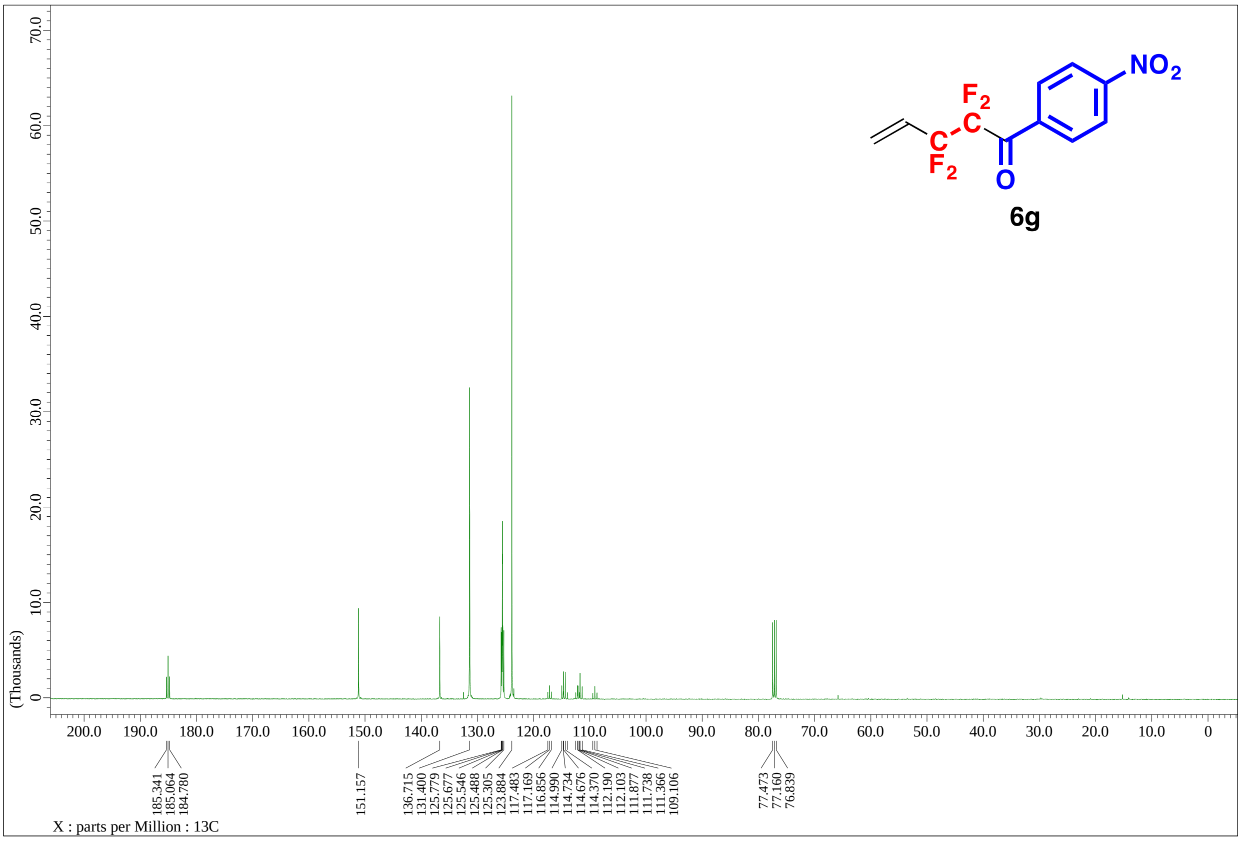

^{19}F NMR Spectrum of 2,2,3,3-tetrafluoro-1-(4-nitrophenyl)pent-4-en-1-one (**6g**)

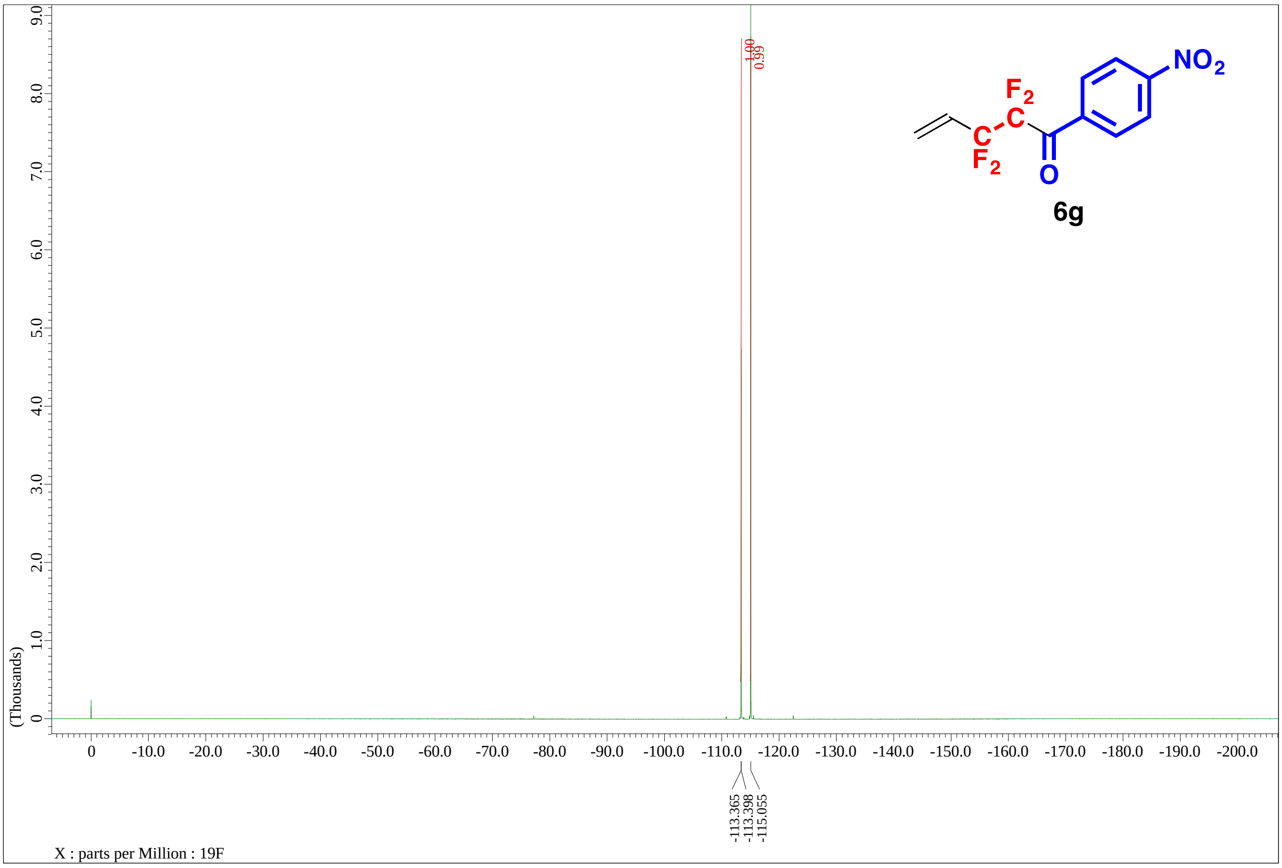

^{1}H NMR Spectrum of 2,2,3,3-tetrafluoro-1-(furan-2-yl)pent-4-en-1-one (**6h**)

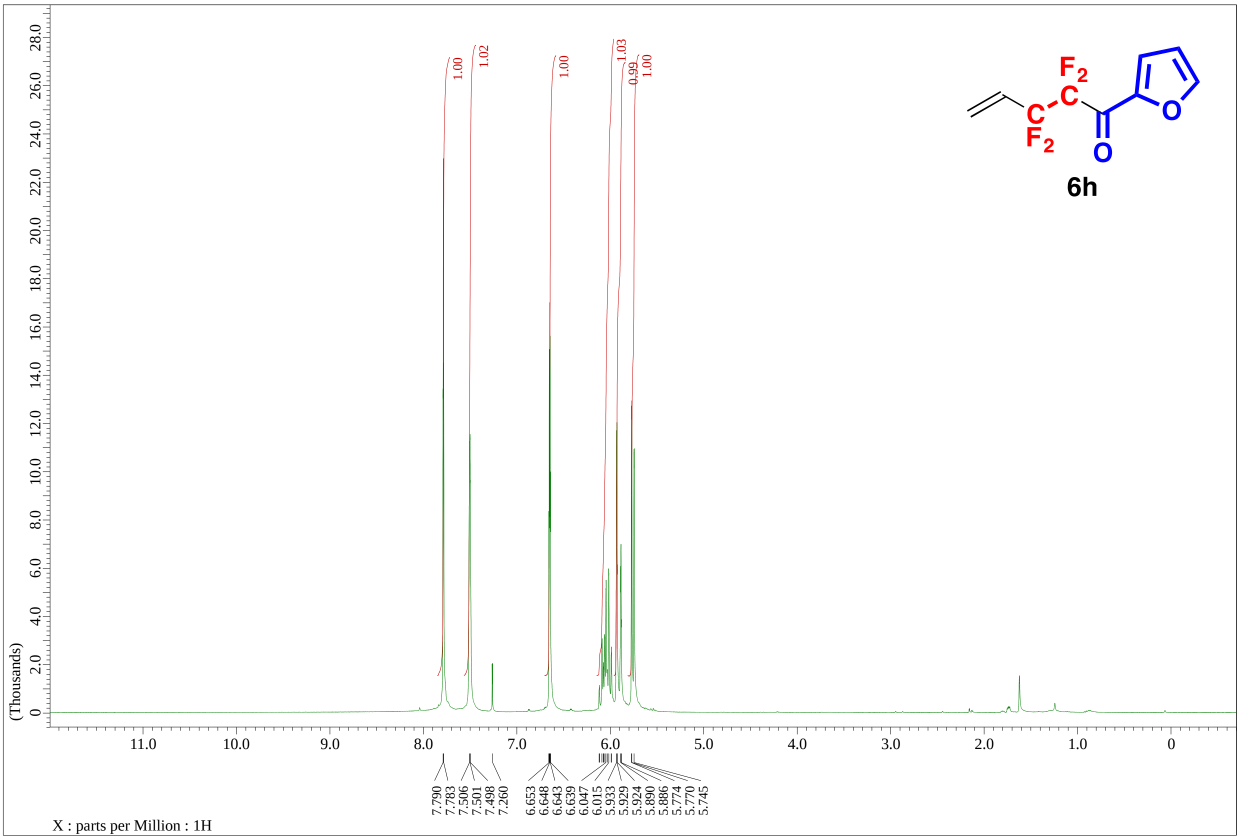

^{13}C NMR Spectrum of 2,2,3,3-tetrafluoro-1-(furan-2-yl)pent-4-en-1-one (**6h**)

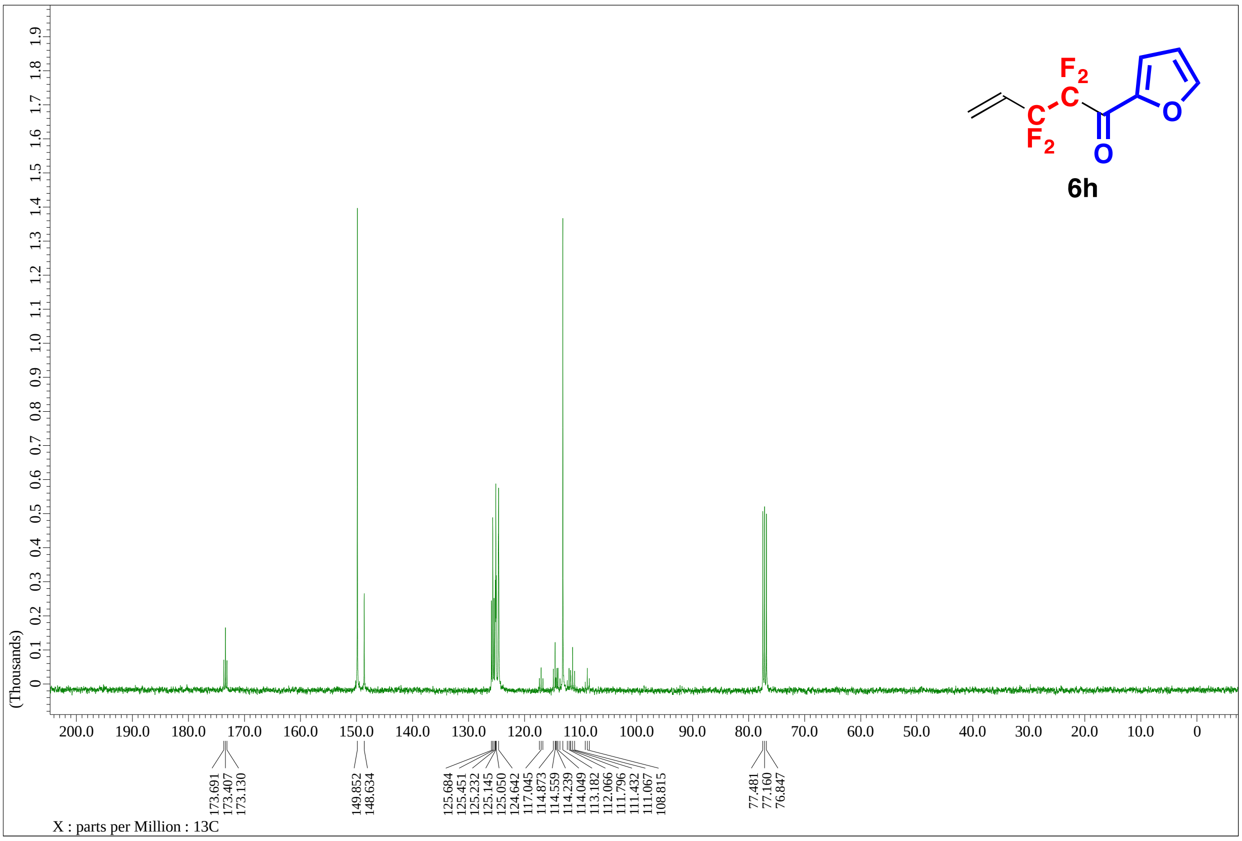

^{19}F NMR Spectrum of 2,2,3,3-tetrafluoro-1-(furan-2-yl)pent-4-en-1-one (**6h**)

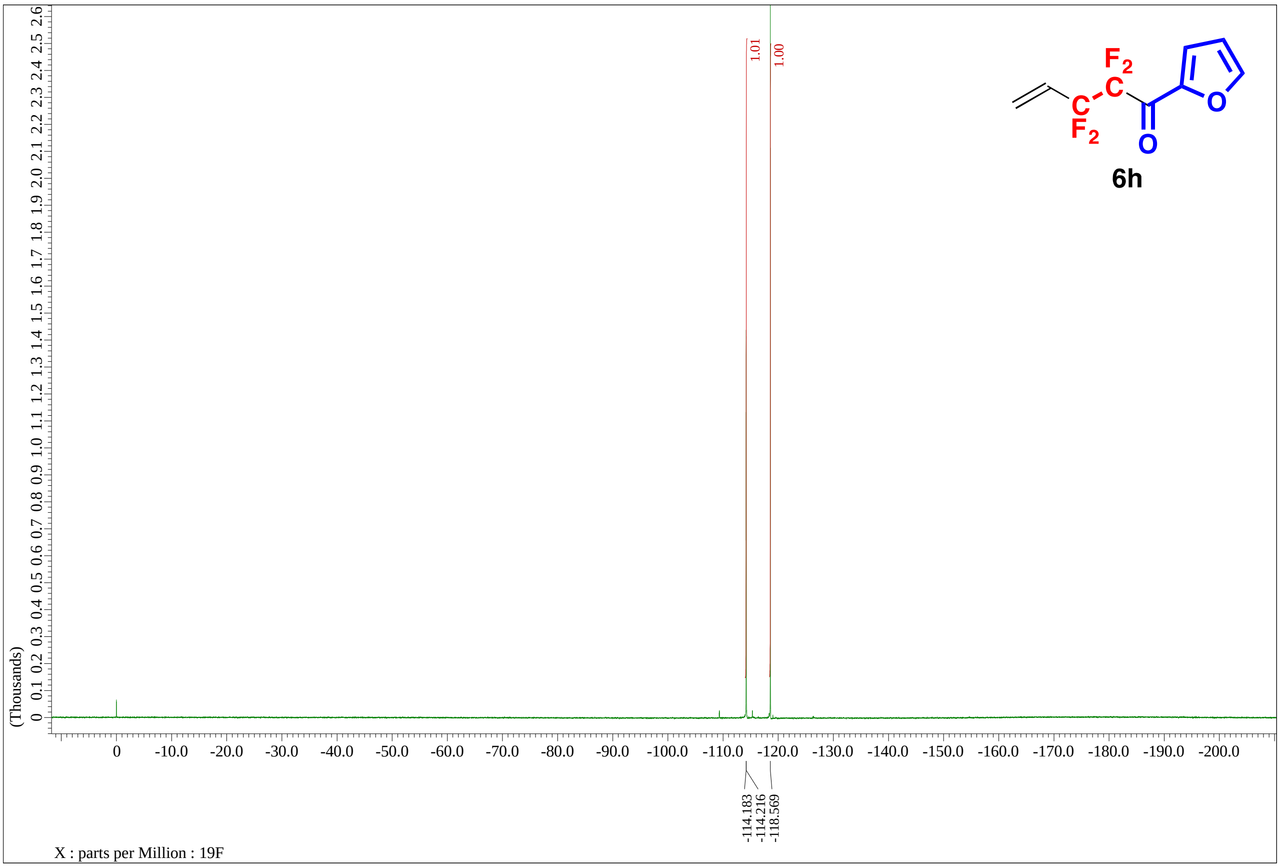

^{1}H NMR Spectrum of 2,2,3,3-tetrafluoro-1-(thiophen-2-yl)pent-4-en-1-one (**6i**)

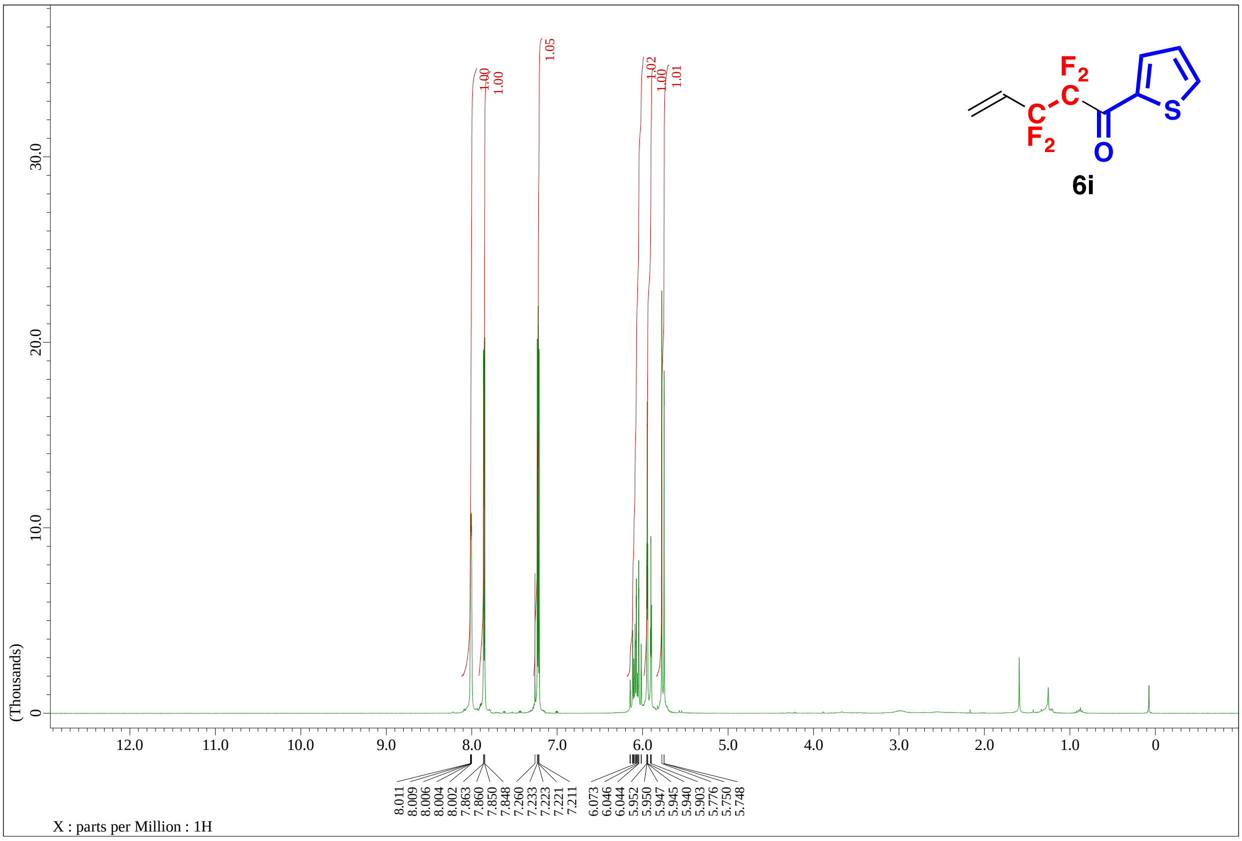

^{13}C NMR Spectrum of 2,2,3,3-tetrafluoro-1-(thiophen-2-yl)pent-4-en-1-one (**6i**)

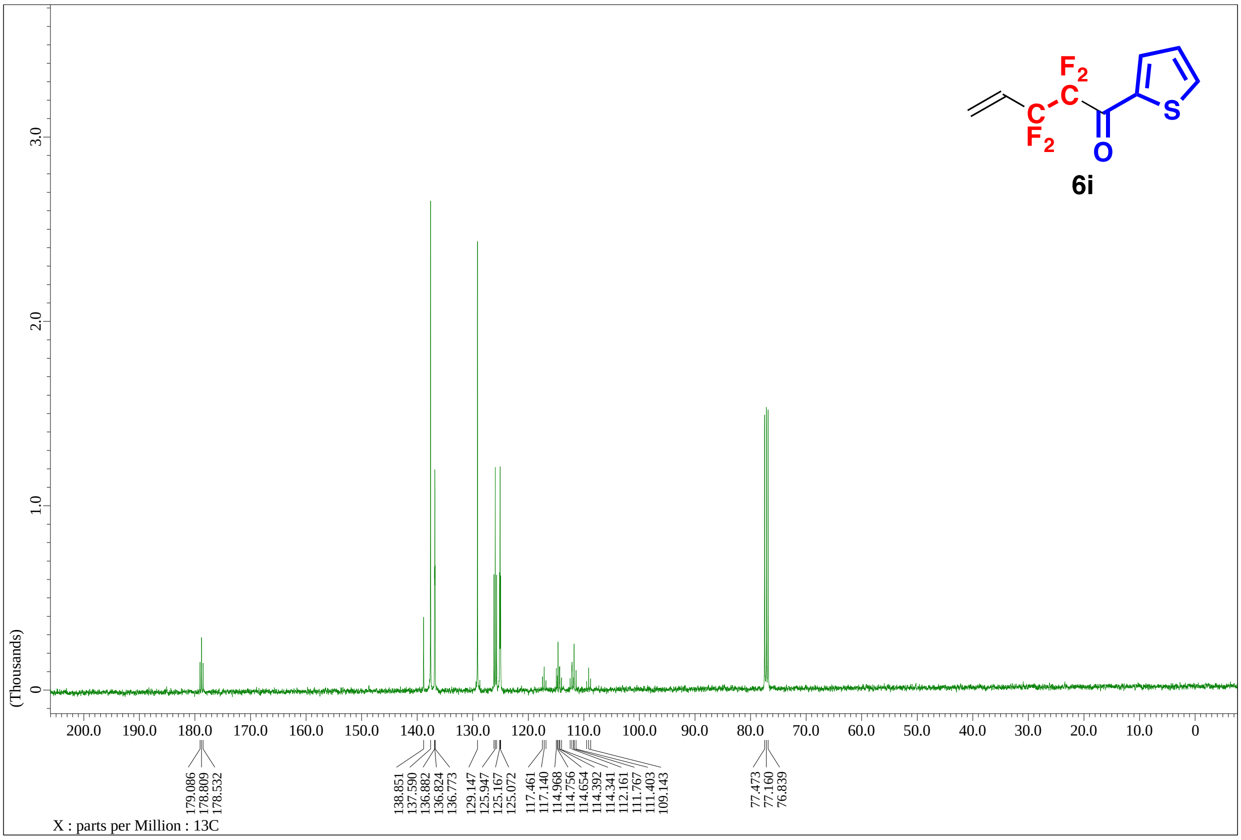

^{19}F NMR Spectrum of 2,2,3,3-tetrafluoro-1-(thiophen-2-yl)pent-4-en-1-one (**6i**)

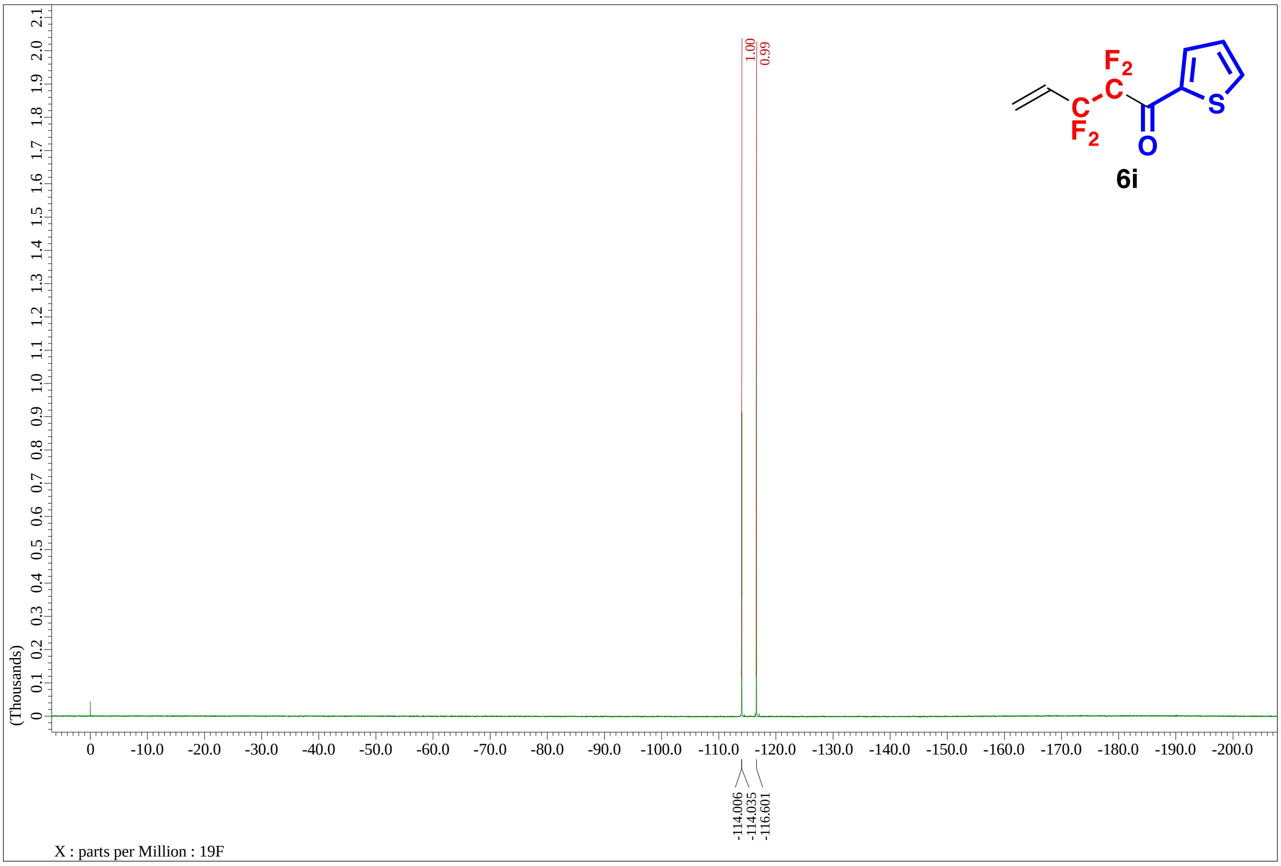

^{1}H NMR Spectrum of 4,4,5,5-tetrafluoro-1-phenylhept-1,6-dien-3-one (**6j**)

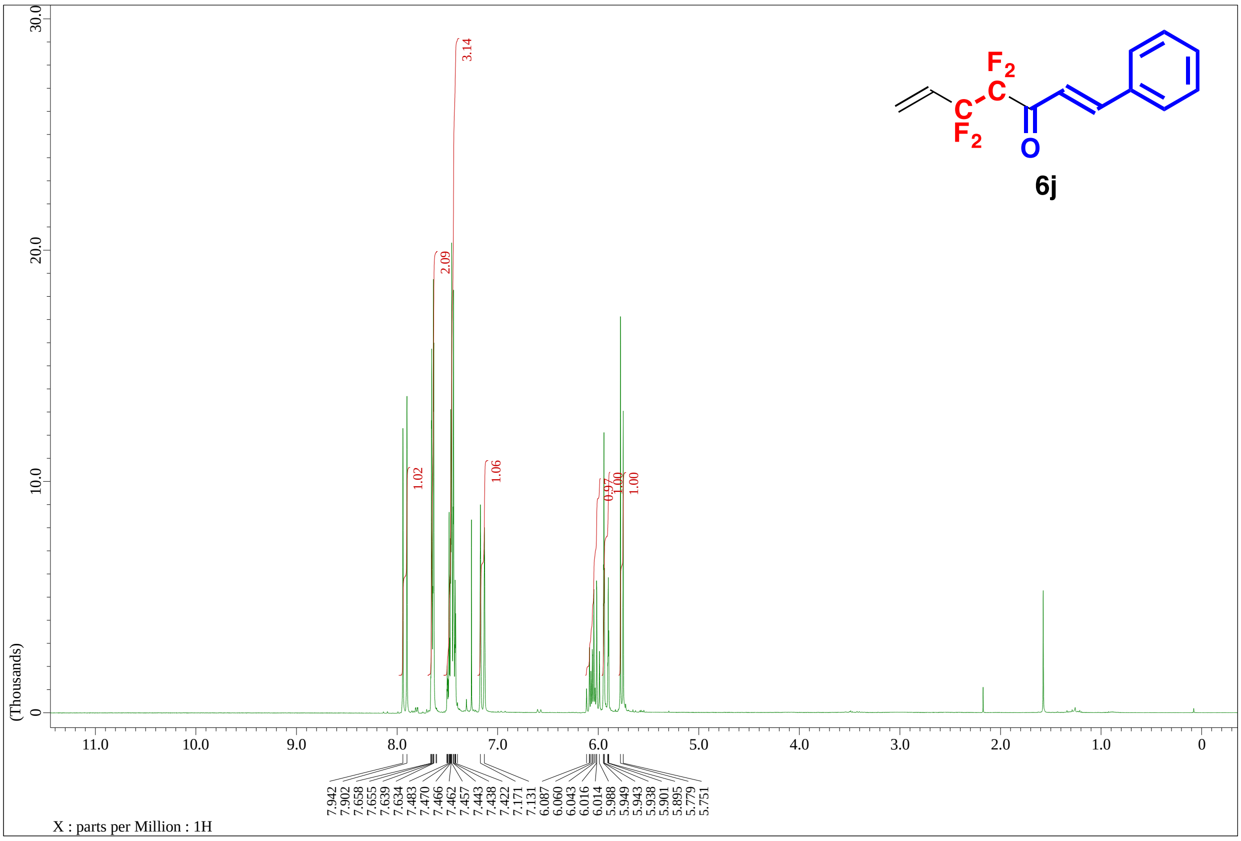

^{13}C NMR Spectrum of 4,4,5,5-tetrafluoro-1-phenylhept-1,6-dien-3-one (**6j**)

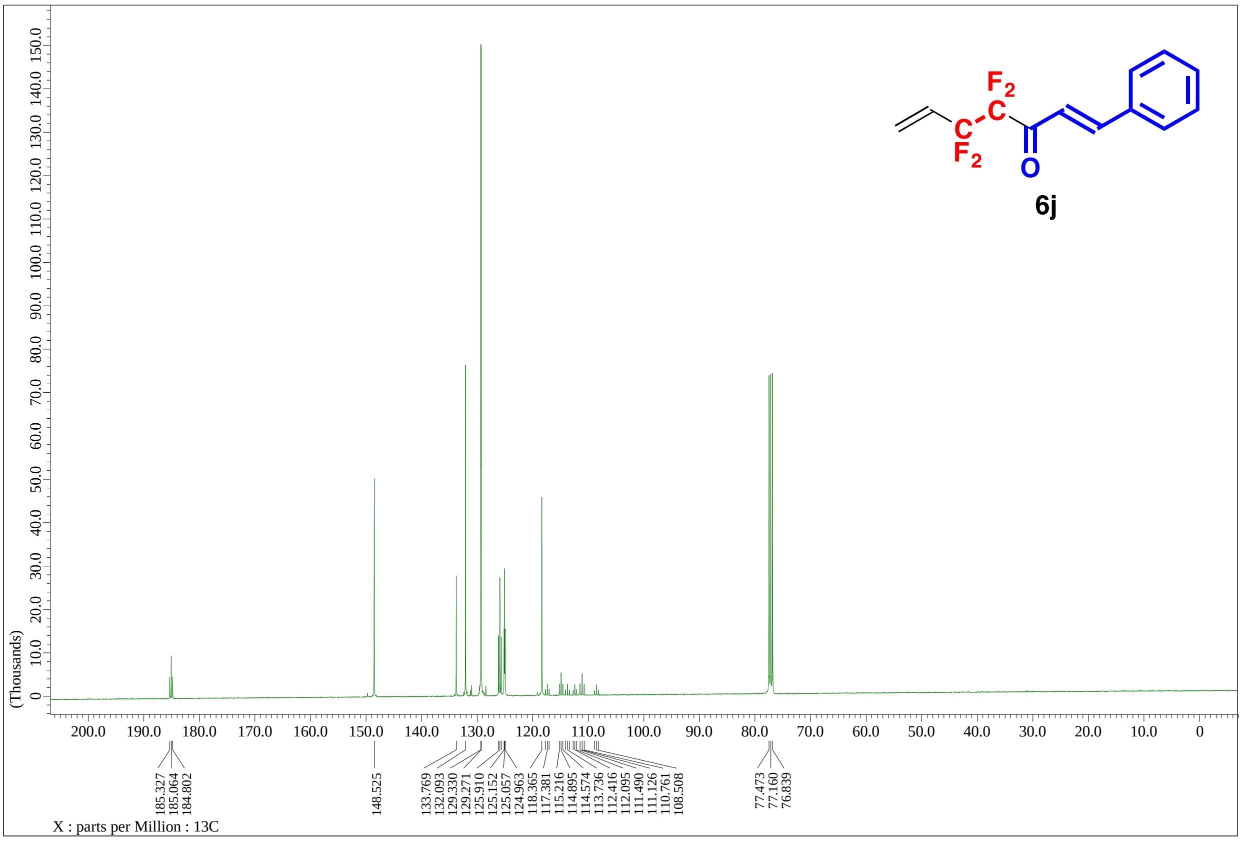

^{19}F NMR Spectrum of 4,4,5,5-tetrafluoro-1-phenylhept-1,6-dien-3-one (**6j**)

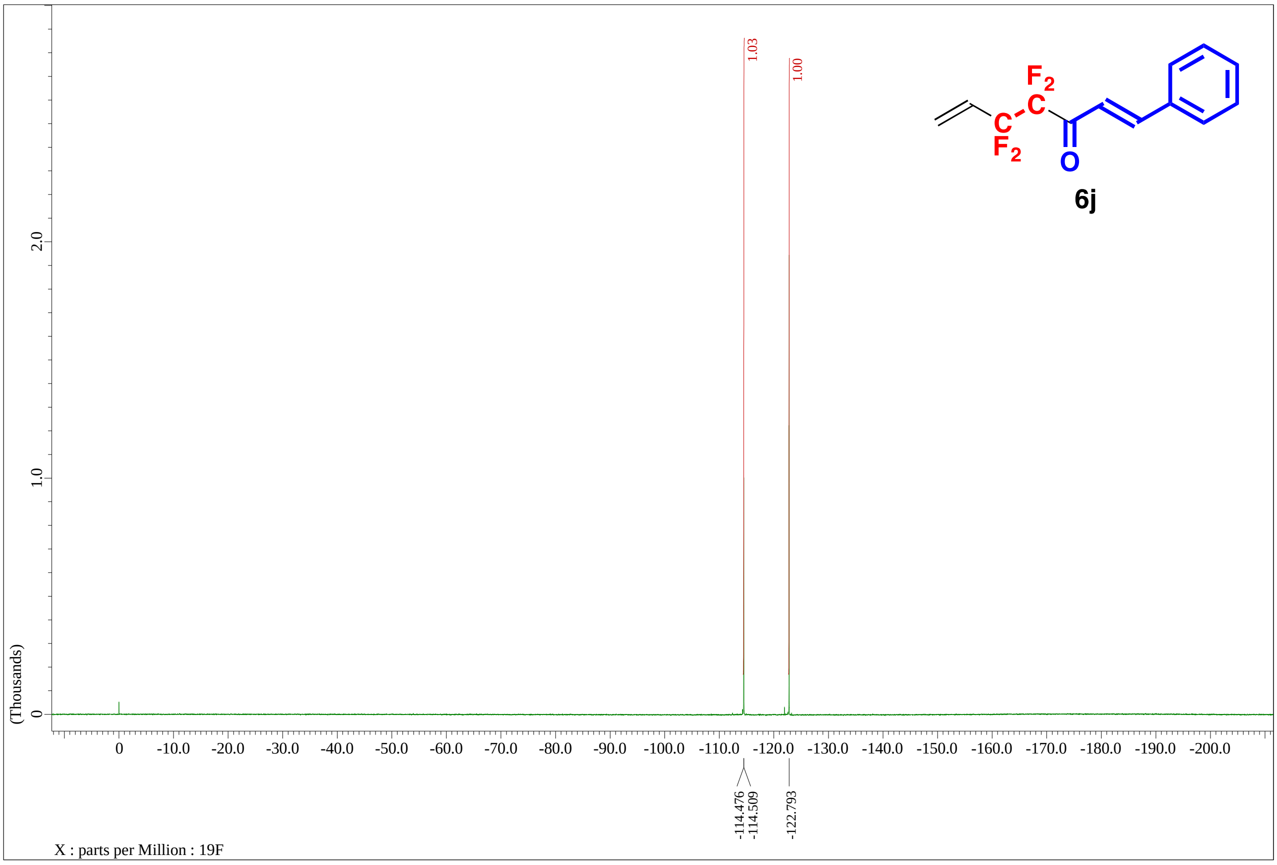

^{1}H NMR Spectrum of 3,3,4,4-tetrafluorododec-1-en-5-one (**6k**)

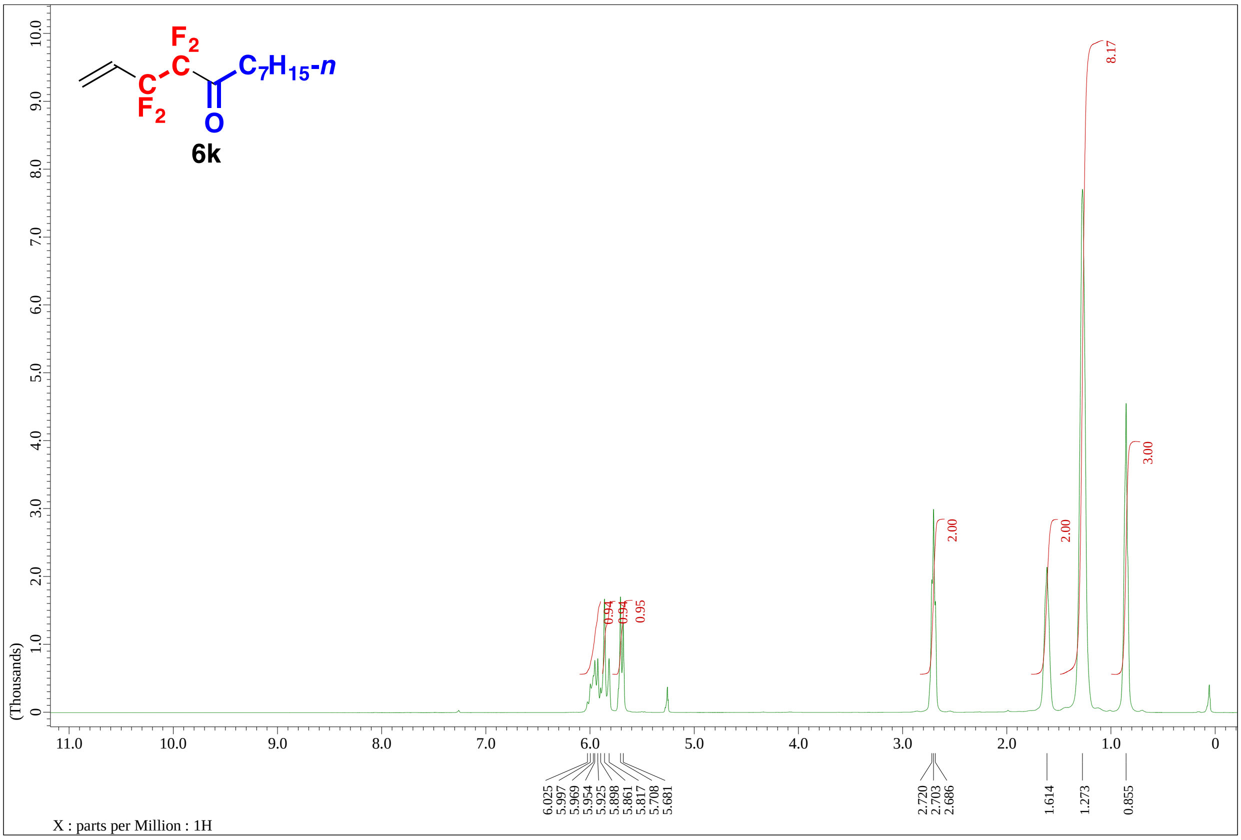

^{13}C NMR Spectrum of 3,3,4,4-tetrafluorododec-1-en-5-one (**6k**)

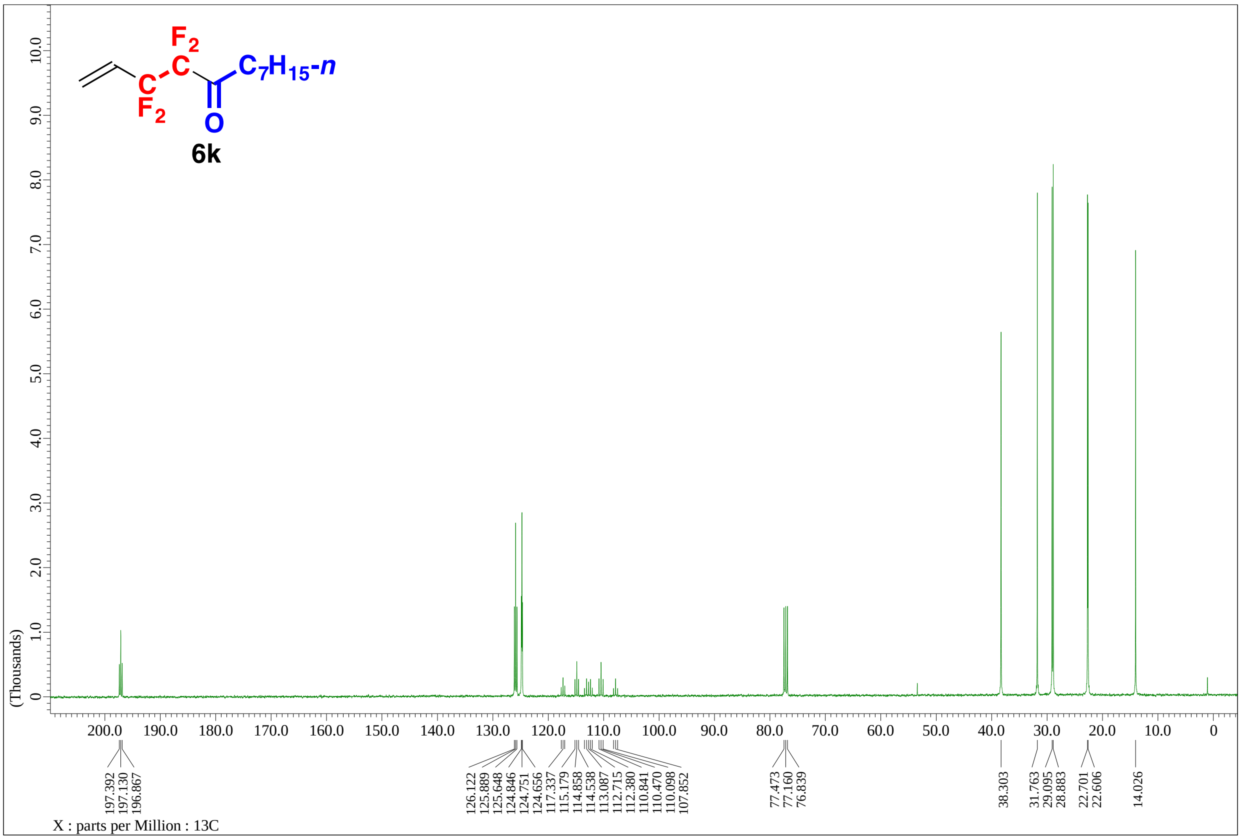

^{19}F NMR Spectrum of 3,3,4,4-tetrafluorododec-1-en-5-one (**6k**)

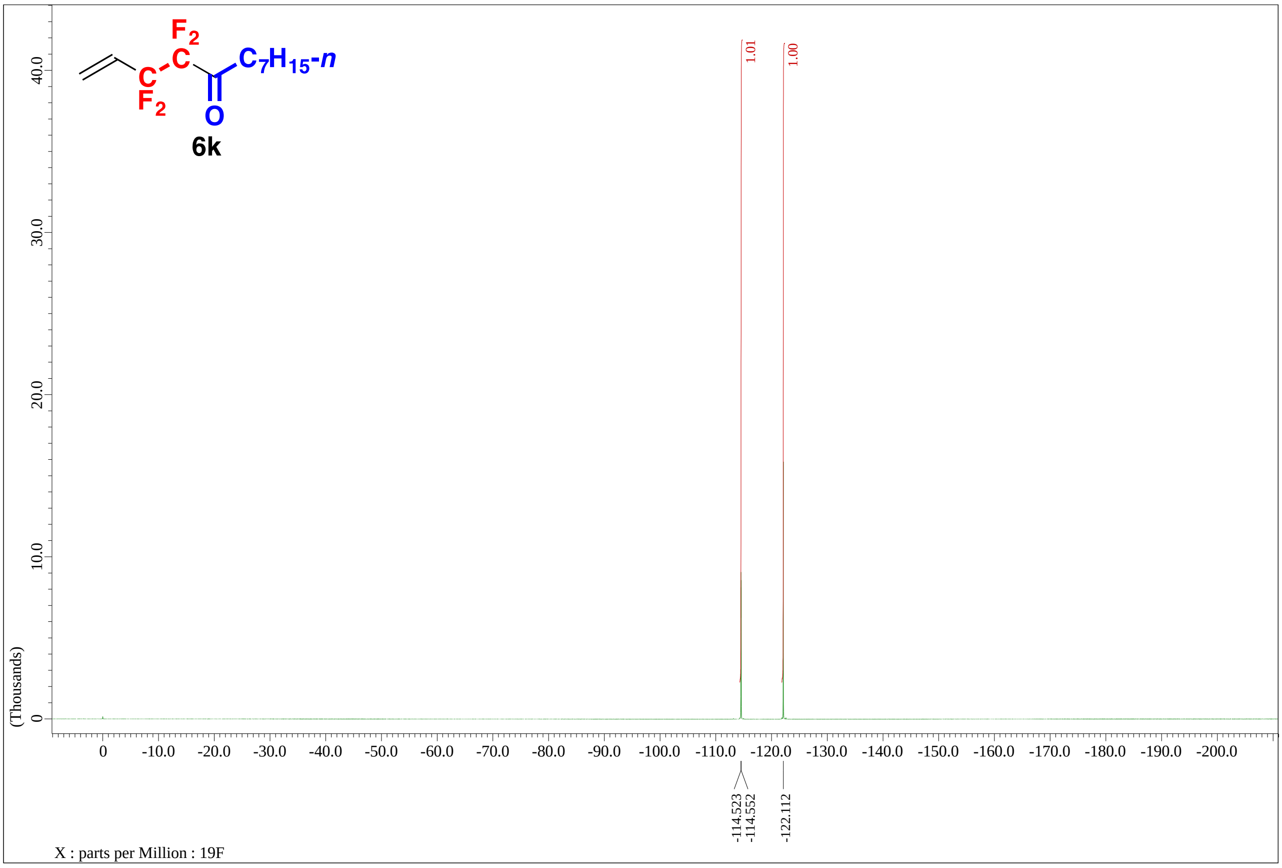

Supplement: File 1 — Experimental procedures, characterization data (1H, 13C, 19F NMR, IR and HRMS), copies of 1H, 13C and 19F NMR spectra. [file Beilstein_J_Org_Chem-14-2375-s001.pdf]
